# Supplementary material for: Enzymatic synthesis and nanopore sequencing of 12-letter supernumerary DNA
Source: Nat Commun. 2023 Oct 26;14:6820. doi: 10.1038/s41467-023-42406-z (PMC10603101; doi:10.1038/s41467-023-42406-z)
Supplement: Supplementary file 1 — Supplmentary Information [file 41467_2023_42406_MOESM1_ESM.pdf]

Supplementary Information for

Enzymatic Synthesis and Nanopore Sequencing of 12-letter Supernumerary DNA

H. Kawabe, C. A. Thomas, S. Hoshika, M.-J. Kim, M.-S. Kim, L. Miessner, N. Kaplan, J. M. Craig, J. H. Gundlach, A. H. Laszlo, S. A. Benner, J. A. Marchand

Supplementary Methods

Organic synthesis of dX^{t}TP: 8-(2'-Deoxy-β-D-erythro-pentofuranosyl)imidazo[1,2-a]-s-triazin-2,4-dione 5'-triphosphate S4
Organic synthesis of dK^{n}TP: 5-(2'-Deoxy-β-D-ribofuranosyl)-2,6-diamino-3-nitropyridine 5'-triphosphate S6
Organic synthesis of dJTP: 4-Amino-8-(2'-deoxy-β-D-erythro-pentofuranosyl)imidazo[1,2-a]-1,3,5-triazin-2-one 5'-triphosphate S9
Organic synthesis of dVTP: 2-Amino-5-nitro-3-(2'-deoxy-β-D-ribofuranosyl)-1H-pyridin-6-one-5'-triphosphate S13

Supplementary Notes

Supplementary Note 1. Xenonucleotide substitution basecalling with Xenomorph S16
Supplementary Note 2. Per-read recall of simulated reads for artificial genetic codes S16

Supplementary Results

Supplementary Table 1. Full description and abbreviations of XNA nucleobases used in this work S17
Supplementary Table 2. Synthetic hairpin sequences S17
Supplementary Table 3. Synthetic hairpin NNN-oligo pools and hairpin 20mer-validation pools S18
Supplementary Table 4. Library pool-barcodes sequences S18
Supplementary Table 5. Triplet-barcodes sequences S19
Supplementary Table 6. 20-mer validation library sequences S20
Supplementary Table 7. 12-letter DNA construction sequences S21
Supplementary Table 8. Optimized reaction components and conditions used for XNA tailing S21
Supplementary Table 9. XNA tailing extent of reaction S22
Supplementary Table 10. Optimized reaction components and conditions used for XNA ligation S22
Supplementary Table 11. XNA ligation yield S22
Supplementary Table 12. Constructs generated through XNA tailing S23
Supplementary Table 13. Constructs generated through XNA ligation S24
Supplementary Table 14. Nanopore run overview S25
Supplementary Table 15. Nanopore run read summary for model building S25
Supplementary Table 16. Heptamer, kmer, and level structure used in kmer model S26
Supplementary Table 17. Column header and description of xenomorph preprocess output file S27
Supplementary Table 18. Column header and description of xenomorph morph output file S27
Supplementary Table 19. Recall benchmarking for 4-nt kmer XNA models S28
Supplementary Table 20. Specificity benchmarking for 4-nt kmer XNA models S28

Supplementary Table 21. Recall benchmarking for 4-nt and 12-letter kmer XNA model	S29
Supplementary Table 22. Template sequences and primer sequences used for PCR of P≡Z base pair	S30
Supplementary Table 23. Thermocycler settings used for PCR of P≡Z base pair	S30
Supplementary Table 24. 12-letter DNA Sequence (S^cuper-12 and S^nuper-12)	S31
Supplementary Table 25. S^cuper-12 per-read recall confusion matrix values	S32
Supplementary Table 26. S^nuper-12 per-read recall confusion matrix values	S32
Supplementary Table 27. Tabulation of per-read recall from simulated signal levels for the standard genetic code (A, T, G, C)	S33
Supplementary Table 28. Tabulation of per-read recall from simulated signal levels for the isoG/isoC code (A, T, G, C, B, S^n)	S33
Supplementary Table 29. Tabulation of per-read recall from simulated signal levels for the hachimoji code (A, T, G, C, B, S^c, P, Z)	S33
Supplementary Table 30. Tabulation of per-read recall from simulated signal levels for the 12-base S^nupernumerary code (A, T, G, C, B, S^n P, Z, X^t, K^n, J, V)	S34
Supplementary Table 31. Nanopore datasets of supernumerary DNA uploaded to Sequence Reads Archive	S35
Supplementary Figure 1. Overview of non-templated N+1 tailing reaction	S36
Supplementary Figure 2. Screening polymerases capable of effective tailing of both purine and pyrimidine dNTPs for canonical bases (N = A, T, G, C) by T4 ligation assay	S37
Supplementary Figure 3. Synthesis of dX^t triphosphate	S38
Supplementary Figure 4. Synthesis of dK^n triphosphate	S39
Supplementary Figure 5. Synthesis of dJ triphosphate	S40
Supplementary Figure 6. Synthesis of dV triphosphate	S41
Supplementary Figure 7. UPLC/QTOF validation of tailing activity for all dNTPs and dxNTPs by Klenow Fragment (exo-)	S42
Supplementary Figure 8. UPLC/QTOF validation of tailing activity for all dNTPs and dxNTPs by Therminator	S44
Supplementary Figure 9. Screening and optimization of XNA tailing conditions	S46
Supplementary Figure 10. Addition of yeast inorganic pyrophosphatase (YiPP) leads to slight improvements in XNA tailing reaction yield	S47
Supplementary Figure 11. Enzymatic tailing does not lead to measurable differences in ligation when compared to ligation using fully synthetic hairpin with N+1 tails	S48
Supplementary Figure 12. High resolution LC/MS of oligo showing N+1 tailing as major product	S49
Supplementary Figure 13. Overview of T3 DNA ligase, T4 DNA ligase, and T7 DNA ligase products	S52
Supplementary Figure 14. Overview of XNA ligation products from XNA tailed hairpins	S53
Supplementary Figure 15. Screening and optimization of ligation conditions across all XNA bases	S54
Supplementary Figure 16. Screening T3 ligase, T4 ligase, T7 ligase for JV, X^tK^n, and BS^c XNA ligation	S56
Supplementary Figure 17. Full gels of XNA tailing and XNA ligation using optimized conditions	S58
Supplementary Figure 18. Proof of concept for XNA tailing and XNA ligation cycling to insert two consecutive P≡Z base pairs	S59
Supplementary Figure 19. Examples of basecalling XNA sequences with guppy	S61
Supplementary Figure 20. Full gels of NNN<u>N</u>NNN library construction for nanopore sequencing	S62
Supplementary Figure 21. Variance minimization for segmentation steps of signal-to-sequence mapping	S63
Supplementary Figure 22. Example trace of signal deviation from the standard model	S64
Supplementary Figure 23. Distribution of observed 4-nt kmers signal levels for all XNA bases	S65
Supplementary Figure 24. Example comparison of observed 4-nt kmer signal levels across a full heptamer sequence	S75

Supplementary Figure 25. Full heatmap showing measured 4-nt kmer model KDE means including standard bases S76
Supplementary Figure 26. Full heatmap showing measured 4-nt XNA kmer model deviation from standard model S77
Supplementary Figure 27. The Xenomorph preprocessing pipeline S78
Supplementary Figure 28. Construction, pooling, and sequencing of the Val-20 6-letter DNA libraries S79
Supplementary Figure 29. PCR amplification and sequencing of a DNA template with a P≡Z base pair S80
Supplementary Figure 30. Construction of 12-letter DNA for nanopore sequencing S81
Supplementary Figure 31. Confusion matrix values of per-read recall for simulated reads S82
Supplementary Figure 32. Box plots of per-read recall for simulated reads S83

Supplementary References S84

Supplementary Methods

Organic synthesis of dX^{t}TP: 8-(2′-Deoxy-β-D-erythro-pentofuranosyl)imidazo[1,2-a]-s-triazin-2,4-dione 5′-triphosphate. Schematic of the synthesis is shown in **Supplementary Fig. 3**.

8-[2′-Deoxy-3′,5′-di-*O*-(*p*-toluoyl)-β-D-erythro-pentofuranosyl]-2-[(2-methylpropionyl)amino]-imidazo[1,2-*a*]-s-triazin-4-one (3). 5-Aza-7-deazaguanine **2** (8 g, 51.9 mmol) was dissolved in 10% aqueous K_2CO_3 solution (400 mL) and a solution of Bu_4NHSO_4 (1.28 g, 3.77 mmol) in CH_2Cl_2 (280 mL) was added at room temperature. After vigorous stirring for more than 1 min, a solution of 2′-deoxy-di-*O*-(p-toluoyl)-α-D-erythro-pentofuranosyl chloride **1** (21.4 g, 55.0 mmol) in CH_2Cl_2 (180 mL) was added at room temperature. The reaction mixture was stirred for 1 h at room temperature and the organic layer was separated. The aqueous layer was extracted with CH_2Cl_2 (500 mL × 2). The combined organic layer was dried (Na_2SO_4), filtered and concentrated. The residue was dissolved in pyridine (300 mL) and DMAP (1.12 g) and isobutyryl chloride (6 mL) were added at room temperature. After stirring overnight at room temperature, the reaction mixture was concentrated and the residue was dissolved in hot MeOH (150 mL). This mixture was stored at 0 °C for 3 h and the resulting precipitate was filtered and washed with cold MeOH (50 mL) to give the β-isomer **3** (5.9 g, 10.3 mmol, 19%) as a white solid.

^{1}H NMR(DMSO-d_6, 300 MHz) δ10.39 (s, 1H), 7. 90 (d, 2H, J=8.1), 7.81 (d, 2H, J=8.1), 7.70 (d, 1H, J=2.7), 7.60 (d, 1H, J=2.4), 7.34 (d, 2H, J=8.1), 7.26 (d, 2H, J=8.1), 6.36 (t, 1H, J=6.6), 5.74 (m, 1H), 4.50-4.64 (m, 3H), 3.13 (m, 1H), 2.81 (m, 1H), 2.68 (m, 1H), 2.38, 2.34 (2s, 6H), 1.03, 1.01 (2s, 6H).

2-Amino-8-(2′-Deoxy-β-D-erythro-pentofuranosyl)imidazo[1,2-*a*]-s-triazin-4-one (4). To a stirred suspension of **3** (5 g, 8.7 mmol) in MeOH (80 mL) was added 40 % methylamine solution (5 mL) and stirred for 2 days at room temperature. After removal of solvent, ethanol/ether mixture was added to the residue. The resulting precipitate was filtered and dried to give **4** (2.1 g, 7.9 mmol, 90%) as a white solid.

^{1}H NMR(DMSO-d_6, 300 MHz) δ7.42 (d, 1H, J=2.4), 7.34 (d, 1H, J=2.4), 6.93(brs, 2H), 6.13 (t, 1H, J = 6.6), 5.30 (brs, 1H), 4.98 (m, 1H), 4.29 (m, 1H), 3.78 (m, 1H), 3.50 (m, 2H), 2.32 (m, 1H), 2.12 (m, 1H).

8-(2′-Deoxy-β-D-erythro-pentofuranosyl)imidazo[1,2-*a*]-s-triazin-2,4-dione (5). **4** (1.34 g, 5 mmol) was dissolved in acetic acid (38 mL) and a solution of $NaNO_2$ (2 g) in H_2O (6 mL) was added at room temperature. The reaction mixture was stirred at room temperature for 2 days and concentrated. The residue was purified by silica gel column chromatography (CH_2Cl_2: MeOH=8: 1 to 4: 1) to give dX^t nucleoside **5** (900 mg, 3.36 mmol, 67%) as a white solid.

^{1}H NMR(DMSO-d_6, 300 MHz) δ7.45 (d, 1H, J=3.0), 7.41 (d, 1H, J=3.0), 6.05 (t, 1H, J=6.6), 4.28 (m, 1H), 3.79 (m, 1H), 3.46-3.58 (m, 2H), 2.28 (m, 1H), 2.13 (m, 1H).

8-[2′-Deoxy-5′-*O*-(4,4′-dimethoxytripenylmethyl)-β-D-erythro-pentofuranosyl]imidazo[1,2-a]-triazine-2,4-dione (6). To a stirred solution of **5** (469 mg, 1.75 mmol) in pyridine (25 mL) was added DMTCl (652 mg, 1.92 mmol) at room temperature. The reaction mixture was stirred overnight at room

temperature and evaporated. The residue was purified by silica gel column chromatography (EtOAc to EtOAc: MeOH=9: 1) to give **6** (580 mg, 1.02 mmol, 58%) as a white solid.

^{1}H NMR(DMSO-d_6, 300 MHz) δ 11.26 (s, 1H), 7.42 (d, 1H, J=2.4), 7.18-7.35 (m, 10H), 6.83 (m, 4H), 6.09 (t, 1H, J=6.3), 5.37 (d, 1H, J=4.5), 4.28 (m, 1H), 3.90 (M, 1H), 3.72 (S, 6H), 3.12 (m, 2H), 2.39 (m, 1H), 2.18 (m, 1H).

8-(3′-*O*-Acetyl-2′-deoxy-β-D-erythro-pentofuranosyl)imidazo[1,2-a]-triazine-2,4-dione (7). To a stirred solution of **6** (580 mg, 1.02 mmol) in pyridine (15 mL) was added Ac_2O (144 µL, 1.53 mmol) at room temperature. After being stirred at room temperature overnight, the reaction mixture was evaporated. The residue was treated with 3% dichloroacetic acid in CH_2Cl_2 (25 mL) for 2 h. Solvents were removed and the residue was purified by silica gel column chromatography (CH_2Cl_2: MeOH=7: 1) to give **7** (250 mg, 0.81 mmol, 79%) as a white solid.

^{1}H-NMR(300 MHz, DMSO-d_6): δ 11.23 (s, 1H), 7.45 (d, 1H, J=2.7), 7.42 (d, 1H, J=2.4), 6.04 (m, 1H), 5.23 (m, 2H), 4.01 (m, 1H), 3.56 (m, 2H), 2.52 (m, 1H), 2.31 (m, 1H), 2.04 (s, 3H).

8-(2′-Deoxy-β-D-erythro-pentofuranosyl)imidazo[1,2-a]-s-triazin-2,4-dione 5′-triphosphate (8). To a solution of **7** (309 mg, 1 mmol) in pyridine (4 mL) and dioxane (3.4 mL) was added a solution of 2-chloro-4*H*-1,3,2-benzodioxaphosphorin-4-one (260 mg) in dioxane (2.6 mL) at RT. After 20 min a mixture of tributylammonium pyrophosphate in DMF (0.2 M, 10 mL, 2 mmol) and tributylamine (1.2 mL, 4.8 mmol) was added. After 20 min a solution of iodine (360 mg) and water (0.56 mL) in pyridine (28 mL) was added. After 30 min the reaction was quenched by the addition of aqueous Na_2SO_3 (5%, 1 mL). The solvents were removed *in vacuo*. The residue was treated with NH_4OH (20 mL) for 3 h at room temperature and the mixture was lyophilized. The residue was dissolved in water (50 mL), and the mixture was filtered (0.2 µm). Purification by reverse phase HPLC (Sunfire Prep C_{18} column, 5 µm, 30 x 250 mm, eluent A = 25 mM TEAA pH 7, eluent B = CH_3CN in A, gradient from 0 to 40% B in 20 min, flow rate = 15 mL/min), followed by ion-exchange HPLC (Dionex BioLC DNAPac PA-100, 22 x 250 mm, eluent A = water, eluent B = 1 M aq. NH_4HCO_3, gradient from 0 to 30% B in 20 min, flow rate = 10 mL/min) gave **8** as a colorless foam after lyophilization.

^{1}H-NMR (D_2O, 300 MHz): δ7.38 (m, 1H), 7.30(m, 1H), 6.15 (t, 1H, J=6.0), 4.54 (m, 1H), 4.01-4.09 (m, 3H), 2.41 (m, 1H), 2.34 (m, 1H). ^{31}P-NMR (D_2O, 120 MHz): δ -10.01 (d, 1P), -10.51 (d, 1P), -22.48 (t, 1P).

Organic synthesis of dK^{n}TP: 5-(2′-Deoxy-β-D-ribofuranosyl)-2,6-diamino-3-nitropyridine 5′-triphosphate. Schematic of the synthesis is shown in **Supplementary Fig. 4**.

2-Amino-6-chloro-5-iodo-3-nitropyridine (10). A mixture of 2-amino-6-chloro-3-nitropyridine **9** (5.7 g, 32.8 mmol), water (4.5 mL), concentrated H_2SO_4 (1.26 mL) and H_5IO_6 (1.59 g) was stirred for 15 min at 95 °C. Iodine (3.6 g) was added in portions. The reaction mixture was stirred for 1 h at 95 °C, cooled to room temperature, poured into sat. aqueous sodium thiosulfate solution and extracted with ethyl acetate. The organic layer was dried (Na_2SO_4), filtered and concentrated. The residue was purified by silica gel column chromatography (Hex: EtOAc=3: 2) to give **10** (8.7 g, 29.1 mmol, 88%) as an orange solid.

^{1}H NMR(DMSO-d$_6$, 300 MHz) δ8.62 (s, 1H), 8.26 (brs, 2H).

2-Amino-5-[3′-*O*-(tert-butyldiphenylsilyl)-β-D-glycero-pentofuran-3′-ulos-1′-yl]-6-chloro-3-nitropyridine (12). A solution of palladium acetate (187 mg, 0.83 mmol) and triphenyl arsine (509 mg, 1.66 mmol) in chloroform (30 mL) was stirred for 30 min at room temperature. This solution was added to the mixture of glycal **11** (3.25 g, 9.2 mmol), **10** (2.49 g, 8.3 mmol) and silver carbonate (4.59 g, 16.6 mmol) in chloroform (60 mL) at room temperature. The reaction mixture was refluxed overnight, cooled to room temperature and filtered through a celite pad, the filtrate was concentrated and the residue was purified by silica gel column chromatography (Hex: EtOAc=4: 1 to 7: 3) to give compound **12** (2.75 g, 5.23 mmol, 63%) as an orange foam.

^{1}H NMR(CDCl$_3$, 300 MHz) δ8.42 (s, 1H), 7.73-7.82 (m, 4H), 7.41-7.48 (m, 6H), 5.83 (m, 1H), 7.77 (m, 1H), 4.23 (s, 1H), 3.90 (m, 2H), 1.78 (t, 1H, J=6.0), 1.23 (t, 1H, J=6.9), 1.08 (s, 9H).

2-Amino-5-(2′-deoxy-β-D-ribofuranosyl)-6-chloro-3-nitropyridine (14). To a stirred solution of **12** (2.75 g, 5.23 mmol) in THF (60 mL) was added AcOH (1.5 mL), followed by addition of 1 M TBAF in THF (7.9 mL) at 0 °C. After 30 min stirring, the reaction mixture was concentrated to give crude compound **13**, which was dissolved in CH_3CN/AcOH (46 mL/23 mL). To this mixture was added $Na(OAc)_3BH$ (1.66 g, 7.83 mmol) at 0 °C. After 1 h stirring at 0 °C, acetone was added and the reaction mixture was concentrated. The residue was purified by silica gel column chromatography (CH_2Cl_2: MeOH=15: 1) to give **14** (1.21 g, 4.18 mmol, 80%) as a yellow solid.

^{1}H NMR(DMSO-d$_6$, 300 MHz) δ8.49 (s, 1H), 8.14 (brs, 2H), 5.13 (d, 1H, J=3.9), 5.06 (dd, 1H, J=5.7, 9.9), 4.83 (t, 1H, J=5.4), 4.17 (m, 1H), 3.78 (m, 1H), 3.43-3.52 (m, 2H), 2.16 (dd, 1H, J = 5.7, 12.6), 1.66 (m, 1H).

5-(2′-Deoxy-β-D-ribofuranosyl)-2,6-diamino-3-nitropyridine (15). **14** (1.2 g, 4.14 mmol) was dissolved in 7 N NH_3 in MeOH (80 mL) and heated overnight at 110 °C. The reaction mixture cooled and concentrated. The residue was washed with ethanol/ether mixture to give **15** (1 g, 3.7 mmol, 90%) as a yellow solid.

^{1}H NMR(DMSO-d$_6$, 300 MHz) δ7.96 (s, 1H), 7.25 (brs, 4H), 5.01-5.15 (m, 2H), 4.88 (dd, 1H, J= 6.3, 9.6), 4.20 (m, 1H), 3.74 (m, 1H), 3.47-3.58 (m, 2H), 1.89-1.97 (m, 2H).
^{13}C NMR (DMSO-d$_6$, 75 MHz) δ160.6, 155.4, 133.7, 118.2, 112.7, 88.4, 78.1, 72.7, 62.1, 40.9.

5-[2′-Deoxy-5′-*O*-(4,4′-dimethoxytripenylmethyl)-β-D-ribofuranosyl]-2,6-diamino-3-nitropyridine (16). To a stirred solution of **15** (310 mg, 1.15 mmol) in pyridine (20 mL) was added DMTCl (428 mg, 1.26 mmol) at room temperature. After being stirred at room temperature for 3 h, catalytic amount of DMAP was added. The reaction mixture was stirred for an additional 1 h and concentrated. The residue was purified by silica gel column chromatography (Hex: EtOAc=1: 2 to 1: 4) to give **16** (410 mg, 0.72 mmol, 62%) as a yellow foam.

^{1}H-NMR(300 MHz, DMSO-d_6): δ 8.07 (s, 1H), 6.79-8.0 (m, 17H), 5.13 (d, 1H, J = 3.9), 4.94 (dd, 1H, J = 9.0, 6.0), 4.11 (m, 1H), 3.85 (m, 1H), 3.71 (s, 6H), 3.08 (d, 2H, J = 3.6), 2.15 (m, 1H), 1.86 (m, 1H).

5-[3′-*O*-Acetyl-2′-deoxy-5′-*O*-(4,4′-dimethoxytripenylmethyl)-β-D-ribofuranosyl]-2,6-diamino-3-nitropyridine (17). To a stirred solution of **16** (1.08 g, 1.89 mmol) in pyridine (40 mL) were added Ac_2O (0.25 mL, 2.63 mL) and catalytic amount of DMAP at room temperature. After being stirred at room temperature for 2 h, the reaction mixture was concentrated and the residue was purified by silica gel column chromatography (Hex: EtOAc=1: 2) to give **17** (1.08 g, 1.76 mmol, 93%) as a yellow foam.

^{1}H-NMR(300 MHz, $CDCl_3$): δ 8.09 (s, 1H), 7.21-7.38 (m, 9H), 6.82 (d, 4H, J = 7.8), 5.53 (d, 1H, J = 4.8), 4.93 (dd, 1H, J = 4.8, 11.4), 4.06 (m, 1H), 3.57 (dd, 1H, J = 1.8, 10.5), 3.38 (dd, 1H, J = 2.1, 10.2), 2.63 (m, 1H), 2.14 (m, 1H), 2.10(s, 3H).

5-(3′-*O*-Acetyl-2′-deoxy-β-D-ribofuranosyl)-2,6-diamino-3-nitropyridine (18). A mixture of **17** (1.08 g, 1.76 mmol) in 3% dichloroacetic acid in CH_2Cl_2 (40 mL) was stirred at room temperature for 1 h and concentrated. The residue was purified by silica gel column chromatography (EtOAc to EtOAc: MeOH=9: 1) to give **18** (512 mg, 1.64 mmol, 93%) as a yellow solid.

^{1}H-NMR(300 MHz, DMSO-d_6): δ 8.03(s, 1H), 7.90, 7.56 (2brs, 2H), 7.23 (brs, 2H), 5.33 (brs, 1H), 5.17 (m, 1H), 4.87 (t, 1H, J = 8.1), 3.93 (m, 1H), 3.65 (m, 1H), 3.51 (dd, 1H, J = 2.7, 11.4), 2.10 (m, 2H), 2.04 (s, 3H).

5-(2′-Deoxy-β-D-ribofuranosyl)-2,6-diamino-3-nitropyridine 5′-triphosphate (19). **18** (312 mg, 1 mmol) was dissolved in pyridine (6 mL) and dioxane (5 mL). To this mixture was added a solution of 2-chloro-1,3,2-benzodioxaphosphorin-4-one (300 mg, 1.48 mmol) in dioxane (3 mL) at room temperature. After 20 min stirring, a mixture of 0.2 M tributylammonium pyrophosphate in DMF (15 mL) and Bu_3N (1.6 mL) was added. After additional 20 min stirring, a mixture of I_2 (360 mg) and water (0.5 mL) in pyridine (25 mL) was added. After 30 min, the reaction mixture was quenched with 5% sodium sulfite solution. After solvents were removed, the residue was dissolved in water (30 mL) and left to stand at room temperature overnight. Water was removed and 25 % NH_4OH (50 mL) was added. The mixture was stirred at room temperature for 4 h and concentrated. The residue was dissolved in water (50 mL), and the mixture was filtered (0.2 μm). Purification by reverse phase HPLC (Sunfire Prep C_{18} column, 5 μm, 30 x 250 mm, eluent A = 25 mM TEAA pH 7, eluent B = CH_3CN in A, gradient from 0 to 40% B in 20 min, flow rate = 15 mL/min, Rt = 14 min), followed by ion-exchange HPLC (Dionex BioLC DNAPac PA-100, 22 x 250 mm, eluent A = water, eluent B = 1 M aq. NH_4HCO_3, gradient from 0 to 30% B in 20 min, flow rate = 10 mL/min, Rt = 15 min) gave compound **6** (180 μmol, 22%) as a yellow foam after lyophilization.

^{1}H-NMR (D_2O, 300 MHz): δ 7.96 (s, 1H), 4.86 (dd, 1H, J = 4.8, 11.1), 4.40 (m, 1H), 4.00 (m, 1H), 2.14 (m, 1H), 1.87(m, 1H); ^{31}P-NMR (D_2O, 120 MHz): δ -7.5 (d, 1P), -12.4 (d, 1P), -21.0 (t, 1P).

Organic synthesis of dJTP: 4-Amino-8-(2′-deoxy-β-D-erythro-pentofuranosyl)imidazo[1,2-a]-1,3,5-triazin-2-one 5′-triphosphate. Schematic of the synthesis is shown in **Supplementary Fig. 5**.

1-(2′-Deoxy-β-D-erythro-pentofuranosyl)-2-nitroimidazole (23). To a stirred suspension of 2-nitroimidazole **20** (2 g, 17.8 mmol), K_2CO_3 (8 g, 58 mmol) in CH_3CN (800 mL) was added TDA-1 (0.4 mL, 0.84 mmol) at room temperature. This mixture was stirred at RT for 1 h and chloro sugar **21** (8 g, 20.7 mmol) was added at room temperature. After stirring at room temperature for 2 h, the reaction mixture was filtered and the filtrate was evaporated. The residue was purified by silica gel column chromatography (Hex: EtOAc=2: 1) to give **22** as a white foam. To a solution of crude **22** in MeOH (150 mL) was added 40% $MeNH_2$ in water (10 mL) at RT. The reaction mixture was stirred overnight at RT and evaporated, then ethyl ether was added to the residue. The resulting precipitate was filtered to give **23** (3.4 g, 14.8 mmol, 84%) as a white solid.

^{1}H NMR (300 MHz, DMSO-d_6): δ 8.00 (s, 1H), 7.18 (s, 1H), 6.52 (t, 1H, J = 5.4 Hz), 5.29 (d, 1H, J = 4.5 Hz), 5.08 (t, 1H, J = 5.1 Hz), 4.23 (m, 1H), 3.83 (m, 1H), 3.55-3.66 (m, 2H), 2.42 (m, 1H), 2.28 (m, 1H). ^{13}C NMR (75 MHz, DMSO-d_6): δ 144.8, 128.4, 123.9, 89.2, 88.7, 69.4, 61.0, 42.6.

1-[2′-Deoxy-3′,5′-*O*-di-(*tert*-butyldimethylsilyl)-β-D-erythro-pentofuranosyl]-2-nitroimidazole (24). To a stirred solution of **23** (3.4 g, 14.8 mmol) in DMF (140 mL) were added imidazole (3 g, 44.1 mmol) and TBDMSCl (6.7 g, 44.5 mmol) at room temperature. The reaction mixture was stirred overnight at room temperature, poured into water (300 mL) and extracted with ethyl ether. The organic layer was dried (Na_2SO_4), filtered and evaporated. The residue was purified by silica gel column chromatography (Hex: EtOAc=4: 1) to give **24** (6.2 g, 13.5 mmol, 91%) as a white solid.

^{1}H NMR (300 MHz, $CDCl_3$): δ 7.91 (s, 1H), 7. 01 (s, 1H), 6.62 (dd, 1H, J = 4.2, 6.3 Hz), 4.45 (m, 1H), 3.77-3.97 (m, 3H), 2.59 (m, 1H), 2.19 (m, 1H), 0.91, 0.87 (2s, 18H), 0.11, 0.10, 0.05 (3s, 12H). ^{13}C NMR (75 MHz, $CDCl_3$): δ 128.4, 122.9, 89.1, 88.2, 69.6, 61.7, 43.7, 26.1, 25.9, 18.5, 18.1.

2-Amino-1-[2′-Deoxy-3′,5′-*O*-di-(*tert*-butyldimethylsilyl)-β-D-erythro-pentofuranosyl]-imidazole (25). A suspension of **24** (3.1 g, 6.8 mmol) and 10% Pd/C (800 mg) in EtOH (80 mL) was degassed. The reaction mixture was stirred overnight at room temperature under H_2, filtered through a celite pad and washed with MeOH. The filtrate was evaporated to give **25** (2.45 g, 5.7 mmol, 85%) as a pale yellow solid, which was used for the cyclization without further purification.

^{1}H NMR (300 MHz, DMSO-d_6): δ 6.68 (s, 1H), 6.38 (s, 1H), 5.84 (t, 1H, J = 6.6 Hz), 5.49 (brs, 2H), 4.39 (m, 1H), 3.73 (m, 1H), 3.62 (m, 2H), 2.23 (m, 1H), 2.05 (m, 1H), 0.87, .086 (2s, 18H), 0.08, 0.04, 0.03 (3s, 12H). ^{13}C NMR (75 MHz, $CDCl_3$): δ 149.7, 124.8, 111.4, 87.1, 83.6, 72.8, 63.5, 26.5, 26.4, 18.7, 18.5.

8-[2′-Deoxy-3′,5′-*O*-di-(*tert*-butyldimethylsilyl)-β-D-erythro-pentofuranosyl]imidazo[1,2-a]-1,3,5-triazin-2-one-4-thione (26). A mixture of phenyl chloroformate (0.17 mL) and potassium thiocyanate (150 mg) in EtOAc (5 mL) was stirred for 1 h at room temperature. To this mixture was added a solution of **25** (428 mg, 1 mmol) in 1,4-dioxane (4.5 mL) at room temperature. The reaction mixture was stirred for 4 h at 40 °C and MeOH (0.5 mL) was added. The mixture was evaporated and the residue was

purified by silica gel column chromatography (CH_2Cl_2: acetone=10: 1) to give **26** (165 mg, 0.32 mmol, 32%) as a yellow foam.

^{1}H NMR (300 MHz, $CDCl_3$): δ 9.65 (s, 1H), 7.51 (d, 1H, J = 3.0 Hz), 7.43 (d, 1H, J =2.7 Hz), 6.28 (t, 1H, J = 5.7 Hz), 4.45 (m, 1H), 3.88-3.96 (m, 2H), 3.75 (m, 1H), 2.35 (m, 1H), 2.21 (m, 1H), 0,93, 0.89 (2s, 18H), 0.12, 0.8 (2s, 12H). ^{13}C NMR (75 MHz, $CDCl_3$): δ 171.1, 153.3, 147.4, 116.4, 110.5, 88.2, 84.8, 71.2, 62.7, 42.0, 26.2, 25.9, 18.6, 18.2.

8-[2′-Deoxy-3′,5′-*O*-di-(*tert*-butyldimethylsilyl)-β-D-erythro-pentofuranosyl]-4-methylthioimidazo[1,2-a]-1,3,5-triazin-2-one-4-thione (27). Methyl iodide (0.32 mL, 5.1 mmol) was added to a mixture of **26** (870 mg, 1.7 mmol) and $NaHCO_3$ (214 mg, 2.04 mmol) in 1,4-dioxane (4 mL) and MeOH (8 mL). After being stirred for 30 h at room temperature, the reaction mixture was evaporated and the residue was purified by silica gel column chromatography (CH_2Cl_2: acetone=7: 3) to give **27** (600 mg, 1.14 mmol, 67%) as a white foam.

^{1}H NMR (300 MHz, $CDCl_3$): δ 7.34 (d, 1H, J = 2.7 Hz), 6.87 (d, 1H, J = 2.7 Hz), 6.37 (t, 1H, J = 6.0 Hz), 4.43 (m, 1H), 3.70-3.94 (m, 3H), 2.71 (s, 3H), 2.34 (m, 1H), 2.16 (m, 1H), 0.92, 0.88 (2s, 18H), 0.10, 0.06 (2s, 12H). ^{13}C NMR (75 MHz, $CDCl_3$): δ 160.2, 158.6, 149.1, 116.1, 106.6, 88.0, 84.2, 71.4, 62.8, 42.0, 26.2, 25.9, 18.7, 18.2, 13.3.

4-Amino-8-[2′-deoxy-3′,5′-*O*-di-(*tert*-butyldimethylsilyl)-β-D-erythro-pentofuranosyl]-imidazo[1,2-a]-1,3,5-triazin-2-one (28). A solution of **27** (600 mg, 1.14 mmol) in methanolic ammonia (7 N, 20 mL) was stirred for 40 h at room temperature, evaporated and the residue was purified by silica gel column chromatography (CH_2Cl_2: MeOH=9: 1) to give **28** (360 mg, 0.73 mmol, 64%).

^{1}H NMR (300 MHz, DMSO-d_6): δ 7.50-7.66 (m, 3H), 7.35 (d, 1H, J = 2.4 Hz), 6.05 (t, 1H, J = 6.6 Hz), 4.43 (m, 1H), 3.59-3.77 (m, 3H), 2.40 (m, 1H), 2.14 (m, 1H), 0.86, 0.85 (2s, 18H), 0.08, 0.04 (2s, 12H). ^{13}C NMR (75 MHz, DMSO-d_6): δ 155.5, 152.9, 150.7, 115.3, 107.8, 87.4, 83.0, 72.5, 63.2, 39.0, 26.4, 26.3, 18.6, 18.3.

8-[2′-Deoxy-3′,5′-*O*-di-(*tert*-butyldimethylsilyl)-β-D-erythro-pentofuranosyl]-4-[(2-methylpropionyl)amino]-imidazo[1,2-*a*]-1,3,5-triazin-2-one (29). To a stirred solution of **28** (800 mg, 1.61 mmol) and DMAP (100 mg) in pyridine (20 mL) was added isobutyryl chloride (0.254 mL, 2.42 mmol) at RT. After being stirred for 1 h at room temperature, the reaction mixture was evaporated and the residue was purified by silica gel column chromatography (Hex: EtOAc=1: 1) to give **29** (780 mg, 1.38 mmol, 85%) as a white foam.

^{1}H NMR (300 MHz, $CDCl_3$): δ 11.7 (brs, 1H), 7.37 (d, 1H, J = 2.7 Hz), 7.28 (d, 1H, J = 2.4 Hz), 6.30 (t, 1H, J = 6.0 Hz), 4.44 (m, 1H), 3.74-3.93 (m, 3H), 2.60 (m, 1H), 2.32 (m, 1H), 2.16 (m, 1H), 1.18, 1.16 (2s, 6H), 0.92, 0.87 (2s, 18H), 0.10, .006 (2s, 12H). ^{13}C NMR (75 MHz, $CDCl_3$): δ 192.5, 152.5, 149.0, 147.9, 115.9, 107.9, 88.2, 84.4, 71.4, 62.8, 42.0, 39.8, 26.2, 25.9, 19.4, 18.6, 18.2.

8-(2′-Deoxy-β-D-erythro-pentofuranosyl)-4-(2-methylpropionyl)amino-imidazo[1,2-*a*]-1,3,5-triazin-2-one (30). To a stirred solution of **29** (780 mg, 1.38 mmol) in THF (20 mL) was added a solution

of HF (70% in pyridine, 0.95 mL) in pyridine (1.2 mL) at 0 °C. After being stirred overnight at room temperature, the reaction mixture was evaporated and the residue was purified by silica gel column chromatography (CH_2Cl_2: MeOH=8: 1) to give **30** (390 mg, 1.16 mmol, 84%) as a white solid.

^{1}H NMR (300 MHz, DMSO-d_6): δ 11.5 (s, 1H), 7.55 (d, 1H, J = 2.7Hz), 7.48 (d, 1H, J = 2.7 Hz), 6.07 (t, 1H, J = 6.6 Hz), 5.29 (d, 1H, J = 4.2 Hz), 4.99 (t, 1H, J = 5.4 Hz), 4.28 (m, 1H), 3.79 (m, 1H), 3.48-3.58 (m, 2H), 2.52 (m, 1H), 2.27 (m, 1H), 2.13 (m, 1H), 1.10, 1.08 (2s, 6H). ^{13}C NMR (75 MHz, DMSO-d_6): δ 191.6, 152.3, 149.9, 148.0, 117.2, 108.9, 88.4, 83.9, 70.9, 61.9, 41.0, 39.3, 19.7.

8-[2′-Deoxy-5′-*O*-(4,4′-dimethoxytripenylmethyl)-β-D-erythro-pentofuranosyl]-4-[(2-methylpropionyl)amino]-imidazo[1,2-*a*]-1,3,5-triazin-2-one (31). To a stirred suspension of **30** (380 mg, 1.13 mmol) and DMT-Cl (460 mg, 1.36 mmol) in CH_2Cl_2 (15 mL) was added Et_3N (0.32 mL, 2.26 mmol) at room temperature. After being stirred overnight at room temperature, the reaction mixture was evaporated and the residue was purified by silica gel column chromatography (EtOAc to EtOAc: MeOH=9: 1) to give **31** (620 mg, 0.97 mmol, 86%) as a pale yellow foam.

^{1}H NMR (300 MHz, $CDCl_3$): δ 11.73 (s, 1H), 6.81-7.41 (m, 11H), 6.33 (t, 1H, J = 6.0 Hz), 4.67 (m, 1H), 4.13 (m, 1H), 3.79 (s, 6H), 3.42-3.52 (m, 3H), 2.57-2.68 (m, 2H), 2.43 (m, 1H), 1.19, 1.16 (2s, 6H).^{13}C NMR (75 MHz, $CDCl_3$): δ 192.6, 158.9, 152.8, 148.9, 147.7, 144.5, 135.6, 135.5, 130.4, 128.5, 128.2, 127.3, 116.3, 113.5, 108.1, 87.1, 86.5, 84.5, 71.6, 55.5, 41.5, 39.8, 19.4.

8-[3′-*O*-Acetyl-2′-deoxy-5′-*O*-(4,4′-dimethoxytripenylmethyl)-β-D-erythro-pentofuranosyl]-4-[(2-methylpropionyl)amino]-imidazo[1,2-*a*]-1,3,5-triazin-2-one (32). To a stirred solution of **31** (420 mg, 0.66 mmol) in pyridine (10 mL) was added Ac_2O (0.093 mL, 0.98 mmol) at room temperature. The reaction mixture was stirred overnight at room temperature and evaporated. The residue was purified by silica gel column chromatography (EtOAc) to give **32** (440 mg, 0.65 mmol, 98%) as a pale yellow foam.

^{1}H NMR (300 MHz, $CDCl_3$): δ 11.72 (brs, 1H), 7.25-7.39 (m, 9H), 7.16 (d, 1H, J = 2.7 Hz), 7.02 (d, 1H, J = 2.7 Hz), 6.82 (d, 4H, J = 8.7 Hz), 6.38 (t, 1H, J = 7.2 Hz), 5.43 (m, 1H), 4.19 (m, 1H)m 3.79 (s, 6H), 3.46 (m, 2H), 2.63 (m, 1H), 2.51(m, 2H), 2.09 (s, 3H), 1.19, 1.17 (2s, 6H). ^{13}C NMR (75 MHz, $CDCl_3$): δ 192.5, 170.6, 158.9, 152.4., 149.6, 147.8, 144.4, 135.4, 135.3, 130.4, 128.4, 128.3, 127.4, 115.5, 113.5, 108.5, 87.3, 84.6, 83.9, 75.1, 63.7, 55.5, 39.9, 38.6, 21.2, 19.4.

8-(3′-*O*-Acetyl-2′-deoxy-β-D-erythro-pentofuranosyl)-4-[(2-methylpropionyl)amino]-imidazo[1,2-*a*]-1,3,5-triazin-2-one (33). A solution of **32** (440 mg, 0.65 mmol) in 3% trichloroacetic acid in CH_2Cl_2 (20 mL) was stirred for 2 h at room temperature and evaporated. The residue was purified by silica gel column chromatography (CH_2Cl_2: MeOH=10: 1) to give **33** (200 mg, 0.53 mmol, 82%) as a white solid.

^{1}H NMR (300 MHz, DMSO-d_6): δ 9.19 (s, 1H), 7.79 (d, 1H, J = 3.6 Hz), 7.13 (t, 1H, J =2.7 Hz), 6.74 (t, 1H, J =2.7 Hz), 5.97 (m, 1H) 5.22 (m, 1H), 3.96 (m, 1H), 3.533.54 (m, 2H), 2.37-2.53 (m, 2H), 2.22 (m, 1H), 2.06 (s, 3H), 0.85 (m, 6H). ^{13}C NMR (75 MHz, DMSO-d_6): δ 195.1, 176.2, 170.7, 157.7, 151.1, 114.1, 97.3, 85.5, 83.5, 75.7, 62.2, 36.9, 34.1, 21.6, 19.9.

4-Amino-8-(2′-deoxy-β-D-erythro-pentofuranosyl)imidazo[1,2-a]-1,3,5-triazin-2-one 5′-triphosphate (34). To a solution of **33** (260 mg, 0.69 mmol) in pyridine (6 mL) and dioxane (4.5 mL) was added a solution of 2-chloro-4H-1,3,2-benzodioxaphosphorin-4-one (214 mg, 1.06 mmol) in dioxane (2.1 mL) at RT. After 15 min a mixture of tributylammonium pyrophosphate in DMF (0.2 M, 10.5 mL, 2.1 mmol) and tributylamine (1.14 mL, 4.8 mmol) was added. After 20 min a solution of iodine (255 mg, 1.0 mmol) and water (0.35 mL) in pyridine (18 mL) was added. After 20 min the reaction was quenched by the addition of aqueous Na_2SO_3 (5%, 1 mL). The solvents were removed *in vacuo*. NH_4OH (30 mL) was added, and the mixture was stirred overnight at room temperature. After evaporation, the residue was dissolved in water (50 mL) and filtered (0.2 μm). Purification by ion-exchange HPLC (Dionex BioLC DNAPac PA-100, 22 x 250 mm, eluent A = water, eluent B = 1 M aq. NH_4HCO_3, gradient from 0 to 40% B in 20 min, flow rate = 10 mL/min, Rt = 12 min), followed by reverse phase HPLC (SunFires Prep C18 column, 5 μm, 19 x 250 mm, eluent A = 25 mM TEAA pH 7, eluent B = CH_3CN, gradient from 0 to 20% B in 20 min, flow rate = 10 mL/min, Rt = 10 min) gave **34** as a colorless foam after lyophilization.

^{1}H NMR (300 MHz, D_2O): δ 7.44 (m, 1H), 7.34 (m, 1H), 6.19 (t, 1H, J = 6.6 Hz), 4.56 (m, 1H), 4.01- 4.07 (m, 3H), 2.38 (m, 1H), 2.32 (m, 1H). ^{31}P NMR (120 MHz, D_2O): δ -9.78 (br, 1p), -10.42 (br, 1P), -22.13 (br, 1P).

Organic synthesis of dVTP: 2-Amino-5-nitro-3-(2′-deoxy-β-D-ribofuranosyl)-1*H*-pyridin-6-one-5′-triphosphate. Schematic of the synthesis is shown in **Supplementary Fig. 6**.

2-Amino-5-nitro-3-(2′-deoxy-β-D-ribofuranosyl)-6-[2-(4-nitrophenyl)ethoxy]-pyridine (36). Palladium acetate (0.055 g, 0.243 mmol) and triphenylarsine (0.175 g, 0.486 mmol) were dissolved in chloroform (10 mL), and the mixture was stirred at rt for 30 min. Then it was added to a mixture of compound **35** (1.05 g, 2.43 mmol), glycal (0.86 g, 2.43 mmol) and silver carbonate (1.34 g, 4.86 mmol) in chloroform (20 mL). The resulting mixture was refluxed overnight. After cooling to rt, it was filtered through Celite and washed with ethyl acetate. The combined filtrate was concentrated *in vacuo*. The residue was purified by flash chromatography (silica, hexanes: EtOAc=2: 1 to 1: 1) to give a brown solid. This solid was dissolved in THF (20 mL) and treated with HF-pyridine (0.58 mL) and stirred at rt for 1 h. The mixture was evaporated with silica gel and the residue was purified by flash chromatography (silica, hexanes: EtOAc=1: 2 to 100% EtOAc) to give a yellow solid. This material, without further characterization, was dissolved in acetic acid (10 mL) and acetonitrile (10 mL) and treated with sodium triacetoxyborohydride (0.602 g, 2.84 mmol) and stirred at rt for 1 h. The mixture was poured into brine and extracted with EtOAc, and the organic layer was dried over Na_2SO_4. Solvent was removed under reduced pressure, the residue was purified by flash chromatography (silica, EtOAc to EtOAc: MeOH=30: 1) to give compound **36** as a yellow solid (0.59 g, 59% for 3 steps).

^{1}H NMR (300 MHz, DMSO-d_6) δ 8.18 (s, 1H), 8.15 (d, J = 2.1 Hz, 1H), 7.66 (d, J = 8.4 Hz, 2H), 7.49 (s, 2H), 5.16 (t, J = 4.7 Hz, 1H), 5.09 (d, J = 3.9 Hz, 1H), 5.01 (t, J = 8.0 Hz, 1H), 4.58 (t, J = 6.5 Hz, 2H), 4.24 (m, 1H), 3.80 (d, J = 2.4 Hz, 1H), 3.50-3.62 (m, 2H), 3.21 (t, J = 6.5 Hz, 2H), 1.97-1.99 (m, 2H). ^{13}C NMR (75 MHz, DMSO-d_6) δ 158.6, 156.6, 146.8, 146.3, 135.0, 130.5, 123.3, 120.9, 112.2, 87.8, 76.7, 72.1, 66.4, 61.4, 34.4.

2-{[(dimethylamino)methylidene]amino}-5-nitro-3-(2′-deoxy-β-D-ribofuranosyl)-6-[2-(4-nitrophenyl)ethoxy]-pyridine (37). A mixture of **36** (1.48 g, 3.52 mmol) and *N,N*-dimethylformamide dimethyl acetal (1.87 mL, 14.08 mmol) in methanol (20 mL) was stirred at rt overnight. The mixture was evaporated and purified by flash chromatography (neutral silica, EtOAc: MeOH=30: 1 to 10: 1) to give compound **37** as a yellow solid (1.47 g, 88%).

^{1}H NMR (300 MHz, DMSO-d_6) δ 8.65 (s, 1H), 8.31 (s, 1H), 8.16 (d, J = 8.7 Hz, 2H), 7.64 (d, J = 8.4 Hz, 2H), 5.25 (dd, J = 9.5, 5.6 Hz, 1H), 5.03 (d, J = 3.6 Hz, 1H), 4.80 (J = 5.3 Hz, 1H), 4.70 (m, 2H), 4.12 (s, 1H), 3.76-3.77 (m, 1H), 3.42-3.55 (m, 2H), 3.21 (s, 5H), 3.10 (s, 3H), 2.31 (dd, J = 12.5, 5.3 Hz, 1H), 1.52-1.62 (m, 1H). ^{13}C NMR (75 MHz, DMSO-d_6) δ 159.9, 157.1, 154.7, 147.1, 146.2, 133.1, 130.5, 125.6, 124.0, 123.3, 87.2, 74.3, 72.1, 66.3, 62.3, 42.1, 40.8, 34.8, 34.5.

2-{[(dimethylamino)methylidene]amino}-5-nitro-3-[2′-deoxy-5′-*O*-(4,4′-dimethoxytrityl)-β-D-ribofuranosyl]-6-[2-(4-nitrophenyl)ethoxy]-pyridine (38). To a mixture of **37** (1.15 g, 3.05 mmol), TEA (1.39 mL, 13.7 mmol) and DMAP (37 mg, 0.305 mmol) in CH_2Cl_2 (40 mL) was added DMTr-Cl (3.02 g, 7.78 mmol) and the mixture was stirred at rt overnight. This was poured into water and extracted with CH_2Cl_2 and the organic layer was dried over Na_2SO_4. Solvent was removed under reduced pressure; the residue was purified by chromatography (neutral silica, EtOAc: hexanes=3: 1 to EtOAc) to give compound **38** as dark-yellow solid. (3.16 g, 91%).

^1H NMR (300 MHz, $CDCl_3$) δ 8.48 (s, 1H), 8.43 (s, 1H), 8.16 (d, J = 8.4 Hz, 2H), 7.54 (d, J = 8.7 Hz, 2H), 7.45 (d, J = 7.5 Hz, 2H), 7.26-7.36 (m, 6H), 7.17-7.22 (m, 1H), 6.84 (d, J = 8.1 Hz, 4H), 5.41 (dd, J = 9.7, 6.2 Hz, 1H), 4.62 (t, J = 6.0 Hz, 2H), 4.31-4.32 (m, 1H), 4.02 (dd, J = 8.4, 4.8 Hz, 1H), 3.79 (s, 6H), 3.43 (dd, J = 9.6, 4.8 Hz, 1H), 3.21-3.27 (m, 3H), 3.16 (s, 3H), 3.12 (s, 3H), 2.46 (ddd, J = 13.2, 6.0, 2.4 Hz, 1H), 1.78-1.90 (m, 2H).^{13}C NMR (75 MHz, $CDCl_3$) δ 159.6, 158.5, 156.0, 155.0, 146.8, 146.4, 144.8, 135.9, 135.8, 133.8, 130.2, 130.0, 128.1, 127.8, 127.2, 126.8, 123.8, 123.6, 113.1, 86.3, 85.5, 75.1, 74.7, 66.5, 64.5, 60.4, 55.2, 42.1, 41.1, 35.3, 35.2, 21.0, 14.2.

2-{[(dimethylamino)methylidene]amino}-5-nitro-3-[2′-deoxy-5′-*O*-(4,4′-dimethoxytrityl)-3′-*O*-acetyl-β-D-ribofuranosyl]-6-[2-(4-nitrophenyl)ethoxy]-pyridine (39). To a solution of **38** (0.3 g, 0.39 mmol), DMAP (4.8 mg, 0.039 mmol) and pyridine (0.125 mL, 1.56 mmol) in CH_2Cl_2 (5 mL) was added Ac_2O (0.074 mL) and the mixture was stirred at rt overnight. The reaction was quenched with brine, extracted with CH_2Cl_2 and the organic layer was dried over Na_2SO_4. Solvent was removed under reduced pressure, the residue was purified by chromatography (neutral silica, hexanes: EtOAc=1: 1 to 1: 4) to give compound **39** as a yellow solid product. (0.282 g, 88%).

^1H NMR (300 MHz, $CDCl_3$) δ 8.51 (s, 1H), 8.48 (s, 1H), 8.17 (d, J = 8.4 Hz, 2H), 7.55 (d, J = 8.4 Hz, 2H), 7.44 (d, J = 7.5 Hz, 2H), 7.35 (d, J = 8.7 Hz, 4H), 7.30-7.26 (m, 2H), 7.16-7.21 (m, 1H), 6.84 (d, J = 8.4 Hz, 4H), 5.30-5.36 (m, 1H), 5.26 (d, J = 5.4 Hz, 1H), 4.63 (t, J = 6.2 Hz, 2H), 4.17-4.18 (m, 1H), 3.79 (m, 6H), 3.28-3.39 (m, 2H), 3.24 (t, J = 6.2 Hz, 2H), 3.18 (s, 3H), 3.11 (s, 3H), 2.58 (dd, J = 13.8, 5.1 Hz, 1H), 2.07 (s, 3H), 1.85-1.95 (m, 1H).^{13}C NMR (75 MHz, $CDCl_3$) δ 170.5, 159.6, 158.4, 156.0, 155.1, 146.8, 146.4, 144.8, 135.9, 135.8, 133.9, 130.2, 130.1, 130.0, 128.1, 127.8, 127.3, 126.7, 123.6, 123.1, 113.1, 107.2, 86.2, 83.5, 75.5, 66.5, 64.0, 55.2, 41.2, 40.0, 35.3, 35.0, 21.2.

2-{[(dimethylamino)methylidene]amino}-5-nitro-3-(2′-deoxy-3′-*O*-acetyl-β-D-ribofuranosyl)-6-[2-(4-nitrophenyl)ethoxy]-pyridine (40). To a solution of **39** (0.232 g, 0.28 mmol) in CH_2Cl_2 was added $Cl_2CHCOOH$ (0.23 mL, 2.08 mL) and the mixture was stirred at rt for 1 h. The reaction was quenched with saturated $NaHCO_3$ solution, extracted with CH_2Cl_2 and the organic layer was dried over Na_2SO_4. Solvent was removed under reduced pressure, the residue was purified by chromatography (neutral silica, hexanes: EtAOc=1: 3 to 100% EtOAC) to give compound **40** as a yellow solid. (0.122 g, 85%).

^1H NMR (300 MHz, $CDCl_3$) δ 8.50 (s, 1H), 8.39 (s, 1H), 8.16 (d, J = 8.7 Hz, 2H), 7.54 (d, J = 8.4 Hz, 2H), 5.29 (dd, J = 10.4, 5.3 Hz, 1H), 5.22 (d, J = 5.4 Hz, 1H), 4.63 (t, J = 6.0 Hz, 2H), 4.06 (d, J = 2.7 Hz, 1H), 3.85 (m, 2H), 3.20-3.25 (m, 5H), 3.15 (s, 3H), 2.44-2.50 (m, 2H), 2.06-2.16 (m, 4H).^{13}C NMR (75 MHz, $CDCl_3$) δ 170.9, 160.3, 156.6, 155.3, 146.8, 146.3, 134.9, 130.2, 126.9, 123.6, 121.5, 85.1, 76.9, 66.6, 63.2, 41.4, 39.2, 35.3, 35.3, 21.1.

2-Amino-5-nitro-3-(2′-deoxy-β-D-ribofuranosyl)-1*H*-pyridin-6-one-5′-triphosphate (41). To a solution of **6** (64.2 mg, 0.12 mmol) in pyridine (0.8 mL) and dioxane (2.4 mL) was added a solution of 2-chloro-4H-1,3,2-benzodioxaphosphorin-4-one (48 mg, 0.24 mmol) in dioxane (1.0 mL) at room temperature. After 15 min, a mixture of tributylammonium pyrophosphate in DMF (0.2 M, 2.4 mL, 0.48 mmol) and tributylamine (0.27 mL, 1.1 mmol) was added. After 20 min, a solution of iodine (61 mg, 0.24

mmol) and water (0.095 mL) in pyridine (4.76 mL) was added. After 30 min, the reaction was quenched by the addition of aqueous Na_2SO_3 (5%, until color disappears). The pyridine and dioxane were removed under reduced pressure. The residue was dissolved in acetonitrile (3 mL) and DBU (0.5 mL). The mixture was stirred at room temperature for 4 h. The volatiles were removed under reduced pressure and dissolved in ammonium hydroxide (10 mL). The mixture was stirred at room temperature overnight. Ammonia was removed by rotary evaporation, and the residue was diluted with water (20 mL). Purification by ion-exchange HPLC (water to water: 1 M ammonium bicarbonate = 50: 50 in 25 min) gave the triphosphate **41** as a yellow solid after lyophilization (ε = 11800 in H_2O, λ = 391 nm, 88.5 μmoles, 74%).

^{1}H NMR (300 MHz, D_2O) δ 8.37 (s, 1H), 5.06 (dd, *J* = 8.1, 5.1 Hz, 1H), 4.58 (d, *J* = 5.4 Hz, 1H), 4.15-4.20 (m, 3H), 2.31-2.42 (m, 1H), 2.05 (dd, *J* = 13.8, 5.1 Hz, 1H). ^{31}P NMR (121 MHz, D_2O) δ −8.4 (m, 1P), −10.4 (d, *J* = 18.3 Hz, 1P), −21.5 (m, 1P).

Supplementary Notes

Supplementary Note 1. Xenonucleotide substitution basecalling with Xenomorph. End-to-end pipeline for processing raw nanopore reads into xenonucleotide basecalls is available on the Xenomorph github repository (https://github.com/xenobiolab/xenomorph). The repository is also available on Zenodo (https://doi.org/10.5281/zenodo.8356450). The empirically measured 4-nt kmer models for all standard DNA bases (A, T, G, C) and all xenonucleobases (B, S^n, S^c, P, Z, X^t, K^n, J, V) are integrated for selection. The pipeline, as built, also allows users to generate their own models. Basecalling can be performed either per-read or per-sequence (global). In per-read basecalling, individual reads are basecalled while in per-sequence, the signal of all reads that match a sequence are averaged before determining a global call. The per-read consensus is defined as the most frequent basecall among all reads that match a certain sequence.

4-nt kmer models are parameterized with a kmer mean (μ_k) and a kmer variance (σ_k). Users have the choice of setting experimentally measured signal means, signal medians, or means from kernel density estimates as μ_k. Options for σ_k values are either the kmer-specific measured variance or a fixed global variance. The choice of bases to use in the model can also be specified. As described, basecalling in this work uses signal means for μ_k and global average kmer variance for σ_k.

Full code and documentation of Xenomorph is available on github. Sample data, such as the FAST5 data generated in this work, can be found in the SRA under Bioproject PRJNA932328 (**Supplementary Table 31**).

Supplementary Note 2. Per-read recall of simulated reads for artificial genetic codes. We explored the statistical limits of per-read recall of the 4-nt XNA/DNA kmer model on simulated reads for a few theoretical, but synthetically accessible, expanded genetic alphabets (**Supplementary Table 27-30**, **Supplementary Fig. 31 and 32**). For each genetic alphabet, we simulated 1,000 sets of signal levels for every possible heptamer sequence (4,096 possible sequences of the form NNN<u>N</u>NNN) and then basecalled these sequences using Xenomorph. We found that average per-read recall decreases as you increase the density of signal levels, from 88.5 ± 7.5% average recall for the standard genetic code to 65 ± 11% averaged recall with the 12-letter supernumerary (S = S^n) genetic code. Even in this most complex case, per-read recall is strongly sequence-specific with certain sequences showing > 80% recall while others < 30%. These simulations suggest an upper bound for single nucleotide recall and can be used to guide design and sequencing constraints.

Supplementary Results

Supplementary Table 1. Full description and abbreviations of XNA nucleobases used in this work. Base abbreviation (Base), base pair (BP), and full chemical name of XNA nucleosides (Nucleoside) used in this work. Additional references for each base and base pair are provided in the (Ref) column.

Base	BP	Nucleoside	Ref
B	S^n/S^c	6-amino-9-(1′-beta-D-2′-deoxyribofuranosyl)4-hydroxy-5-(hydroxymethyl)oxolan-2-yl]-1H-purin-2-one	8,47,48
S^n	B	2-amino-5-methyl-1-(1′-beta-D-2′-deoxyribofuranosyl)-4(1H)-pyrimidinone	47,48
S^c	B	3-methyl-6-amino-5-(1′-beta-D-2′-deoxyribofuranosyl)-pyrimidin-2-one	8
P	Z	2-amino-8-(β-D-2′-deoxyribofuranosyl)-imidazo-[1,2a]-1,3,5-triazin-[8H]-4-one	8
Z	P	6-amino-3-(2′-deoxy)-D-ribofuranosyl)-5-nitro-1H-pyridin-2-one	8
X^t	K^n	8-(β-D-2′-deoxyribofuranosyl)imidazo[1,2-a]-1,3,5-triazine-2(8H)-4(3H)-dione	49
K^n	X^t	5-nitro-2,4-diamino-5-(1′-beta-D-2′-deoxyribofuranosyl)-pyridine	50
J	V	4-amino-8-(1′-beta-D-2′-deoxyribofuranosyl)-imidazo[1,2-a]-1,3,5-triazin-2(8H)-one	51
V	J	2-amino-3-(2′-deoxy)-D-ribofuranosyl)-5-nitro-1H-pyridin-6-one	51

Supplementary Table 2. Synthetic hairpin sequences. Names of hairpins and sequences of hairpins used in screening and optimization of non-library XNA tailing and XNA ligation reactions. All sequences shown in 5′ to 3′ direction.

Hairpin name	Sequence
HP-3′PT	ATCTTGGCTCGCTAAAAGACCACGGGCCTCTTTTTGAGGCCCGTGGTCTTTTAGCGAGCC*A*A*G*A*T
5′Phos-15HP	/5Phos/ATCTTGACTCGCTAAAAGACCACGGGCCTCTTTTCCTTTGTTGTTGAGGCCCGTGGTCTTTTAGCGAGTCAAGAT
5′Phos-11HP	/5Phos/AACCCAGAAACGCTGGTGAAAGTAAAAGATGCTGATTTTCGTTGTTTCAGCATCTTTTACTTTCACCAGCGTTTCTGGGTT
5′Phos-HP-3′G	/5Phos/CACCCAGAAACGCTGGTGAAAGTAAAAGATGCTGATTTTCGTTGTTTCAGCATCTTTTACTTTCACCAGCGTTTCTGGGTGG
5′Phos-HP-3′C	/5Phos/ATCTTGACTCGCTAAAAGACCACGGGCCTCTTTTCCTTTGTTGTTGAGGCCCGTGGTCTTTTAGCGAGTCAAGATC
5′Phos-NdeI-HP-1	/5Phos/ATGTCAGCGTAAAAAAAAAAGTTCGATAGAAGTCTTCAGCTCTTTTTGAGCTGAAGACTTCTATCGAACTTTTTTTTTTACGCTGACAT
5′Phos-NdeI-HP-2	/5Phos/ATGGTGCCACCTGAGCCTTCAACCGCTATAAGTCTTCAGGCGTTTTTCGCCTGAAGACTTATAGCGGTTGAAGGCTCAGGTGGCACCAT
5′Phos-ScaI-HP	/5Phos/AAAGTACTATTCTGAAGTTCCTGGGTCTTGCCAAGTTTTTCTTGGCAAGACCCAGGAACTTCAGAATAGTACTTT

/5Phos/ = 5′-PO_4
*N = phosphorothioate bond

Supplementary Table 3. Synthetic hairpin NNN-oligo pools and hairpin 20mer-validation pools. Two sets of libraries were constructed in this work: 1) a random NNN-Pool library and 2) a validation library. NNN-Pool library was generated by ligating two hairpins together, each containing a randomized NNN-3′ end (library size = 64 x 64). The validation library was constructed by ligating hairpin pools that consist of a constant region followed by a randomly chosen 20mer sequence. Each hairpin pool contains 10 unique sequences. Ligating two hairpin pools together generates a final library of 100 possible sequence combinations (10 x 10). The table shows constant regions for all oligos in each pool (black), with regions in brackets (blue, bold) being replaced with their corresponding sequence elements from **Supplementary Table 4-6**. '-F' and '-R' are used to note forward and reverse sequences of different components after the hairpin is folded. NNN denotes the 3 randomized bases at the end of the hairpins, while [NNN-BC] (i.e., Triplet-barcode) and [Pool-BC] (i.e., Pool-barcode) are the barcodes that link to the 3′-NNN randomized bases and the tailed XNA, respectively. Regions highlighted in red denote restriction site sequence difference between HP_v1 and HP_v2, HP1 and HP2. All sequences are shown in the 5′ to 3′ direction. Full hairpin sequences purchased for this work can be found in **Supplementary Data 2**.

NNN Pool	Sequence Construction	n
HP_v1-NNN-[Pool-ID]	/5Phos/**[NNN-R]**TCAGCAGT**[NNN-BC-R]**GATC**[Pool-BC-R]**ATCGAAAAAAATTAGTACT**ATAGAA**GTCTTCTAGCTCTTTTCGTTGTTGAGCTAGAAGAC**TTCTAT**AGTACTAATTTTTTTCGAT**[NNN-BC-F]**GATC**[Pool-BC-F]**ACTGCTGA**[NNN-F]**	64
HP_v2-NNN-[Pool-ID]	/5Phos/**[NNN-R]**TCAGCAGT**[NNN-BC-R]**GATC**[Pool-BC-R]**ATCGAAAAAAATTAGTACT**CTATAA**GTCTTCTAGCTCTTTTCGTTGTTGAGCTAGAAGAC**TTATAG**AGTACTAATTTTTTTCGAT**[NNN-BC-F]**GATC**[Pool-BC-F]**ACTGCTGA**[NNN-F]**	64
**Validation Pool**	**Sequence Construction**	
HP1-[VAL-ID]	/5Phos/**[VAL-R]CTATAA**GTCTTCTAGCTCTTTTTGAGCTAGAAGAC**TTATAG[VAL-F]**	10
HP2-[VAL-ID]	/5Phos/**[VAL-R]ATAGAA**GTCTTCTAGCTCTTTTTGAGCTAGAAGAC**TTCTAT[VAL-F]**	10
	/5Phos/ = 5′-PO_4	

Supplementary Table 4. Library pool-barcodes sequences. Sequences of pool barcodes used for construction of NNN-libraries. Pool barcodes were used to identify which XNA was tailed onto the 3′-end of each hairpin pool from sequencing results (shown in **Supplementary Table 12**). Pool barcode sequences are used to construct the HP_v1-NNN-[Pool-ID] and HP_v2-NNN-[Pool-ID] hairpin sequences shown in **Supplementary Table 3** by insertion into the [Pool-BC] region. Sequences shown in 5′ to 3′ direction.

[Pool-ID]	[Pool-BC]	Pool Name
P1	AAGGTTCC	HP_v1-NNN-P1
P2	CTTACTCG	HP_v2-NNN-P2
P3	AGTCAGCT	HP_v1-NNN-P3
P4	TCCTGGAA	HP_v2-NNN-P4

Supplementary Table 5. Triplet-barcodes sequences. Sequences of the Triplet-barcodes and NNN sequences they are assigned to. The Triplet-barcode is a 24 nt sequence that is distal to the 3′-NNN end in each hairpin and is used to assign the true identity of the 3′-NNN bases that flank XNA insertions (**Fig. 3a**). Each NNN combination (N = A, T, G, or C; 64 NNN combinations) has a corresponding Triplet-barcode that maps to it 1:1. Barcode sequences were chosen from Oxford Nanopore Technologies list of barcodes for long-read sequencing. Barcode sequences are shown in 5′ to 3′ direction. The Triplet-barcode (abbreviated as [NNN-BC]) and NNN sequences used to construct HP_v1-NNN-[Pool-ID] and HP_v2-NNN-[Pool-ID] hairpin sequences, shown in **Supplementary Table 3**, by insertion into [NNN-BC] and [NNN] regions, respectively. Full sequences of all hairpins used for model generation can be found in **Supplementary Data 2**.

BC-ID	Triplet-barcode [NNN-BC]	[NNN]	BC-ID	Triplet-barcode [NNN-BC]	[NNN]
NB01	CACAAAGACACCGACAACTTTCTT	AAA	NB33	CAGACTTGGTACGGTTGGGTAACT	CAA
NB02	ACAGACGACTACAAACGGAATCGA	AAG	NB34	GGACGAAGAACTCAAGTCAAAGGC	CAG
NB03	CCTGGTAACTGGGACACAAGACTC	AAC	NB35	CTACTTACGAAGCTGAGGGACTGC	CAC
NB04	TAGGGAAACACGATAGAATCCGAA	AAT	NB36	ATGTCCCAGTTAGAGGAGGAAACA	CAT
NB05	AAGGTTACACAAACCCTGGACAAG	AGA	NB37	GCTTGCGATTGATGCTTAGTATCA	CGA
NB06	GACTACTTTCTGCCTTTGCGAGAA	AGG	NB38	ACCACAGGAGGACGATACAGAGAA	CGG
NB07	AAGGATTCATTCCCACGGTAACAC	AGC	NB39	CCACAGTGTCAACTAGAGCCTCTC	CGC
NB08	ACGTAACTTGGTTTGTTCCCTGAA	AGT	NB40	TAGTTTGGATGACCAAGGATAGCC	CGT
NB09	AACCAAGACTCGCTGTGCCTAGTT	ACA	NB41	GGAGTTCGTCCAGAGAAGTACACG	CCA
NB10	GAGAGGACAAAGGTTTCAACGCTT	ACG	NB42	CTACGTGTAAGGCATACCTGCCAG	CCG
NB11	TCCATTCCCTCCGATAGATGAAAC	ACC	NB43	CTTTCGTTGTTGACTCGACGGTAG	CCC
NB12	TCCGATTCTGCTTCTTTCTACCTG	ACT	NB44	AGTAGAAAGGGTTCCTTCCCACTC	CCT
NB13	AGAACGACTTCCATACTCGTGTGA	ATA	NB45	GATCCAACAGAGATGCCTTCAGTG	CTA
NB14	AACGAGTCTCTTGGGACCCATAGA	ATG	NB46	GCTGTGTTCCACTTCATTCTCCTG	CTG
NB15	AGGTCTACCTCGCTAACACCACTG	ATC	NB47	GTGCAACTTTCCCACAGGTAGTTC	CTC
NB16	CGTCAACTGACAGTGGTTCGTACT	ATT	NB48	CATCTGGAACGTGGTACACCTGTA	CTT
NB17	ACCCTCCAGGAAAGTACCTCTGAT	GAA	NB49	ACTGGTGCAGCTTTGAACATCTAG	TAA
NB18	CCAAACCCAACAACCTAGATAGGC	GAG	NB50	ATGGACTTTGGTAACTTCCTGCGT	TAG
NB19	GTTCCTCGTGCAGTGTCAAGAGAT	GAC	NB51	GTTGAATGAGCCTACTGGGTCCTC	TAC
NB20	TTGCGTCCTGTTACGAGAACTCAT	GAT	NB52	TGAGAGACAAGATTGTTCGTGGAC	TAT
NB21	GAGCCTCTCATTGTCCGTTCTCTA	GGA	NB53	AGATTCAGACCGTCTCATGCAAAG	TGA
NB22	ACCACTGCCATGTATCAAAGTACG	GGG	NB54	CAAGAGCTTTGACTAAGGAGCATG	TGG
NB23	CTTACTACCCAGTGAACCTCCTCG	GGC	NB55	TGGAAGATGAGACCCTGATCTACG	TGC
NB24	GCATAGTTCTGCATGATGGGTTAG	GGT	NB56	TCACTACTCAACAGGTGGCATGAA	TGT
NB25	GTAAGTTGGGTATGCAACGCAATG	GCA	NB57	GCTAGGTCAATCTCCTTCGGAAGT	TCA
NB26	CATACAGCGACTACGCATTCTCAT	GCG	NB58	CAGGTTACTCCTCCGTGAGTCTGA	TCG
NB27	CGACGGTTAGATTCACCTCTTACA	GCC	NB59	TCAATCAAGAAGGGAAAGCAAGGT	TCC
NB28	TGAAACCTAAGAAGGCACCGTATC	GCT	NB60	CATGTTCAACCAAGGCTTCTATGG	TCT
NB29	CTAGACACCTTGGGTTGACAGACC	GTA	NB61	AGAGGGTACTATGTGCCTCAGCAC	TTA
NB30	TCAGTGAGGATCTACTTCGACCCA	GTG	NB62	CACCCACACTTACTTCAGGACGTA	TTG
NB31	TGCGTACAGCAATCAGTTACATTG	GTC	NB63	TTCTGAAGTTCCTGGGTCTTGAAC	TTC
NB32	CCAGTAGAAGTCCGACAACGTCAT	GTT	NB64	GACAGACACCGTTCATCGACTTTC	TTT

Supplementary Table 6. 20-mer validation library sequences. The randomly chosen 20mer sequences contained within each validation library pool are listed. Each validation pool contained a mixture of 10 hairpin sequences (numbered 1 to 10) with 20 randomized base pairs at the end of the hairpin. Two pools of validation hairpins (each containing 10 unique sequences) can be ligated together to generate 100 (10 x 10) random sequence combinations. Validation pool sequences were randomly generated and intended to provide a sequence diversity (+/- 20 nt surrounding an XNA nt) much greater than what is present in the model training NNN-pools. The smaller library size (100 sequences per ligated pool) and richer sequence diversity made it possible to multiplex all the validation sets while still obtaining sufficient coverage for calculating appropriate statistics. Validation pool sequences are a subset of HP1-[VAL-ID] and HP2-[VAL-ID] hairpin sequences shown in **Supplementary Table 3**. Sequences are shown in 5′ to 3′ direction. Full sequences of hairpins ordered, alongside ligation products generated, can be found in **Supplementary Data 2**.

[VAL-ID]	[VAL]	[VAL-ID]	[VAL]
VAL_A_1	GGTGTATACGTTCTTTGCTC	VAL_D_1	CAAACCGTACGCGGACCCGG
VAL_A_2	GGTATACTTTGCGACATGAT	VAL_D_2	ACGAGCAATCTAAGAAATAA
VAL_A_3	TCAGGTATTACGTCATTCTG	VAL_D_3	AGCGTTTTGGGTACGCATCA
VAL_A_4	TCCTCCTTTTCGACTGACAT	VAL_D_4	TCTGTGCGATTACAAACGCT
VAL_A_5	TGATAGTCAGCATTACGCTC	VAL_D_5	TGAATACAAATGTGTACGTT
VAL_A_6	GATTCTCTACGCGCCAACCG	VAL_D_6	CGCATTAACGGCGAGTAGTA
VAL_A_7	CATCGATCCCCCTAGTATCG	VAL_D_7	GGGCAATGTGCTGACTTAGG
VAL_A_8	GTTTTGCTTCCTCAACCATG	VAL_D_8	AGAAGTGCCCCCCAGCTAGA
VAL_A_9	ACTTAGCACGGTATACTGAA	VAL_D_9	AGAGTTTACTAGATGGTTGA
VAL_A_10	TACCCTCATCTATGGCGATT	VAL_D_10	GAGGTGAGGCTGGGATAAAA
VAL_B_1	CACTCAGTCCAATAACCAGC	VAL_E_1	GATCGTTCGAATCACCATCT
VAL_B_2	TATCTTAGACTGCACTCTTT	VAL_E_2	ATCCGGAGATATCAGCAGGG
VAL_B_3	AAAGATTATGTCGGCTGAAC	VAL_E_3	TCTGCAAAGGAAAGGGTCCA
VAL_B_4	CTGTCATGCGCCAACCATGC	VAL_E_4	CTAGCCCCGCTGAGCGACCT
VAL_B_5	ATTAACAATAGGTGGATGCG	VAL_E_5	GAACTCTACGCCCGCCGCTT
VAL_B_6	ACACGAATTACCATTTCTAA	VAL_E_6	CGAACAGTTGGACAATCGCT
VAL_B_7	GTCTCAGAGAGGCCTGTCTT	VAL_E_7	GTGAGGGAGGGGACTGTCCG
VAL_B_8	TGTTGATCCGCGCGCGAAAA	VAL_E_8	AACGCCAGACCGTTACGATA
VAL_B_9	GTAACCTCAAAACTATTCAA	VAL_E_9	ACTGTTAGCCCTCTCTAGAG
VAL_B_10	AAATTGTATGCATTTGACCC	VAL_E_10	TTAGTCGGAAGTATCTGTAC
VAL_C_1	CGAGTTAATCCCCTATGCGT	VAL_F_1	CGGTCTGCCGGTTCGAGCAC
VAL_C_2	CTGGGCAAAGGAACGGTACT	VAL_F_2	GGGGCACTGCCTCCGATGAT
VAL_C_3	ATTGTGGAGTGCCAGCCCCC	VAL_F_3	GCTTAGACATCTCGACCTTA
VAL_C_4	TTCGAAGCCGTGATTCGACA	VAL_F_4	ACAATTCAGTGGTAGTAGAC
VAL_C_5	CGCGAGGTATGCTACACATC	VAL_F_5	CGTAAATGGGGCAAAGGGAA
VAL_C_6	TTCGGCCTGGTCGTGTTAAC	VAL_F_6	GGCCACGGGAACCTCTGCGG
VAL_C_7	ACTGCGTGATAGCTGATTTC	VAL_F_7	AAAATCTTAAGAGCGGGAAG
VAL_C_8	CGCGGTTTTCCGATGTCGCG	VAL_F_8	ATCAATCGTTCATACCAAGG
VAL_C_9	TCAAACCCGACGGAGTTATA	VAL_F_9	TTCCTCCACAGGCATGGACT
VAL_C_10	AGAACTGCTGCCTATTTGGT	VAL_F_10	ACATCGGGGCAAGTATTATT

Supplementary Table 7. 12-letter DNA construction sequences. Sequences of hairpins used to build 12-letter DNA. Two tailed hairpins can be ligated together to generate a sequence with a single xenonucleotide insertion. Four single insertion constructs can undergo Golden Gate ligation to form a DNA sequence containing all 12 letters. Table shows barcodes for each oligo that links to the variable 3 nt sequence on the 3′-end and the xenonucleotide tailed on the 3′-end (bold), as well as restriction site sequences (red, bold). Sequences are shown in 5′ to 3′ direction.

HP	Sequence
HP12-A1	/5Phos/AGATCAGC**GAAAGTTGTCGGTGTCTTTGTGGATATC**GAGCTCTTTCTGAGCTC**GATATCCACAAAGACACCGACAACTTTC**GCTGATCT
HP12-A2	/5Phos/AGTTCAGC**ACAGACGACTACAAACTGCCGC**GTCTTCCAGCTCTTTCTGAGCTGGAAGAC**GCGGCAGTTTGTAGTCGTCTGT**GCTGAACT
HP12-A3	/5Phos/ACTTCAGC**TGTCCCAGTTACCAGTGGCAGG**GTCTTCCAGCTCTTTCTGAGCTGGAAGAC**CCTGCCACTGGTAACTGGGACA**GCTGAAGT
HP12-A4	/5Phos/CAGTCAGC**CAGGGAAACACGATAGGCAACC**GTCTTCCAGCTCTTTCTGAGCTGGAAGAC**GGTTGCCTATCGTGTTTCCCTG**GCTGACTG
HP12-A5	/5Phos/GTCTCAGC**CTTGTCCAGGGTTTGTTTGCGG**GTCTTCCAGCTCTTTCTGAGCTGGAAGAC**CCGCAAACAAACCCTGGACAAG**GCTGAGAC
HP12-A6	/5Phos/ATCTCAGC**GACTACTTTCTGCCTTATCACC**GTCTTCCAGCTCTTTCTGAGCTGGAAGAC**GGTGATAAGGCAGAAAGTAGTC**GCTGAGAT
HP12-A7	/5Phos/AGCTCAGC**GTGGGAATGAATCCTTTGATGG**GTCTTCCAGCTCTTTCTGAGCTGGAAGAC**CCATCAAAGGATTCATTCCCAC**GCTGAGCT
HP12-A8	/5Phos/AGTTCAGC**ACGTAACTTGGTTTGTTCCCTGAGTACT**CAGCTATTTCTTAGCTG**AGTACTCAGGGAACAAACCAAGTTACGT**GCTGAACT
HP12-B1	/5Phos/AATTCAGC**CTAGGCACAGCGAGTCTTGGTTGATATC**GAGCTCTTTCTGAGCTC**GATATCAACCAAGACTCGCTGTGCCTAG**GCTGAATT
HP12-B2	/5Phos/CGCTCAGC**AAAGGTTTCAACGCTTTGCCGC**GTCTTCCAGCTCTTTCTGAGCTGGAAGAC**GCGGCAAAGCGTTGAAACCTTT**GCTGAGCG
HP12-B3	/5Phos/GTTTCAGC**TGTCCCAGTTACCAGTGGCAGG**GTCTTCCAGCTCTTTCTGAGCTGGAAGAC**CCTGCCACTGGTAACTGGGACA**GCTGAAAC
HP12-B4	/5Phos/AGTTCAGC**CAGGGAAACACGATAGGCAACC**GTCTTCCAGCTCTTTCTGAGCTGGAAGAC**GGTTGCCTATCGTGTTTCCCTG**GCTGAACT
HP12-B5	/5Phos/GAATCAGC**CTTGTCCAGGGTTTGTTTGCGG**GTCTTCCAGCTCTTTCTGAGCTGGAAGAC**CCGCAAACAAACCCTGGACAAG**GCTGATTC
HP12-B6	/5Phos/AGCTCAGC**GACTACTTTCTGCCTTATCACC**GTCTTCCAGCTCTTTCTGAGCTGGAAGAC**GGTGATAAGGCAGAAAGTAGTC**GCTGAGCT
HP12-B7	/5Phos/GAGTCAGC**GTGGGAATGAATCCTTTGATGG**GTCTTCCAGCTCTTTCTGAGCTGGAAGAC**CCATCAAAGGATTCATTCCCAC**GCTGACTC
HP12-B8	/5Phos/AGTTCAGC**ACGTAACTTGGTTTGTTCCCTGAGTACT**CAGCTATTTCTTAGCTG**AGTACTCAGGGAACAAACCAAGTTACGT**GCTGAACT

/5Phos/ = 5′-PO_4

Supplementary Table 8. Optimized reaction components and conditions used for XNA tailing. Polymerase choice, polymerase amount, reaction time, and reaction temperature for each dxNTP and dNTP tailed to blunt-ended hairpin oligos or oligo pools (**Supplementary Table 2, 3**). All reactions excluding N = A also contain 0.005 U/µL of YiPP).

N/X	Polymerase	[Pol] U/µL	[dNTP] mM	Time	Temp
A	Taq	0.24	2.38	1h	60°C
T	Therminator	0.29	1.19	4h	60°C
G	Therminator	0.29	2.38	1h	60°C
C	Therminator	0.29	1.19	8h	60°C
B	KF(exo-)	0.71	1.19	8h	37°C
S^n	Therminator	0.29	1.19	4h	60°C
S^c	Therminator	0.29	1.19	16h	60°C
P	KF(exo-)	0.71	1.19	8h	37°C
Z	Therminator	0.29	1.19	4h	60°C
X^t	Therminator	0.29	1.19	4h	60°C
K^n	Therminator	0.29	1.19	4h	60°C
J	Therminator	0.29	1.19	4h	60°C
V	Therminator	0.29	1.19	4h	60°C

Supplementary Table 9. XNA tailing extent of reaction. Tailing extent reaction estimate was calculated as the percent relative intensity of the product band, relative to intensities in each lane for blunt-ligation (upper band) and product (lower band). Tailing reactions were optimized to eliminate residual starting material (blunt-end DNA), which would be a major source of non-specific ligation in subsequent reactions. Band intensity was estimated using ImageJ with background intensity subtraction. Estimates presented are for tailing reactions shown in **Fig. 2** using optimized reaction conditions (see **Supplementary Fig. 17** for the full gel). Note that these estimates present an upper limit of tailing success, and more conservative estimates (< 5% remaining) should be used if quantifying by gel.

Base	Blunt-end remaining
A	1%
T	1%
G	<1%
C	<1%
B	1%
S^n	2%
S^c	2%
P	<1%
Z	<1%
X^t	<1%
K^n	<1%
J	<1%
V	<1%

Supplementary Table 10. Optimized reaction components and conditions used for XNA ligation. DNA ligase choice and ligase amount for XNA ligation reactions between complementary single nucleotide 3′-overhang of XNA base pairs. All ligation reactions were carried out at 16 °C for 16 h.

Base pair	Ligase	[Ligase]
BS^n	T7	273 U/μL
BS^c	T7	750 U/μL
PZ	T4	36 U/μL
X^tK^n	T3	272 U/μL
JV	T3	272 U/μL
Blunt	T4	36 U/μL

Supplementary Table 11. XNA ligation yield. Ligation yield was calculated as the relative intensity of the product band, setting negative control as 0% yield and starting material band intensity as 100% yield. Ligation yield for individual sequences is known to have a sequence context dependence. Estimates presented are for ligation reactions shown in **Fig. 2** using optimized reaction conditions (see **Supplementary Fig. 17** for the full gel).

Base pair	Ligation yield estimate
BS^n	73%
BS^c	7%
PZ	53%
X^tK^n	31%
JV	15%
Blunt	72%

Supplementary Table 12. Constructs generated through XNA tailing. Construction of single XNA nt tailed NNN-library hairpins, validation library hairpins, and 12-letter DNA hairpins by XNA tailing of different oligos and oligo pools (**Supplementary Table 2, 3, 7**).

HP Substrate Name(s)	dxNTP	Product Name(s)
HP_v1-NNN-P3, HP_v1-NNN-P1	B	HP_v1-NNN-P3-B, HP_v1-NNN-P1-B
HP_v2-NNN-P4, HP_v2-NNN-P2	S^n	HP_v2-NNN-P4-S^n, HP_v1-NNN-P2-S^n
HP_v2-NNN-P4, HP_v2-NNN-P2	S^c	HP_v2-NNN-P4-S^c, HP_v1-NNN-P2-S^c
HP_v1-NNN-P3	P	HP_v1-NNN-P3-P
HP_v2-NNN-P4	Z	HP_v2-NNN-P4-Z
HP_v1-NNN-P3, HP_v1-NNN-P1	X^t	HP_v1-NNN-P3-X^t, HP_v1-NNN-P1-X^t
HP_v2-NNN-P4, HP_v2-NNN-P2	K^n	HP_v2-NNN-P4-K^n, HP_v1-NNN-P2-K^n
HP_v1-NNN-P3	J	HP_v1-NNN-P3-J
HP_v2-NNN-P4	V	HP_v2-NNN-P4-V
HP1-Val-A, HP1-Val-E	B	HP1-Val-A-B, HP1-Val-E-B
HP2-Val-B	S^n	HP2-Val-B-S^n
HP2-Val-F	S^c	HP2-Val-F-S^c
HP2-Val-B	P	HP2-Val-B-P
HP1-Val-C	Z	HP1-Val-C-Z
HP1-Val-C	X^t	HP1-Val-C-X^t
HP2-Val-D	K^n	HP2-Val-D-K^n
HP2-Val-D, HP1-Val-A	J	HP2-Val-D-J, HP1-Val-A-J
HP1-Val-E, HP1-Val-C	V	HP1-Val-E-V, HP1-Val-C-V
HP12-A1, HP12-B1	B	HP12-A1-B, HP12-B1-B
HP12-A2	S^c	HP12-A2-S^c
HP12-B2	S^n	HP12-B2-S^n
HP12-A3, HP12-B3	P	HP12-A3-P, HP12-B3-P
HP12-A4, HP12-B4	Z	HP12-A4-Z, HP12-B4-Z
HP12-A5, HP12-B5	X^t	HP12-A5-X^t, HP12-B5-X^t
HP12-A6, HP12-B6	K^n	HP12-A6-K^n, HP12-B6-K^n
HP12-A7, HP12-B7	J	HP12-A7-J, HP12-B7-J
HP12-A8, HP12-B8	V	HP12-A8-V, HP12-B8-V

Supplementary Table 13. Constructs generated through XNA ligation. Assembly of single XNA-bp constructs from two oligos or oligo pools containing a single nucleotide overhang (**Supplementary Table 12)**. NNN$\underline{N}$NNN libraries were built to obtain full coverage over an NNN$\underline{N}$NNN heptamer region for building kmer models. Val-20 validation library was built to sample randomized 20-mer regions for testing kmer models. 12-letter DNA was built such that all 12 bases could be sequenced in a single read. Library size calculation includes consideration of both sense and antisense sequences as independent reads. N/A = not applicable.

HP substrate 1	HP substrate 2	Product	Library size (#)	Usage
HP_v1-NNN-P3-B	HP_v2-NNN-P4-S^n	HP-NNN-BS^n	4096 x 2	Model building
HP_v1-NNN-P3-B	HP_v2-NNN-P4-S^c	HP-NNN-BS^c	4096 x 2	Model building
HP_v1-NNN-P3-P	HP_v2-NNN-P4-Z	HP-NNN-PZ	4096 x 2	Model building
HP_v1-NNN-P3-X^t	HP_v2-NNN-P4-K^n	HP-NNN-X^tK^n	4096 x 2	Model building
HP_v1-NNN-P3-J	HP_v2-NNN-P4-V	HP-NNN-JV	4096 x 2	Model building
HP_v1-NNN-P1	HP_v2-NNN-P2	HP-NNN-blunt	4096 x 1	Model building
HP_v1-NNN-P1-B	HP_v2-NNN-P2-S^n	HP-NNN-BS^n-2	4096 x 2	Replicate set
HP_v1-NNN-P1-B	HP_v2-NNN-P2-S^c	HP-NNN-BS^c-2	4096 x 2	Replicate set
HP_v1-NNN-P3-P	HP_v2-NNN-P4-Z	HP-NNN-PZ-2	4096 x 2	Replicate set
HP_v1-NNN-P1-X^t	HP_v2-NNN-P2-K^n	HP-NNN-X^tK^n-2	4096 x 2	Replicate set
HP_v1-NNN-P3-J	HP_v2-NNN-P4-V	HP-NNN-JV-2	4096 x 2	Replicate set
HP_v1-NNN-P1	HP_v2-NNN-P2	HP-NNN-blunt-2	4096 x 1	Validation set
HP1-Val-A-B	HP2-Val-B-S^n	Val-AB-BS^n	100 x 2	Validation set
HP1-Val-E-B	HP2-Val-F-S^c	Val-EF-BS^c	100 x 2	Validation set
HP1-Val-B-B	HP2-Val-E-S^c	Val-BE-BS^c	100 x 2	Validation set
HP2-Val-B-P	HP1-Val-C-Z	Val-BC-PZ	100 x 2	Validation set
HP1-Val-C-X^t	HP2-Val-D-K^n	Val-CD-X^tK^n	100 x 2	Validation set
HP2-Val-F-X^t	HP1-Val-A-K^n	Val-FA-X^tK^n	100 x 2	Validation set
HP2-Val-D-J	HP1-Val-E-V	Val-DE-JV	100 x 2	Validation set
HP1-Val-A-J	HP1-Val-C-V	Val-AC-JV	100 x 2	Validation set
HP12-A1-B	HP12-A2-S^c	HP12-BS^c-A	N/A	12-letter DNA
HP12-B1-B	HP12-B2-S^n	HP12-BS^n-B	N/A	12-letter DNA
HP12-A3-P	HP12-A4-Z	HP12-PZ-A	N/A	12-letter DNA
HP12-B3-P	HP12-B4-Z	HP12-PZ-B	N/A	12-letter DNA
HP12-A5-X^t	HP12-A6-K^n	HP12-X^tK^n-A	N/A	12-letter DNA
HP12-B5-X^t	HP12-B6-K^n	HP12-X^tK^n-B	N/A	12-letter DNA
HP12-A7-J	HP12-A8-V	HP12-JV-A	N/A	12-letter DNA
HP12-B7-J	HP12-B8-V	HP12-JV-B	N/A	12-letter DNA

Supplementary Table 14. Nanopore run overview. Run IDs, contents of run, flow cells, and flow cell chemistry used to generate training and validation datasets.

Run ID	Run contents	Flow cell
NNN-BS^n-NNN	HP-NNN-BS^n	Flongle R9.4.1
NNN-BS^c-NNN	HP-NNN-BS^c	Flongle R9.4.1
NNN-PZ-NNN	HP-NNN-PZ	Flongle R9.4.1
NNN-X^{t}K^n-NNN	HP-NNN-X^{t}K^n	Flongle R9.4.1
NNN-JV-NNN	HP-NNN-JV	Flongle R9.4.1
NNN-BS^n-NNN-2	HP-NNN-BS^n-2	Flongle R9.4.1
NNN-BS^c-NNN-2	HP-NNN-BS^c-2	Flongle R9.4.1
NNN-PZ-NNN-2	HP-NNN-PZ-2	Flongle R9.4.1
NNN-X^{t}K^n-NNN-2	HP-NNN-X^{t}K^n-2	Flongle R9.4.1
NNN-JV-NNN-2	HP-NNN-JV-2	Flongle R9.4.1
NNN-NNN-1	HP-NNN-blunt	Flongle R9.4.1
NNN-NNN-2	HP-NNN-blunt-2	Flongle R9.4.1
Val-BSPZJVXK	Val-AB-BS^n, Val-EF-BS^c, Val-BC-PZ, Val-CD-X^{t}K^n, Val-DE-JV, Val-AC-JV	MinION R9.4.1
Val-BSXK	Val-BE-BS^c, Val-FA-X^{t}K^n	MinION R9.4.1
12L-DNA-setA	HP12-BS^{c}PZJVX^{t}K^n (S^{c}uper-12)	Flongle R9.4.1
12L-DNA-setB	HP12-BS^{n}PZJVX^{t}K^n (S^{n}uper-12)	Flongle R9.4.1

Supplementary Table 15. Nanopore run read summary for model building. Summary of total reads obtained from each run for model building, listed by Run ID. In the xenomorph processing pipeline, reads are first basecalled by guppy then aligned to a reference using minimap2. Reads that align to the reference are further filtered by reads that fully align to barcoded heteroligation products and subsequently analyzed for kmer model building and kmer model validation.

Run ID	Model built	Total reads	Reads pass filter
NNN-BS^n-NNN	BS^n	351 k	50 k
NNN-BS^c-NNN	BS^c	156 k	19 k
NNN-PZ-NNN	PZ	783 k	102 k
NNN-X^{t}K^n-NNN	X^{t}K^n	374 k	46 k
NNN-JV-NNN-4h	JV	810 k	43 k
NNN-NNN-1	ATGC	655 k	80 k

Supplementary Table 16. Heptamer, kmer, and level structure used in kmer model. Heptamer sequence contains every combination of canonical nucleotide (N = A, T, G, C) and any XNA base (N = B, S^n, S^c, P, Z, J, V, X^t, K^n for XNA or N = A, T, G, C for canonical). Kmers of k = 4 are extracted from heptamer sequences and mapped to the signal level using a (-1, 0, +1, +2) sequence-to-level mask. The kmer sequences are assigned signal levels matching the 0th position nucleotide. Red letters highlight the sliding window within the heptamer sequence that corresponds to the kmer sequence. For a given heptamer sequence *S,* corresponding kmers are numbered k_1 to k_4 from left to right.

Heptamer(*S*)	*Kmer* seq	level	*kmer i*
NNNNNNN	NNNN---	-N-----	k_1
NNNNNNN	-NNNN--	--N----	k_2
NNNNNNN	--NNNN-	---N---	k_3
NNNNNNN	---NNNN	----N--	k_4

Supplementary Table 17. Column header and description of `xenomorph preprocess` output file.

Col	Header	Description	Format
1	read_ID	FAST5 file name	String
2	reference_sequence	Name of mapped ref FASTA sequence	String
3	q-score	Average q-score of matched sequence	Float
4	read_signal_match_score	Signal match score of matched sequence	Float
5	read_signal_match_start	Signal match start position in ref FASTA	Int
6	read_signal_match_end	Signal match end position in ref FASTA	Int
7	read_pos_relative_to_raw	Raw signal position where match starts	Int
8	read_reference_locus	Ref sequence of signal extracted region	String
9	read_substitution_base	Canonical base used for segmentation	String
10	read_xna_strand	Strand of signal extracted region (+,-)	String
11	read_xna_sequence	Sequence of signal extracted region	String
12	read_xna	XNA base from reference at center	String
13	read_xna_position	Position of XNA base in reference sequence	Int
14	read_levels	Array of norm. median signal level	Float Array

Supplementary Table 18. Column header and description of `xenomorph morph` output file.

Col	Header	Description	Format
1-14	*See Supplementary Table 17*	*See Supplementary Table 17*	-
15	xeno_basecall	Most likely base at read_xna_position	String
16	log_likelihood_ratio	Log likelihood ratio of most likely base relative to second most likely base	Float
17	model_sigma	Model standard deviation values (global or kmer-specific) used in alternative hypothesis testing	Float
18	model_mean	Model mean values (mean or median) used in alternative hypothesis testing	String
19	model_file	Kmer model used to perform alternative hypothesis testing	String

Supplementary Table 19. Recall benchmarking for 4-nt kmer XNA models. Tabulation of estimated recall for 4-nt XNA kmer models (mean model) tested against the Val-20 set (n = 5,000 reads for each base).

recall

	N vs §			***N vs ATGC***		
**XNA model**	**Per-read**	**Consensus**	**Per-sequence**	**Per-read**	**Consensus**	**Per-sequence**
**B**	0.77	0.89	0.87	0.74	0.91	0.81
S^n	0.82	0.81	0.78	0.71	0.71	0.70
S^c	0.75	0.73	0.78	0.60	0.61	0.61
**P**	0.79	0.93	0.80	0.68	0.89	0.74
**Z**	0.87	0.99	0.92	0.83	0.99	0.92
**J**	0.60	0.63	0.74	0.55	0.59	0.73
**V**	0.82	0.86	0.88	0.74	0.86	0.84
X^t	0.65	0.84	0.74	0.61	0.81	0.72
K^n	0.70	0.69	0.57	0.62	0.54	0.47

Supplementary Table 20. Specificity benchmarking for 4-nt kmer XNA models. Tabulation of specificity (1-FDR) for the 4-nt XNA kmer models tested against a DNA library without any XNAs incorporated (NNN-blunt; n = 50,000 reads for each base). Per-read (Per-Read), per-read consensus (Consensus), and per-sequence consensus (Per-sequence) values calculated from sequences with at least 10 mapped reads.

specificity (1 – *FDR*)

	N vs §			***N vs ATGC***		
**XNA model**	**Per-read**	**Consensus**	**Per-sequence**	**Per-read**	**Consensus**	**Per-sequence**
**B**	0.89	0.96	0.98	0.97	>0.99	>0.99
S^n	0.83	0.95	0.88	0.96	0.99	0.99
S^c	0.85	0.95	0.94	0.95	0.98	0.98
**P**	0.85	0.96	0.87	0.96	0.99	>0.99
**Z**	0.93	>0.99	0.96	0.98	>0.99	>0.99
**J**	0.83	0.89	0.95	0.94	0.95	0.94
**V**	0.87	0.97	0.93	0.96	0.99	0.97
X^t	0.86	0.95	0.97	0.96	0.99	0.99
K^n	0.80	0.89	0.93	0.94	0.97	0.97

Supplementary Table 21. Recall benchmarking for 4-nt and 12-letter kmer XNA model. Tabulation of estimated recall for a 4-nt XNA kmer model tested Val-20 sets using a full 12-letter (ATGCBS^{c}PZXKJV) kmer model for alternative hypothesis testing, from confusion matrix shown in **Fig. 4**. In '*N* vs *S*' comparisons, '*S*' denotes the most similar standard base as determined by guppy basecall. Mean kmer signal levels and outlier-robust log-likelihood ratios were used for the base classification. Box highlights base chosen from picking the most likely nucleobase among any purine or pyrimidine.

Per-read recall of Val-20

	XNA in sequence							
**Base called**	**B**	**S^c**	**P**	**Z**	**X^t**	**K^n**	**J**	**V**
**B**	**0.53**	0.01	0.01	0.00	0.11	0.01	0.03	0.02
**S^c**	0.06	**0.43**	0.05	0.01	0.01	0.02	0.01	0.07
**P**	0.01	0.07	**0.46**	0.00	0.01	0.01	0.01	0.08
**Z**	0.02	0.01	0.00	**0.77**	0.01	0.00	0.09	0.00
**X^t**	0.04	0.03	0.02	0.00	**0.45**	0.02	0.02	0.02
**K^n**	0.08	0.05	0.09	0.00	0.01	**0.72**	0.02	0.04
**J**	0.04	0.01	0.00	0.09	0.01	0.00	**0.54**	0.01
**V**	0.01	0.16	0.17	0.00	0.02	0.01	0.01	**0.62**
**A**	0.18	0.03	0.05	0.00	0.34	0.07	0.01	0.02
**T**	0.01	0.05	0.02	0.04	0.02	0.00	0.03	0.06
**G**	0.01	0.14	0.12	0.00	0.01	0.13	0.01	0.06
**C**	0.01	0.01	0.01	0.08	0.01	0.01	0.22	0.01

Supplementary Table 22. Template sequences and primer sequences used for PCR of P≡Z base pair. Synthetic oligo template sequence with a P≡Z base pair (red, bold), received from Firebird Biosciences (Alachua, Fl). Oligo template sequences are hybridized prior to use as a PCR template. Primer sequences are used to amplify the template: each condition used a different barcoded reverse primer (PCR_Amp_R1: Equimolar; PCR_Amp_R2: Optimal; PCR_Amp_R3: No dxNTP; PCR_Amp_R4: Limiting). All conditions used the same forward primer (PCR_Amp_F). Sequences are shown in 5′ to 3′ direction.

Name	Template Sequence
PCR_Template_P	GGTCTGGTGCCACTGGTAACTGGGACAGCTGAAGT**P**CAGTCAGCCAGGGAAACACGATAGGCAACCACACC
PCR_Template_Z	GGTGTGGTTGCCTATCGTGTTTCCCTGGCTGACTG**Z**ACTTCAGCTGTCCCAGTTACCAGTGGCACCAGACC
**Name**	**Primer Sequence**
PCR_Amp_F	CGATTCCACAAAGACACCGACAACTTTCTTGGTCTGGTGCCACTGGT
PCR_Amp_R1	CGATTCAAGGATTCATTCCCACGGTAACACGGTGTGGTTGCCTATCGTG
PCR_Amp_R2	CGATTCACGTAACTTGGTTTGTTCCCTGAAGGTGTGGTTGCCTATCGTG
PCR_Amp_R3	CGATTCAACCAAGACTCGCTGTGCCTAGTTGGTGTGGTTGCCTATCGTG
PCR_Amp_R4	CGATTCGAGAGGACAAAGGTTTCAACGCTTGGTGTGGTTGCCTATCGTG

Supplementary Table 23. Thermocycler settings used for PCR of P≡Z base pair. Thermocycler conditions used to amplify P≡Z template. 25 total cycles were performed.

Step	Temperature (°C)	Time
1	95	2 min
2	95	15 s
3	58.5	15 s
4	72	10 s
**5**	**Cycle steps 2-4**	**24x**
6	72	1 min
7	10	Hold

Supplementary Table 24. 12-letter DNA Sequence (S^cuper-12 and S^nuper-12). Sequences of 12-letter DNA as prepared for nanopore sequencing. XNA positions are bolded in orange. Two sets of sequences were constructed, with set A containing S^c and set B containing S^n. After Golden Gate ligation, both sets were digested using restriction enzymes at either restriction site 5′-GATATC-3′ or 5′-AGTACT-3′ to remove one of the hairpin ends. By removing one hairpin, a singular blunt end is generated for nanopore DNA preparation; this allows for subsequent sequencing of both sense and antisense strands in a single nanopore sequencing event. Since 4-nt kmer models were built from dsDNA data, basecalling uses signals collected from dsDNA portion of each read. Sequences shown in 5′ to 3′ direction.

12-letter construct	Sequence
12L-DNA-setA_1 (S^cuper-12)	ATCCACAAAGACACCGACAACTTTCGCTGATCT**B**AGTTCAGCACAGACGACTACAAACTGCCACTGGTAACTG<br>GGACAGCTGAAGT**P**CAGTCAGCCAGGGAAACACGATAGGCAAACAAACCCTGGACAAGGCTGAGAC**X**ATCTCA<br>GCGACTACTTTCTGCCTTATCAAAGGATTCATTCCCACGCTGAGCT**J**AGTTCAGCACGTAACTTGGTTTGTTC<br>CCTGAGTACTCAGCTATTTCTTAGCTGAGTACTCAGGGAACAAACCAAGTTACGTGCTGAACT**V**AGCTCAGCG<br>TGGGAATGAATCCTTTGATAAGGCAGAAAGTAGTCGCTGAGAT**K**GTCTCAGCCTTGTCCAGGGTTTGTTTGCC<br>TATCGTGTTTCCCTGGCTGACTG**Z**ACTTCAGCTGTCCCAGTTACCAGTGGCAGTTTGTAGTCGTCTGTGCTGA<br>ACT**S**AGATCAGCGAAAGTTGTCGGTGTCTTTGTGGAT
12L-DNA-setA_2 (S^cuper-12)	ACTCAGGGAACAAACCAAGTTACGTGCTGAACT**V**AGCTCAGCGTGGGAATGAATCCTTTGATAAGGCAGAAAG<br>TAGTCGCTGAGAT**K**GTCTCAGCCTTGTCCAGGGTTTGTTTGCCTATCGTGTTTCCCTGGCTGACTG**Z**ACTTCA<br>GCTGTCCCAGTTACCAGTGGCAGTTTGTAGTCGTCTGTGCTGAACT**S**AGATCAGCGAAAGTTGTCGGTGTCTT<br>TGTGGATATCGAGCTCTTTCTGAGCTCGATATCCACAAAGACACCGACAACTTTCGCTGATCT**B**AGTTCAGCA<br>CAGACGACTACAAACTGCCACTGGTAACTGGGACAGCTGAAGT**P**CAGTCAGCCAGGGAAACACGATAGGCAAA<br>CAAACCCTGGACAAGGCTGAGAC**X**ATCTCAGCGACTACTTTCTGCCTTATCAAAGGATTCATTCCCACGCTGA<br>GCT**J**AGTTCAGCACGTAACTTGGTTTGTTCCCTGAGT
12L-DNA-setB_1 (S^nuper-12)	ATCAACCAAGACTCGCTGTGCCTAGGCTGAATT**B**CGCTCAGCAAAGGTTTCAACGCTTTGCCACTGGTAACTG<br>GGACAGCTGAAAC**P**AGTTCAGCCAGGGAAACACGATAGGCAAACAAACCCTGGACAAGGCTGATTC**X**AGCTCA<br>GCGACTACTTTCTGCCTTATCAAAGGATTCATTCCCACGCTGACTC**J**AGTTCAGCACGTAACTTGGTTTGTTC<br>CCTGAGTACTCAGCTATTTCTTAGCTGAGTACTCAGGGAACAAACCAAGTTACGTGCTGAACT**V**GAGTCAGCG<br>TGGGAATGAATCCTTTGATAAGGCAGAAAGTAGTCGCTGAGCT**K**GAATCAGCCTTGTCCAGGGTTTGTTTGCC<br>TATCGTGTTTCCCTGGCTGAACT**Z**GTTTCAGCTGTCCCAGTTACCAGTGGCAAAGCGTTGAAACCTTTGCTGA<br>GCG**S**AATTCAGCCTAGGCACAGCGAGTCTTGGTTGAT
12L-DNA-setB_2 (S^nuper-12)	ACTCAGGGAACAAACCAAGTTACGTGCTGAACT**V**GAGTCAGCGTGGGAATGAATCCTTTGATAAGGCAGAAAG<br>TAGTCGCTGAGCT**K**GAATCAGCCTTGTCCAGGGTTTGTTTGCCTATCGTGTTTCCCTGGCTGAACT**Z**GTTTCA<br>GCTGTCCCAGTTACCAGTGGCAAAGCGTTGAAACCTTTGCTGAGCG**S**AATTCAGCCTAGGCACAGCGAGTCTT<br>GGTTGATATCGAGCTCTTTCTGAGCTCGATATCAACCAAGACTCGCTGTGCCTAGGCTGAATT**B**CGCTCAGCA<br>AAGGTTTCAACGCTTTGCCACTGGTAACTGGGACAGCTGAAAC**P**AGTTCAGCCAGGGAAACACGATAGGCAAA<br>CAAACCCTGGACAAGGCTGATTC**X**AGCTCAGCGACTACTTTCTGCCTTATCAAAGGATTCATTCCCACGCTGA<br>CTC**J**AGTTCAGCACGTAACTTGGTTTGTTCCCTGAGT

Supplementary Table 25. S^cuper-12 per-read recall confusion matrix values. Tabulation of per-read recall results using the 4-nt XNA kmer model for S^cuper-12, from confusion matrices shown in **Fig. 5**. Table shows: (left) fraction of base called at each xenonucleotide position using the full 12-letter supernumerary model; (right) base called using model with simplified priors, where ‡ denotes the xenonucleotide at position called, and § denotes the most similar standard base called instead. Box highlights base pair chosen from picking the most likely nucleobase among any purine or pyrimidine set, then fixing complementary base.

Base called – S^cuper-12

Base in sequence	B	S^c	P	Z	X^t	K^n	J	V	A	T	G	C	‡	§
B	0.59	0.06	0.00	0.06	0.11	0.01	0.01	0.01	0.15	0.00	0.00	0.00	0.71	0.29
S^c	0.02	0.46	0.09	0.02	0.04	0.01	0.00	0.23	0.05	0.02	0.06	0.01	0.87	0.13
P	0.00	0.01	0.83	0.00	0.01	0.00	0.00	0.10	0.01	0.00	0.03	0.00	0.92	0.08
Z	0.03	0.00	0.01	0.69	0.03	0.01	0.12	0.00	0.00	0.03	0.00	0.08	0.78	0.22
X^t	0.01	0.03	0.00	0.01	0.39	0.06	0.00	0.08	0.38	0.02	0.00	0.01	0.48	0.52
K^n	0.02	0.02	0.00	0.01	0.01	0.82	0.01	0.07	0.01	0.01	0.01	0.01	0.95	0.05
J	0.01	0.03	0.00	0.07	0.01	0.01	0.67	0.03	0.01	0.11	0.00	0.06	0.81	0.19
V	0.00	0.04	0.03	0.00	0.00	0.00	0.00	0.89	0.01	0.00	0.02	0.00	0.93	0.07

Supplementary Table 26. S^nuper-12 per-read recall confusion matrix values. Tabulation of per-read recall results using the 4-nt XNA kmer model for S^nuper-12, from confusion matrices shown in **Fig. 5**. Table shows: (left) fraction of base called at each xenonucleotide position using the full 12-letter supernumerary model; (right) base called using model with simplified priors, where ‡ denotes the xenonucleotide at position called, and § denotes the most similar standard base called instead. Box highlights base pair chosen from picking the most likely nucleobase among any purine or pyrimidine set, then fixing complementary base.

Base called – S^nuper-12

Base in sequence	B	S^n	P	Z	X^t	K^n	J	V	A	T	G	C	‡	§
B	0.74	0.01	0.02	0.02	0.08	0.02	0.01	0.01	0.03	0.01	0.05	0.01	0.80	0.20
S^n	0.02	0.51	0.00	0.00	0.03	0.03	0.02	0.35	0.04	0.00	0.01	0.01	0.91	0.09
P	0.01	0.05	0.66	0.00	0.06	0.02	0.01	0.06	0.04	0.01	0.08	0.01	0.78	0.22
Z	0.00	0.01	0.00	0.89	0.00	0.01	0.01	0.02	0.00	0.05	0.00	0.01	0.96	0.04
X^t	0.01	0.06	0.07	0.00	0.37	0.25	0.01	0.07	0.13	0.00	0.00	0.01	0.73	0.27
K^n	0.10	0.14	0.02	0.00	0.32	0.14	0.00	0.02	0.09	0.00	0.12	0.04	0.67	0.33
J	0.06	0.03	0.01	0.05	0.02	0.00	0.69	0.02	0.00	0.00	0.00	0.11	0.79	0.21
V	0.00	0.09	0.01	0.00	0.00	0.00	0.00	0.85	0.01	0.00	0.03	0.00	0.93	0.07

Supplementary Table 27. Tabulation of per-read recall from simulated signal levels for the standard genetic code (A, T, G, C). Information regarding read simulation can be found in the Supplementary Note section.

Standard code

	Base simulated			
**DNA recalled**	**A**	**T**	**G**	**C**
**A**	**0.81**	0.00	0.16	0.02
**T**	0.00	**0.96**	0.00	0.04
**G**	0.16	0.00	**0.83**	0.00
**C**	0.02	0.03	0.00	**0.94**

Supplementary Table 28. Tabulation of per-read recall from simulated signal levels for the isoG/isoC code (A, T, G, C, B, S^n).

isoG/isoC code

	Base simulated					
**XNA recalled**	**A**	**T**	**G**	**C**	**B**	**S^n**
**A**	**0.67**	0.00	0.14	0.01	0.09	0.06
**T**	0.00	**0.96**	0.00	0.04	0.00	0.00
**G**	0.14	0.00	**0.76**	0.00	0.03	0.07
**C**	0.01	0.03	0.00	**0.89**	0.06	0.00
**B**	0.10	0.00	0.03	0.06	**0.80**	0.03
**S^n**	0.07	0.00	0.07	0.00	0.03	**0.83**

Supplementary Table 29. Tabulation of per-read recall from simulated signal levels for the hachimoji code (A, T, G, C, B, S^c, P, Z).

Hachimoji code

	Base simulated							
**XNA recalled**	**A**	**T**	**G**	**C**	**B**	**S^c**	**P**	**Z**
**A**	**0.63**	0.00	0.13	0.01	0.08	0.08	0.04	0.02
**T**	0.00	**0.95**	0.00	0.03	0.00	0.01	0.00	0.01
**G**	0.13	0.00	**0.64**	0.00	0.02	0.06	0.13	0.00
**C**	0.01	0.03	0.00	**0.75**	0.04	0.03	0.00	0.13
**B**	0.09	0.00	0.03	0.04	**0.76**	0.05	0.01	0.04
**S^c**	0.08	0.01	0.06	0.03	0.05	**0.71**	0.04	0.01
**P**	0.04	0.00	0.14	0.00	0.01	0.05	**0.78**	0.00
**Z**	0.02	0.01	0.00	0.13	0.04	0.01	0.00	**0.79**

Supplementary Table 30. Tabulation of per-read recall from simulated signal levels for the 12-base S^{n}upernumerary code (A, T, G, C, B, S^n P, Z, X^t, K^n, J, V).

S^{n}upernumerary code

	Base simulated											
**XNA recalled**	**A**	**T**	**G**	**C**	**B**	**S^n**	**P**	**Z**	**X^t**	**K^n**	**J**	**V**
**A**	**0.57**	0.00	0.11	0.01	0.06	0.05	0.03	0.02	0.05	0.09	0.01	0.01
**T**	0.00	**0.95**	0.00	0.03	0.00	0.00	0.00	0.01	0.00	0.00	0.01	0.01
**G**	0.10	0.00	**0.52**	0.00	0.01	0.04	0.10	0.00	0.01	0.11	0.00	0.06
**C**	0.01	0.03	0.00	**0.59**	0.03	0.00	0.00	0.09	0.02	0.01	0.19	0.00
**B**	0.07	0.00	0.02	0.04	**0.63**	0.02	0.00	0.03	0.10	0.07	0.04	0.00
**S^n**	0.05	0.00	0.04	0.00	0.02	**0.66**	0.06	0.00	0.04	0.03	0.00	0.10
**P**	0.03	0.00	0.11	0.00	0.00	0.06	**0.63**	0.00	0.02	0.05	0.00	0.10
**Z**	0.02	0.01	0.00	0.11	0.03	0.00	0.00	**0.72**	0.01	0.01	0.12	0.00
**X^t**	0.05	0.00	0.02	0.02	0.10	0.04	0.02	0.01	**0.66**	0.07	0.01	0.01
**K^n**	0.08	0.00	0.12	0.01	0.07	0.03	0.04	0.01	0.06	**0.53**	0.01	0.02
**J**	0.00	0.01	0.00	0.19	0.03	0.00	0.00	0.11	0.01	0.01	**0.61**	0.00
**V**	0.01	0.01	0.06	0.00	0.00	0.10	0.11	0.00	0.01	0.03	0.00	**0.69**

Supplementary Table 31. Nanopore datasets of supernumerary DNA uploaded to Sequence Reads Archive. Accession numbers for samples deposited in the SRA. Raw FAST5 and FASTQ basecalls (guppy) are provided. Model building datasets used in this work, alongside independent replicates, are available as testing data for the public. All samples can be found under SRA Bioproject PRJNA932328 [https://www.ncbi.nlm.nih.gov/bioproject/PRJNA932328] – Nanopore sequencing of 12-letter DNA (ATGCBSPZXKJV).

Sample Name	Contents	Sample Accession
PZ_libv2	HP-NNN-PZ	SAMN33188871
PZ_libv2-2	HP-NNN-PZ-2	SAMN33188872
BSn_libv2	HP-NNN-BS^n	SAMN33188873
BSn_libv2-2	HP-NNN-BS^n-2	SAMN33188874
BSc_libv2	HP-NNN-BS^c	SAMN33188875
BSc_libv2-2	HP-NNN-BS^c-2	SAMN33188876
JV_libv2	HP-NNN-JV	SAMN33188877
JV_libv2-2	HP-NNN-JV-2	SAMN33188878
XK_libv2	HP-NNN-XK	SAMN33188879
XK_libv2-2	HP-NNN-XK-2	SAMN33188880
blunt_libv2	HP-NNN-blunt	SAMN33188869
blunt_libv2-2	HP-NNN-blunt-2	SAMN33188870
val20-lib	val20-lib	SAMN33188881
val20-lib_BScXK	val20-lib_BScXK	SAMN33188882
S^{c}uper-12	12letter_setA	SAMN34367581
S^{n}uper-12	12letter_setB	SAMN34367582
PZ_XPCR	PCR_PZ	SAMN37609009

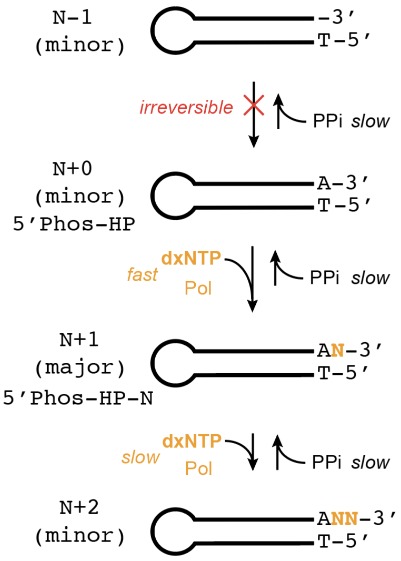

Supplementary Figure 1. Overview of non-templated N+1 tailing reaction. Tailing of blunt-end hairpin DNA substrates (N) will ideally lead to complete formation of XNA-tailed hairpin products (N+1 major). PPi release from tailing leads to slow background rate of pyrophosphorolysis, which acts in the reverse direction of nucleotide tailing (3′-exo). Pyrophosphorolysis is mitigated by adding YiPP to tailing reactions and balancing reaction duration and reaction rates. The over tailing of products to generate (N+2) hairpins is also considered in optimization for tailing reactions. N+1 tailing is generally thought to occur at a first-order reaction rate, 2 orders of magnitude slower than templated polymerization.[52] Furthermore, N+2 addition rates are polymerase specific and are thought to occur at first order rates 2 orders of magnitude slower than N+1 product formation.[53] End abbreviations: 3′ indicates 3′-OH, 5′- indicates 5′-PO_4.

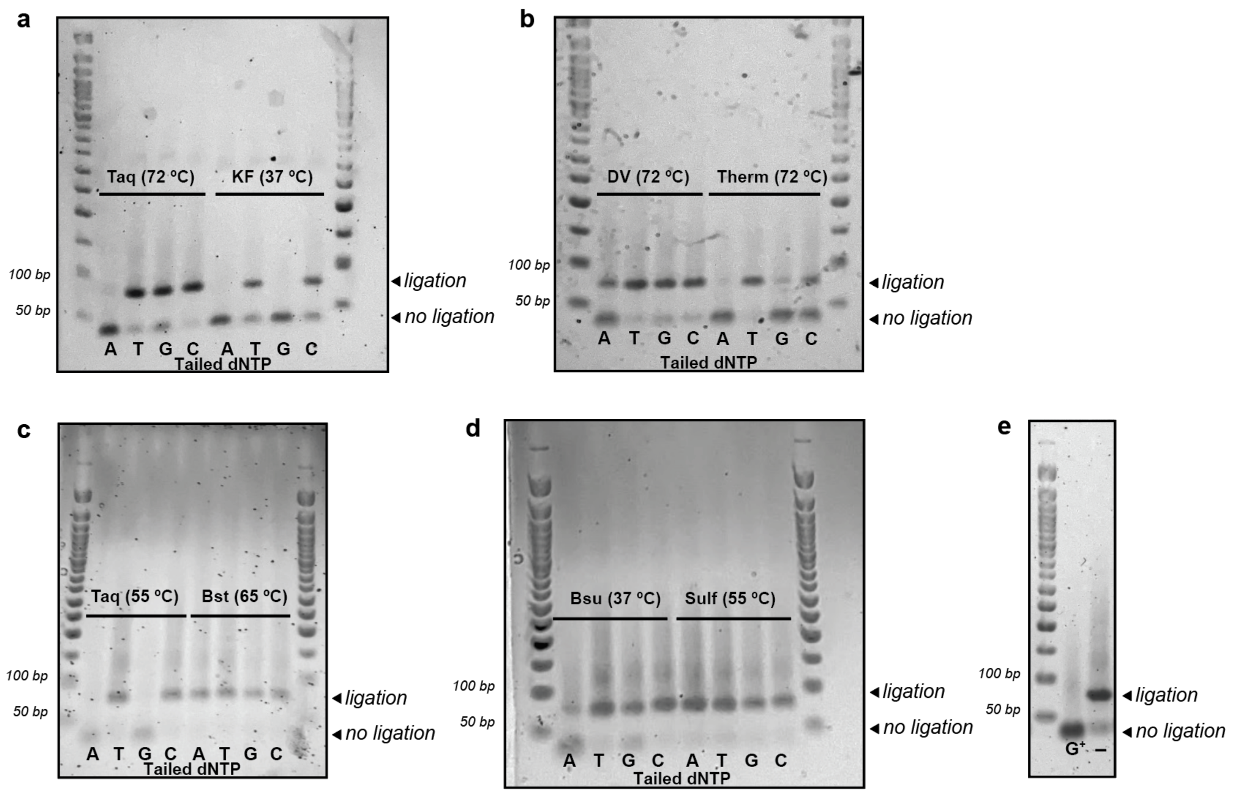

Supplementary Figure 2. Screening polymerases capable of effective tailing of both purine and pyrimidine dNTPs for canonical bases (N = A, T, G, C) by T4 ligation assay. A 5′-phosphorylated hairpin oligo with a 3′-blunt end was purchased from IDT (5′Phos-15HP; **Supplementary Table 2).** Oligos are first refolded by incubating 20 μM of oligo in a 100 mM NaCl, 10 mM Tris-HCl buffer (pH 8.2) at 90 °C for 3 minutes then cooling at 0.1 °C/s until reaching 20 °C. All subsequent tailing reactions used 16 μM 5′Phos-15HP (blunt-end with 15 nt in the hairpin region), 1.19 mM dNTP (with dNTP used specified on lane figure panel), and tailed for 1 h at the specified temperature using the specified polymerases. Subsequent T4 ligation reactions were performed with 11.2 μM of oligo for 1 h using T4 DNA Ligase Reaction Buffer which contains 1 mM ATP. (**a**) Tailing screen for Taq polymerase (0.25 U/μL, 72 °C) and Klenow Fragment (exo-; KF) polymerase (0.68 U/μL, 37 °C) followed by high concentration T4 ligation. (**b**) Tailing screen for Deep Vent (exo-; DV) polymerase (0.1 U/μL, 72 °C) and Therminator (Therm) polymerase (0.1 U/μL, 72 °C) followed by high concentration T4 ligation. (**c**) Tailing screen for Taq polymerase (0.25 U/μL, 55 °C) and Bst polymerase (0.4 U/μL, 65 °C) followed by T4 ligation. (**d**) Tailing screen for Bsu polymerase (0.25 U/μL, 37 °C) and *Sulfolobus* (Sulf) polymerase (0.1 U/μL, 55 °C) followed by T4 ligation. (**e**) Positive control (G^{+}) shows no ligation for a hairpin with a 3′ single nucleotide overhang (5′Phos-HP-3′G, lower band) while negative control (-) shows full ligation of blunt-end hairpin (upper band). Polymerase screening gels are representative of a single experimental replicate.

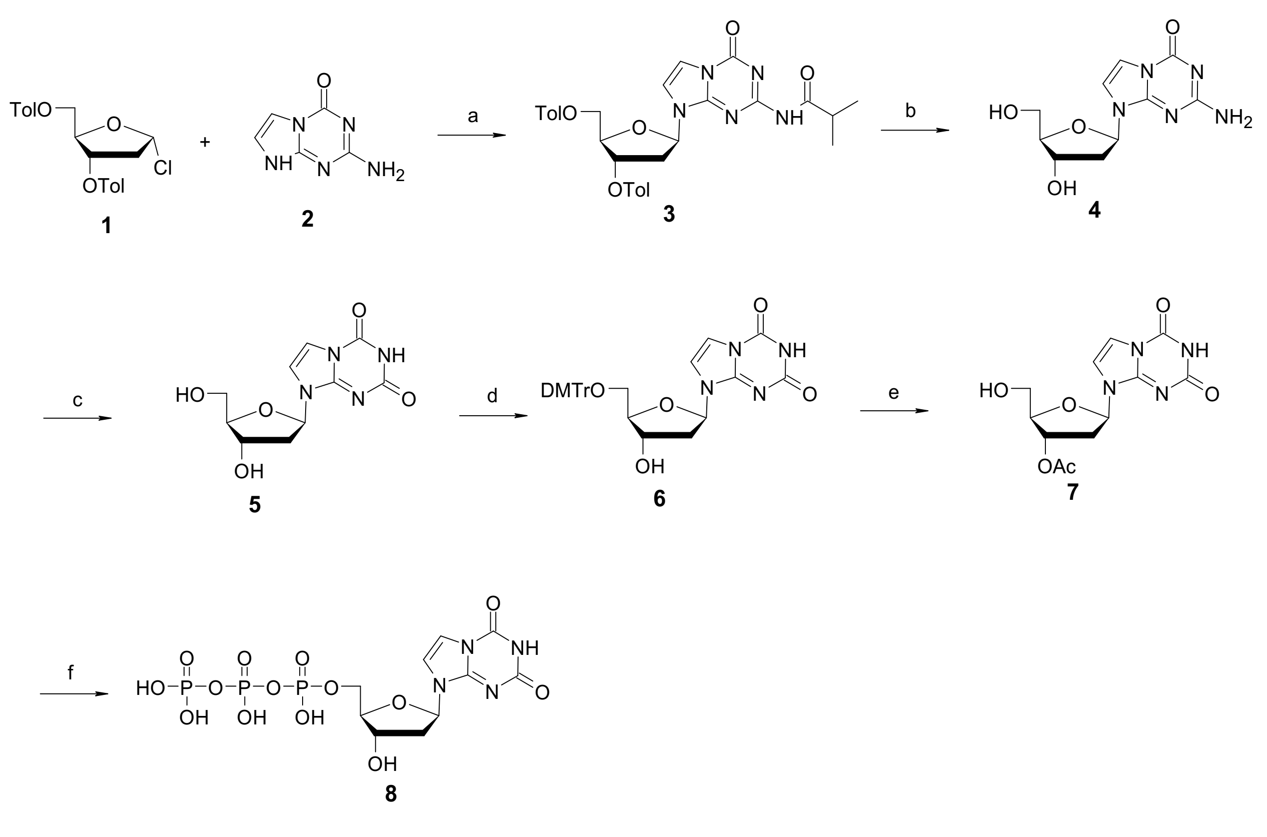

Supplementary Figure 3. Synthesis of dX^t triphosphate. Reagent and conditions: (a) (i) 10% aq. K_2CO_3, Bu_4NHSO_4, CH_2Cl_2, rt, 1 h, (ii) isobutyryl chloride, DMAP, pyridine, rt, 20 h, 19%; (b) $MeNH_2$, MeOH, rt, 2 days, 90%; (c) $NaNO_2/H_2O$, AcOH, rt, 2 days, 67%; (d) DMTCl, pyridine, rt, 20 h, 58%; (e) (i) Ac_2O, pyridine, rt, 18 h, (ii) 3% dichloroacetic acid in CH_2Cl_2, rt, 2 h, 79%; (f) (i) 2-chloro-4*H*-1,3,2-benzodioxaphosphorin-4-one, pyridine, dioxane, rt, 20 min, (ii) 0.2 M tributylammonium pyrophosphate, Bu_3N, rt, 20 min, (iii) I_2/H_2O/pyridine, rt, 30 min, (iv) NH_4OH, rt, 3 h. This synthetic scheme follows that of the originally reported synthesis.[54]

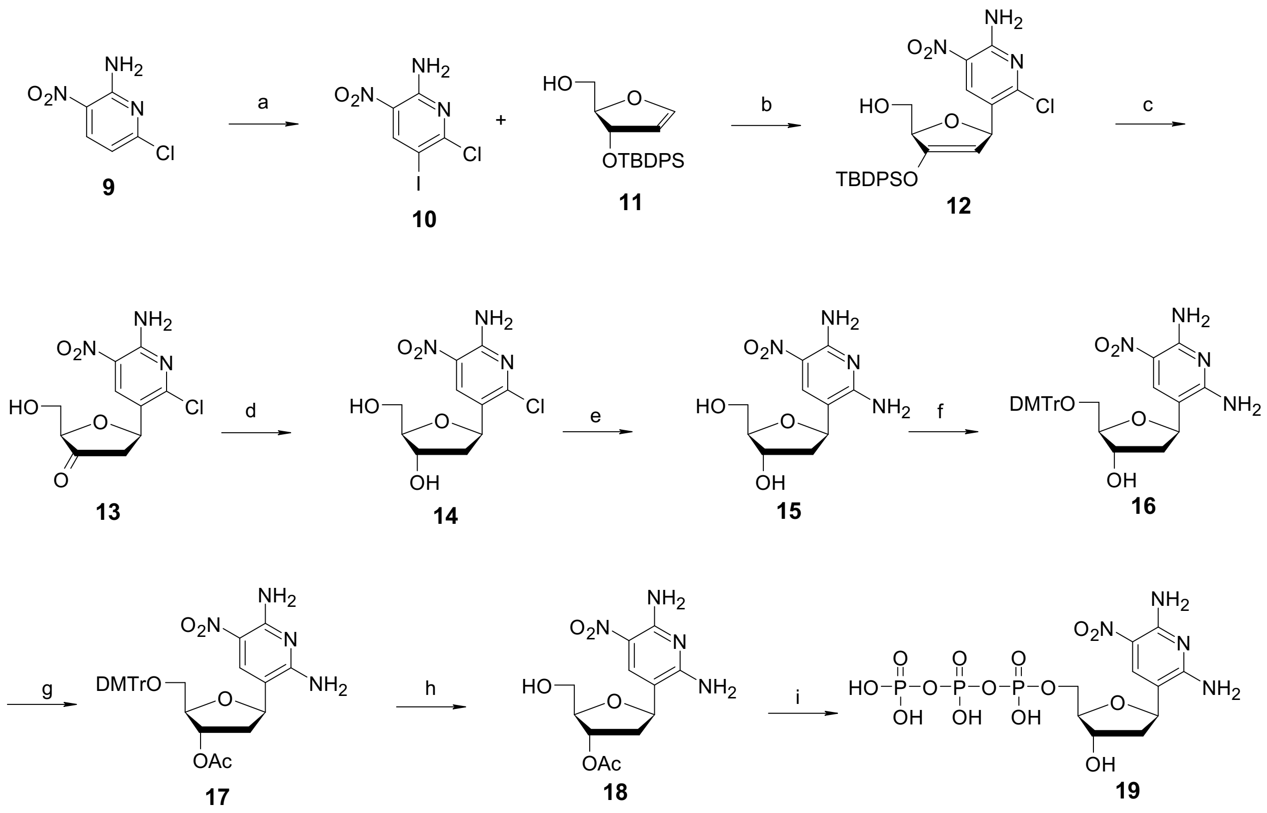

Supplementary Figure 4. Synthesis of dK^n triphosphate. Reagents and conditions: (a) I_2/H_5IO_6, $AcOH/H_2SO_4/H_2O$, 95 °C, 1 h, 88%; (b) $Pd(OAc)_2$, $AsPh_3$, Ag_2CO_3, $CHCl_3$, reflux, 20 h, 63%; (c) TBAF, THF/AcOH, 0 °C, 30 min; (d) $NaBH(OAc)_3$, MeCN/AcOH, 0 °C, 1 h, 80%, 2 steps; (e) 7 N NH_3 in MeOH, 110 °C, 20 h, 90%; (f) DMTCl, DMAP, pyridine, rt, 4 h, 62%; (g) Ac_2O, DMAP, pyridine, rt, 2 h, 93%; (h) 3% dichloroacetic acid in CH_2Cl_2, rt, 1 h, 93%; (f) (i) 2-chloro-4*H*-1,3,2-benzodioxaphosphorin-4-one, pyridine, dioxane, rt, 20 min, (ii) 0.2 M tributylammonium pyrophosphate, Bu_3N, rt, 20 min, (iii) I_2/H_2O/pyridine, rt, 30 min, (iv) NH_4OH, rt, 4 h.

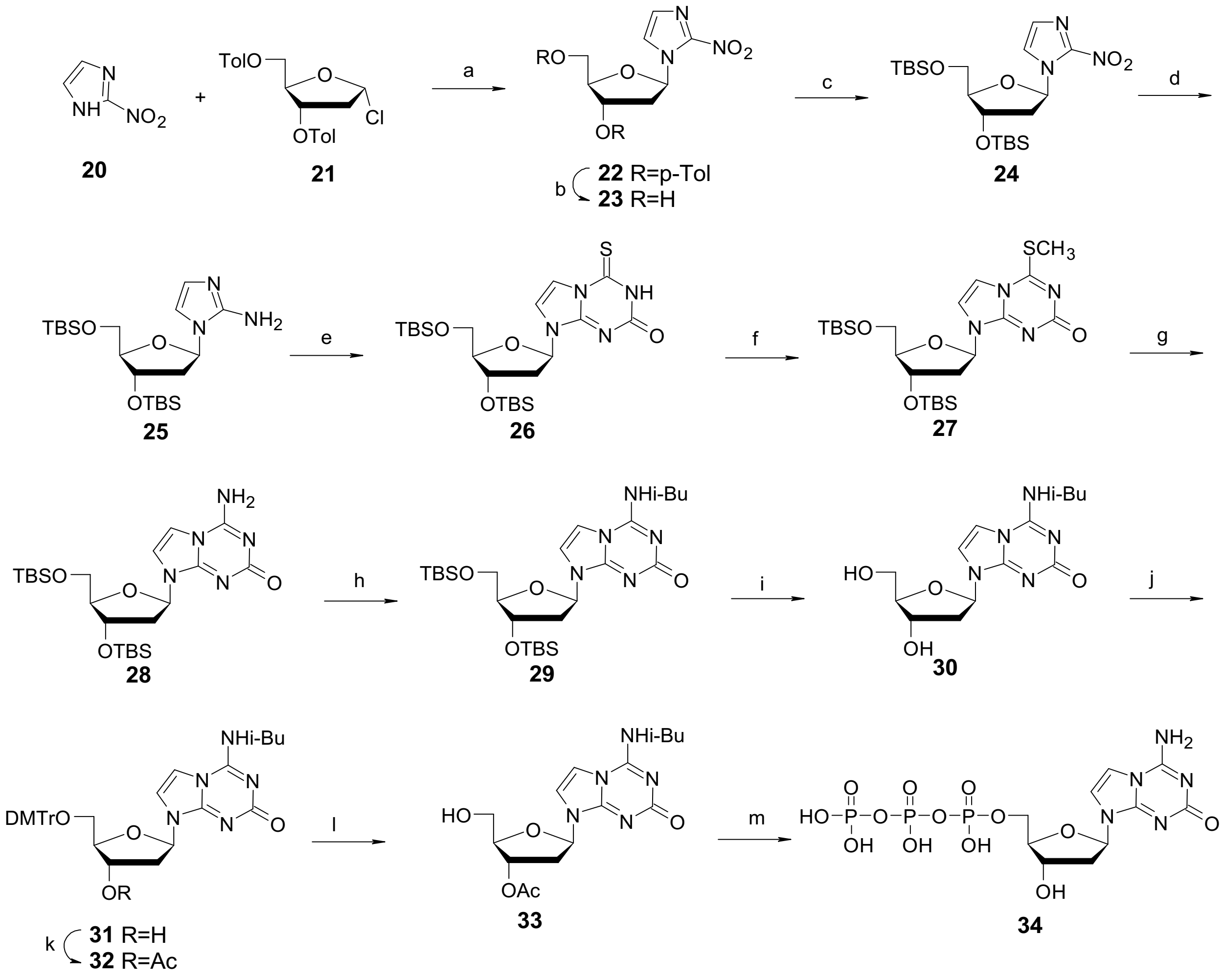

Supplementary Figure 5. Synthesis of dJ triphosphate. Reagents and Conditions: (a) (i) K_2CO_3, TDA-1, CH_3CN, rt; (b) 40% $MeNH_2$ in water, MeOH, 18 h, rt, 84%, 2 steps; (c) TBDMSCl, imidazole, DMF, rt, 18 h, 91%; (d) 10% Pd/C, EtOH, H_2, rt, 18 h, 85%; (e) Phenyl chloroformate, KSCN, 40 °C, 4 h, 32%; (f) CH_3I, $NaHCO_3$, 1,4-dioxane/MeOH, rt, 30 h, 67 %; (g) 7 N NH_3 in MeOH, rt, 40 h, 64%; (h) isobutyryl chloride, DMAP, pyridine, rt, 1 h, 85%; (i) HF in pyridine, THF, rt, 18 h, 84%; (j) DMTCl, Et_3N, CH_2Cl_2, rt, 18 h, 86%; (k) Ac_2O, pyridine, rt, 18 h, 98%; (l) 3% dichloroacetic acid in CH_2Cl_2, rt, 2 h, 82%; (f) (i) 2-chloro-4*H*-1,3,2-benzodioxaphosphorin-4-one, pyridine, dioxane, rt, 15 min, (ii) 0.2 M tributylammonium pyrophosphate, Bu_3N, rt, 20 min, (iii) I_2/H_2O/pyridine, rt, 20 min, (iv) NH_4OH, rt, 18 h.

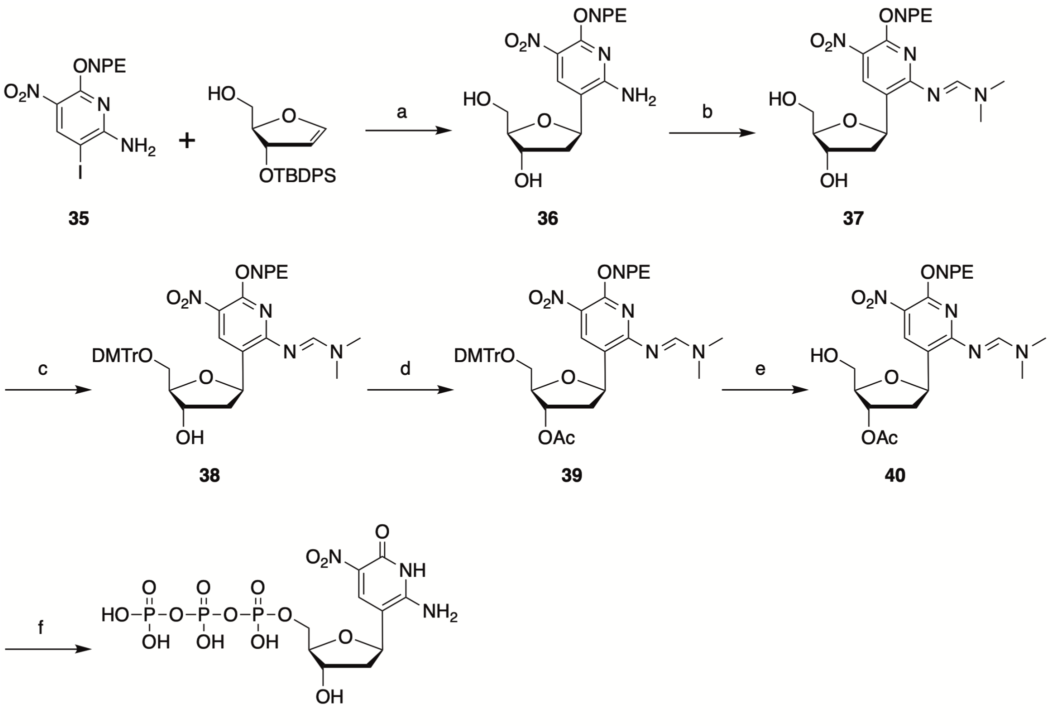

Supplementary Figure 6. Synthesis of dV triphosphate. Reagent and condition: a. 1. $Pd(OAc)_2$, $AsPh_3$, Ag_2CO_3, $CHCl_3$ 2. HF-pyridine, THF 3. $NaBH(OAc)_3$, AcOH, CH_3CN, 59% (for 3 steps) b. $Me_2NHCH(OMe)_2$, MeOH, 88% c. DMTr-Cl, TEA, DMAP, CH_2Cl_2, 91% d. Ac_2O, DMAP, pyridine, CH_2Cl_2, 88% e. dichloroacetic acid, CH_2Cl_2, 85% f. 2-chloro-1,3,2-benzodioxaphsphorin-4-one, pyridine, dioxane; tribuylammonium pyrophosphate, Bu_3N, DMF; I_2, pyridine; DBU, CH_3CN; NH_4OH, 74%.

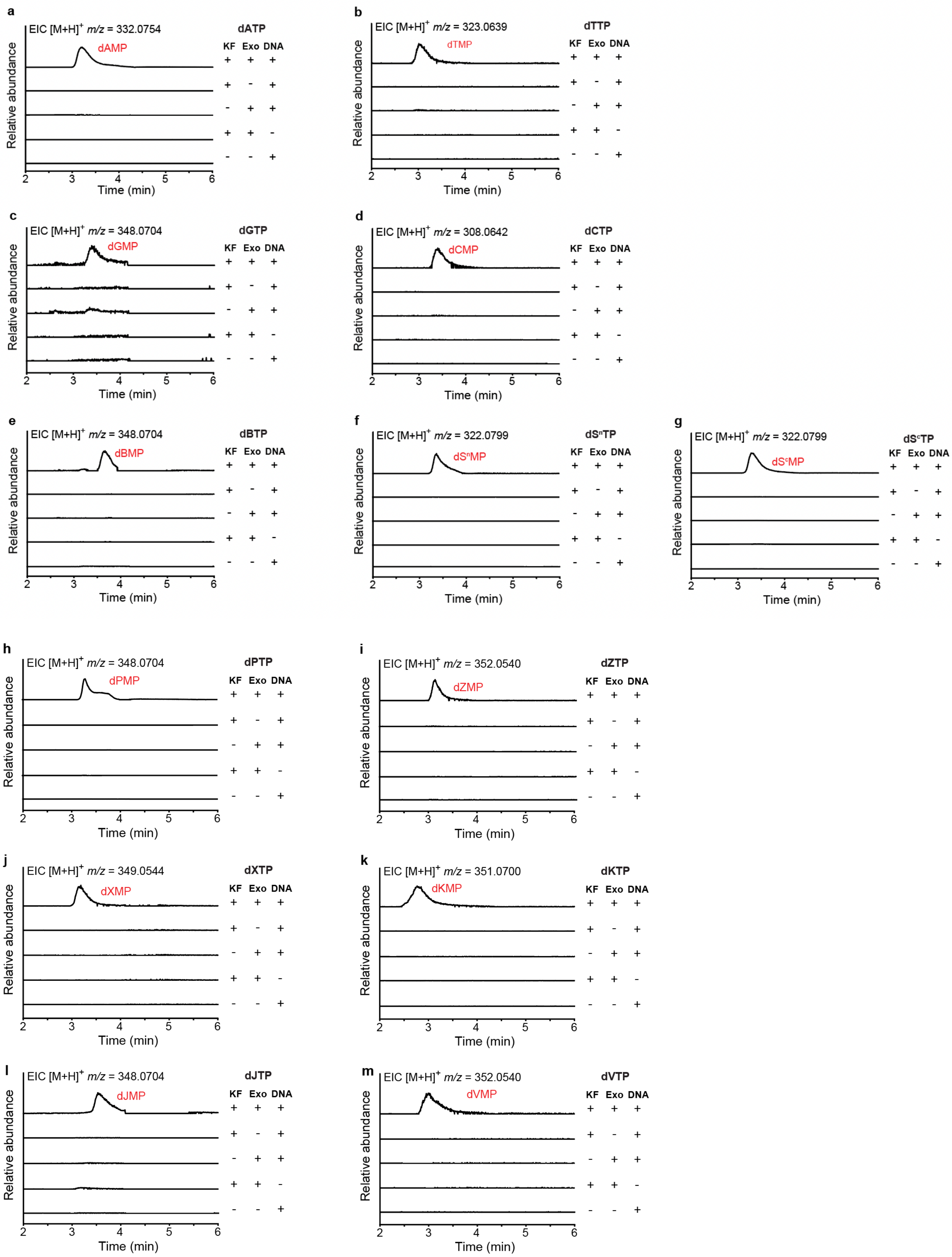
a
EIC [M+H]+ m/z = 332.0754
dATP
dAMP
b
EIC [M+H]+ m/z = 323.0639
dTTP
dTMP
c
EIC [M+H]+ m/z = 348.0704
dGTP
dGMP
d
EIC [M+H]+ m/z = 308.0642
dCTP
dCMP
e
EIC [M+H]+ m/z = 348.0704
dBTP
dBMP
f
EIC [M+H]+ m/z = 322.0799
dS^{n}TP
dS^{n}MP
g
EIC [M+H]+ m/z = 322.0799
dS^{c}TP
dS^{c}MP
h
EIC [M+H]+ m/z = 348.0704
dPTP
dPMP
i
EIC [M+H]+ m/z = 352.0540
dZTP
dZMP
j
EIC [M+H]+ m/z = 349.0544
dXTP
dXMP
k
EIC [M+H]+ m/z = 351.0700
dKTP
dKMP
l
EIC [M+H]+ m/z = 348.0704
dJTP
dJMP
m
EIC [M+H]+ m/z = 352.0540
dVTP
dVMP
KF Exo DNA
+ + +
+ - +
- + +
+ + -
- - +
Relative abundance
Time (min)
2 3 4 5 6

Supplementary Figure 7. UPLC/QTOF validation of tailing activity for all dNTPs and dxNTPs by Klenow Fragment (exo-). (**a**-**m**) Full set of controls for the data shown in **Fig. 2b**. Extracted ion chromatograms (EIC) show relative abundance of either dNMP or dxNMP release when corresponding dNTPs/dxNTPs are used as a substrate for polymerase (KF exo-) tailing. Chromatogram scales are normalized for comparison of runs within each panel. dNTP or dxNTP used in each reaction shown in panel legend. Reactions controlled for polymerase (+/- KF), Exo III (+/- Exo), or hairpin DNA (+/- DNA). Source data are provided as a Source Data file.

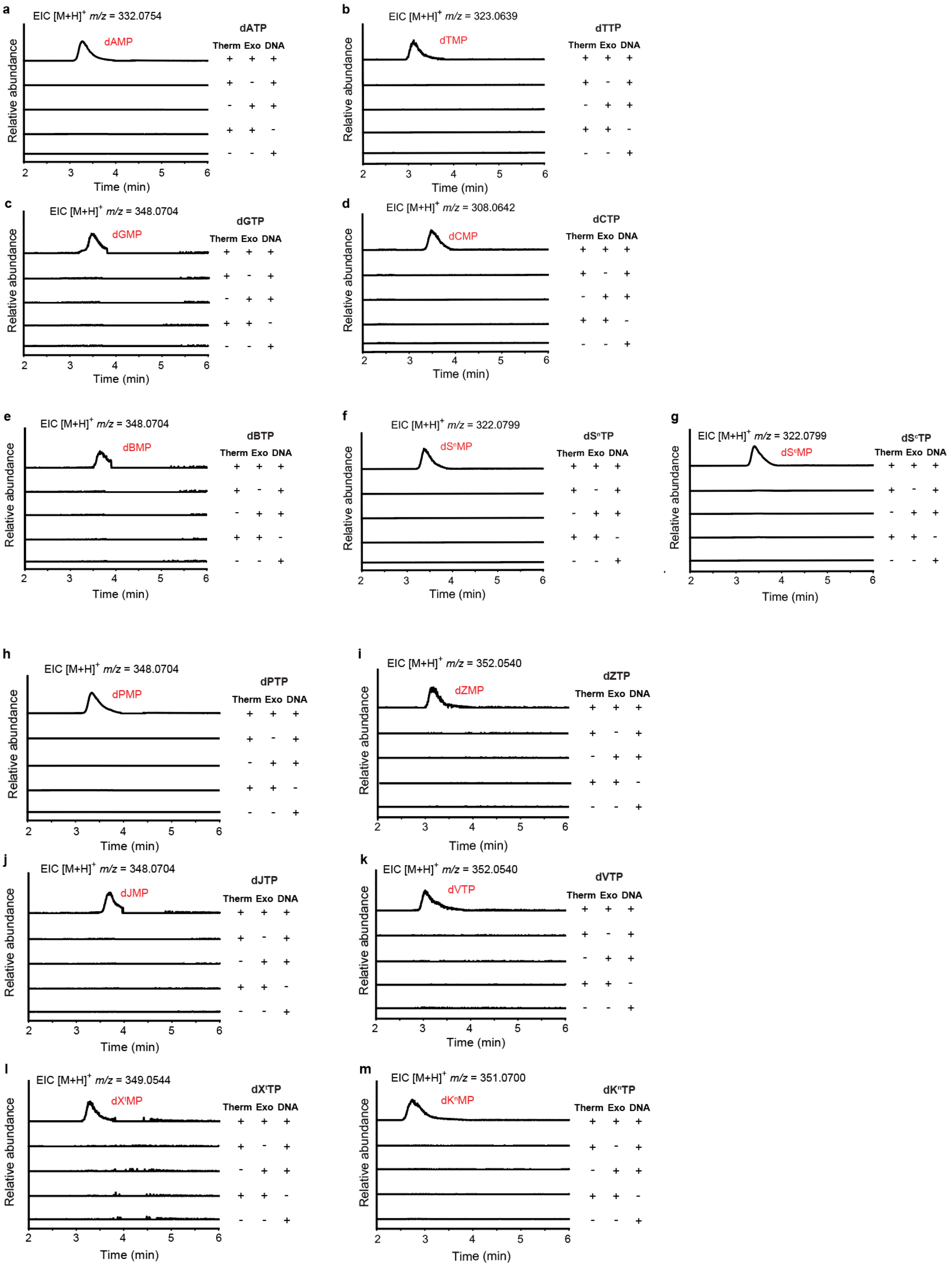
a
EIC [M+H]+ m/z = 332.0754
dATP
dAMP
b
EIC [M+H]+ m/z = 323.0639
dTTP
dTMP
c
EIC [M+H]+ m/z = 348.0704
dGTP
dGMP
d
EIC [M+H]+ m/z = 308.0642
dCTP
dCMP
e
EIC [M+H]+ m/z = 348.0704
dBTP
dBMP
f
EIC [M+H]+ m/z = 322.0799
dS^{n}TP
dS^{n}MP
g
EIC [M+H]+ m/z = 322.0799
dS^{c}TP
dS^{c}MP
h
EIC [M+H]+ m/z = 348.0704
dPTP
dPMP
i
EIC [M+H]+ m/z = 352.0540
dZTP
dZMP
j
EIC [M+H]+ m/z = 348.0704
dJTP
dJMP
k
EIC [M+H]+ m/z = 352.0540
dVTP
dVTP
l
EIC [M+H]+ m/z = 349.0544
dX^{t}TP
dX^{t}MP
m
EIC [M+H]+ m/z = 351.0700
dK^{n}TP
dK^{n}MP
Therm Exo DNA
Relative abundance
Time (min)

Supplementary Figure 8. UPLC/QTOF validation of tailing activity for all dNTPs and dxNTPs by Therminator. (**a**-**m**) Full set of controls for the data shown in **Fig. 2c**. Extracted ion chromatograms (EIC) show relative abundance of either dNMP or dxNMP release when corresponding dNTPs/dxNTPs are used as a substrate for polymerase (Therminator; Therm) tailing. Chromatogram scales are normalized for comparison of runs within each panel. dNTP or dxNTP used in each reaction shown in panel legend. Reactions controlled for polymerase (+/- Therm), Exo III (+/- Exo), or hairpin DNA (+/- DNA). Source data are provided as a Source Data file.

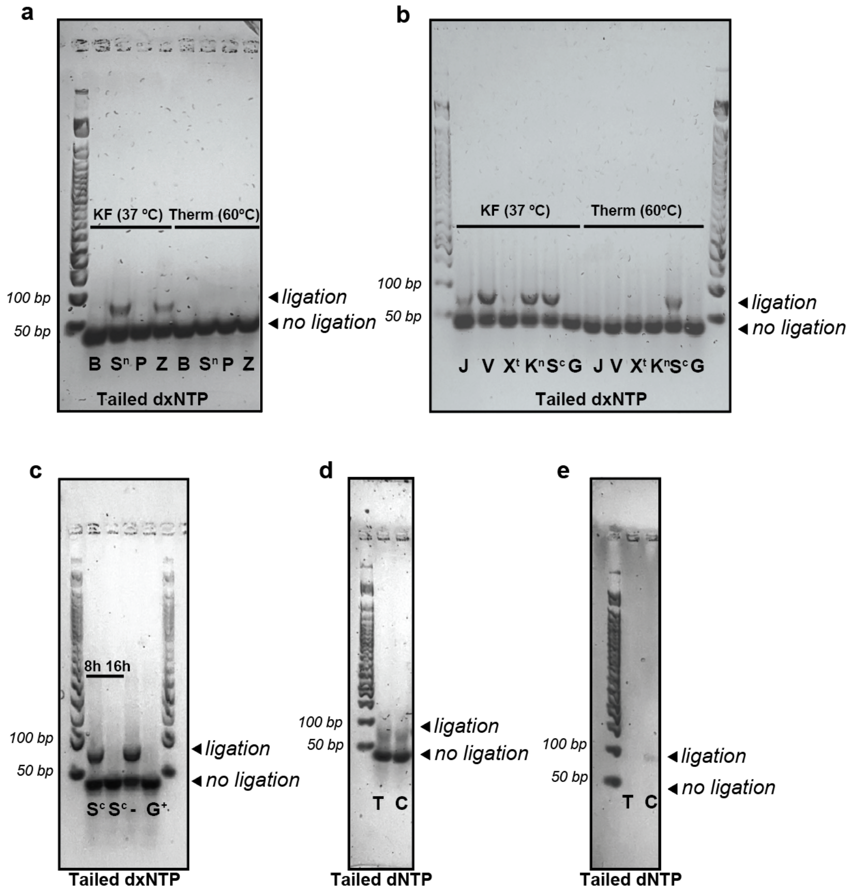

Supplementary Figure 9. Screening and optimization of XNA tailing conditions. All tailing reactions used 11.9 µM 5′Phos-11HP, 1.19 mM of specified dNTP/dxNTP, and tailed at the specified temperature for the specified times using either Klenow Fragment (KF exo-; 0.71 U/µL) or Therminator (Therm; 0.29 U/µL). Tailing completeness was measured via T4 ligation assays. Hairpins tailed with a dNTP or dxNTP result in a single nucleotide overhang which is no longer a substrate for self-ligation. No tailing results in blunt-ended hairpins which self ligate in the presence of T4 DNA ligase. (**a**) XNA tailing screen using KF exo- and Therm for 8 h. (**b**) XNA tailing screen using KF and Therm for 8 h. (**c**) Additional S^c tailing screen using Therm for 8 or 16 h. Positive control (G$^+$) shows no ligation for a hairpin with a 3′ single nucleotide overhang (5′Phos-HP-3′G, lower band) while negative control (-) shows ligation of blunt-end hairpin (upper band). (**d**) Tailing screen for dTTP and dCTP using Therm at 60 °C for 4 h. (**e**) Tailing screen for dTTP and dCTP using Therm at 60°C for 4 h followed by T4 DNA ligation and digestion with exonucleases at 37 °C for 1 h using Exo I (2.7 U/µL), Exo III (13.3 U/µL) and Exo VIII (truncated, 0.67 U/µL). Screening gels are representative of a single experimental replicate.

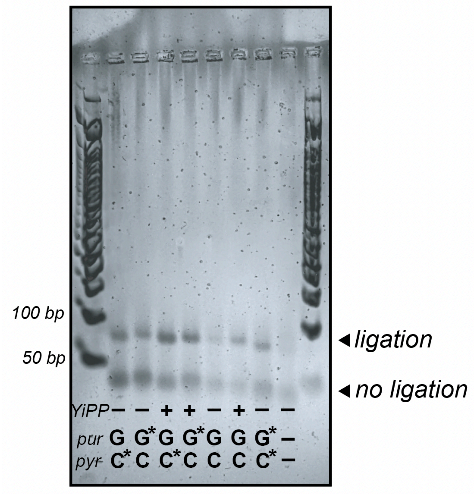

Supplementary Figure 10. Addition of yeast inorganic pyrophosphatase (YiPP) leads to slight improvements in XNA tailing reaction yield. 5′-phosphorylated hairpin oligos with either a 3′-blunt end or 3′-single nucleotide (-G, or -C) overhangs were purchased from IDT (5′-Phos-11HP; **Supplementary Table 2).** Separately, 11.4 µM of 3′-blunt end oligos were tailed with 1.14 mM of dCTP or dGTP, Klenow Fragment (exo-; KF; 0.68 U/µL), and either 0.009 U/µL of YiPP or no YiPP at 37 °C for 4 h. Subsequent ligation reactions were performed using 2.6 µM of two oligos with complementary overhang bases, either enzymatically tailed (G, C) or synthesized overhangs (G*, C*). Ligation reactions were incubated for 15 min at 16 °C using T7 DNA ligase (272 U/µL) and carried out in 1X of NEB StickTogether™ buffer which contains 7.5% (w/v) PEG 6000. Blunt-end hairpins (-/-) serve as a negative ligation control as the short reaction time prevents blunt end ligation. Unligated materials were digested using exonuclease I (2.7 U/µL), exonuclease III (13.3 U/µL) and exonuclease VII (1.33 U/µL) for 1 h at 37°C. Exonuclease reactions were heat inactivated by incubation at 95 °C for 10 min and then at 80 °C for 10 min. Note that in this set of experiments, Exo VII was used which has a higher heat inactivation temperature than Exo VIII (truncated) used in other aspects of this work. We also found Exo VII would result in incomplete digestion (lower band) and required different buffer conditions. In subsequent screening work, Exo VIII (truncated) was used instead in the exonuclease treatment steps. Positive control with G* and C* shows ligation of hairpins with G and C synthetic overhangs. Gel representative of a single experimental replicate.

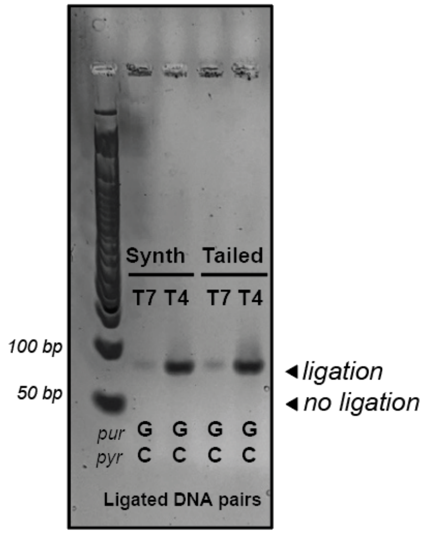

Supplementary Figure 11. Enzymatic tailing does not lead to measurable differences in ligation when compared to ligation using fully synthetic hairpin with N+1 tails. Ligation of over-tailed product (i.e., more than one nucleotide added to the blunt 3′-end) with an N+1 tailed hairpin would result in dsDNA that contains a gap of one or more nucleotides. The gap region exposes a 3′ and 5′ end that would make this product susceptible to exonuclease degradation. Therefore, one way we tested to see if over-tailing was a problem was to compare how much ligated product was observed (as measured by agarose gel band intensity) if hairpins were tailed enzymatically vs made synthetically. Here, 5′-phosphorylated hairpin oligos with either a 3′-blunt end or 3′-single nucleotide (-G, or -C) overhangs were purchased from IDT (**Supplementary Table 2).** Oligos were first folded using previously described methods. Blunt end oligo 5′Phos-11HP was then tailed with dCTP using conditions listed in **Supplementary Table 8**. Subsequent ligation reactions were performed using T7 or T4 DNA ligase. Either the dCTP-tailed oligo (Tailed) or 5′Phos-HP-3′C (Synth) was ligated to 5′Phos-HP-3′G. For T7 ligation reactions, 2.7 μM of each oligo were incubated with 272 U/μL of T7 DNA ligase and StickTogether™ DNA ligase buffer at 16 °C for 15 min, after which the ligase was heat inactivated at 65 °C for 10 min. For T4 ligation reactions, 4.2 μM of each oligo were incubated with 80 U/μL of T4 DNA ligase and T4 DNA ligase buffer at 16 °C for 2 h, after which the ligase was heat inactivated at 65 °C for 10 min. Unreacted hairpins or incomplete ligation products were removed by exonuclease treatment performed at 37 °C for 1 h using Exo I (2.7 U/μL), Exo III (13.3 U/μL), and Exo VIII (truncated, 0.67 U/μL). Exonuclease reactions were heat inactivated by incubation at 80 °C for 20 min. Results suggest over-tailing, if present, is not significant under tested conditions. Gel representative of a single experimental replicate.

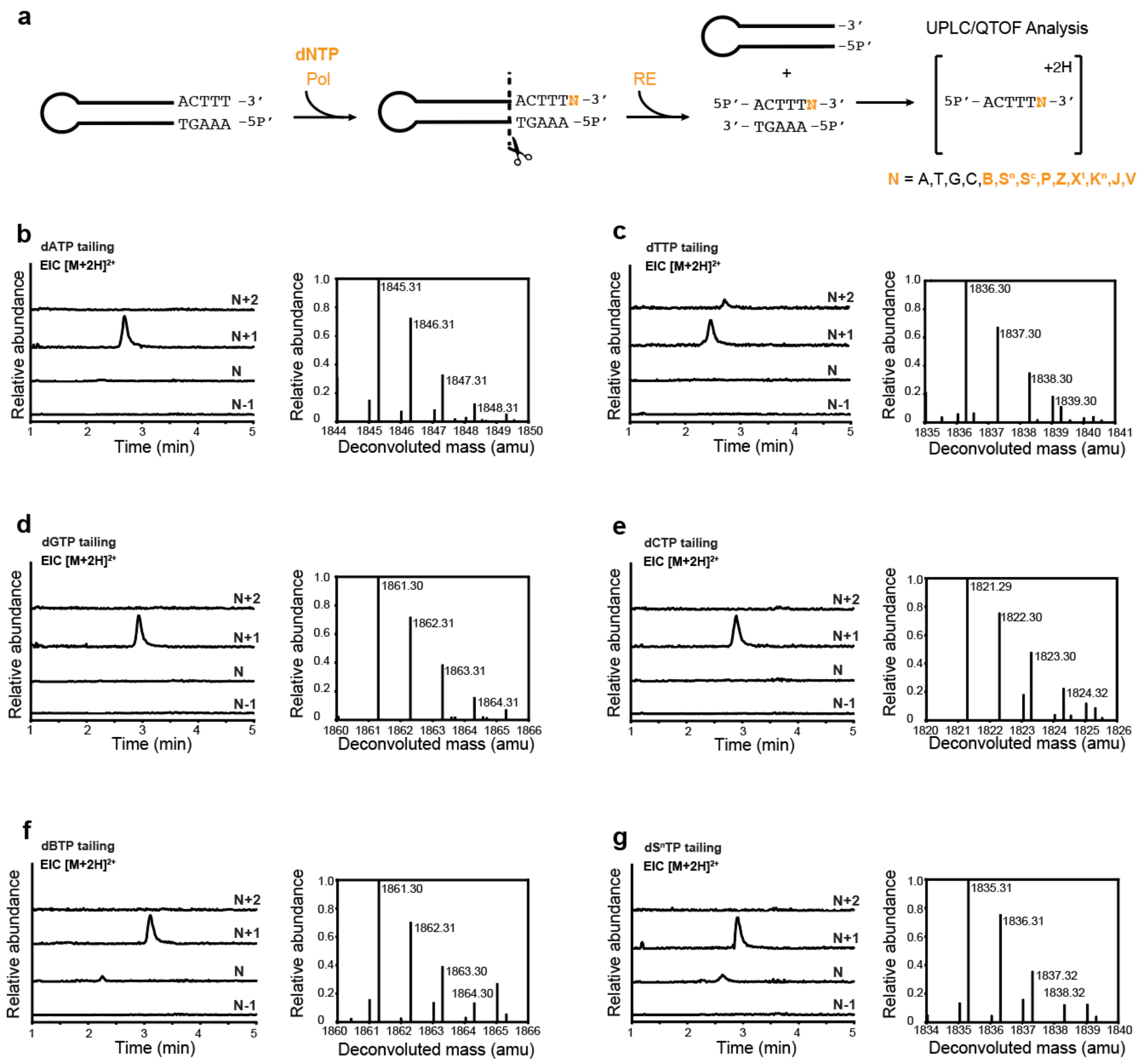
a
dNTP
Pol
RE
UPLC/QTOF Analysis
ACTTT -3'
TGAAA -5P'
5P' - ACTTTN -3'
3' - TGAAA -5P'
+2H
N = A,T,G,C,B,Sⁿ,Sᶜ,P,Z,Xᵗ,Kⁿ,J,V
b
dATP tailing
EIC [M+2H]2+
c
dTTP tailing
d
dGTP tailing
e
dCTP tailing
f
dBTP tailing
g
dSⁿTP tailing
N+2
N+1
N
N-1
Relative abundance
Time (min)
Deconvoluted mass (amu)
1845.31
1846.31
1847.31
1848.31
1836.30
1837.30
1838.30
1839.30
1861.30
1862.31
1863.31
1864.31
1821.29
1822.30
1823.30
1824.32
1861.30
1862.31
1863.30
1864.30
1835.31
1836.31
1837.32
1838.32

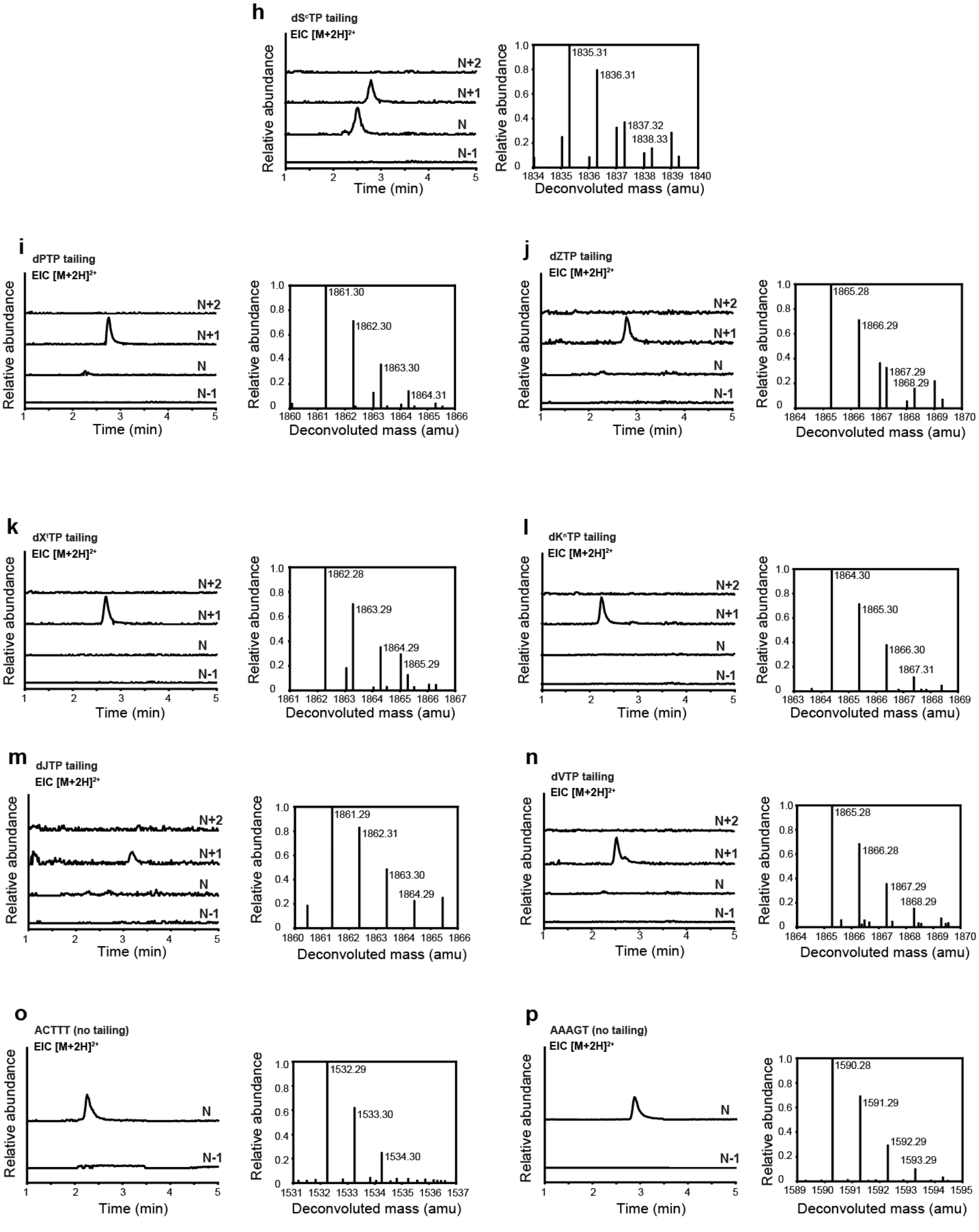

h
dS^cTP tailing
EIC [M+2H]2+
Relative abundance
N+2
N+1
N
N-1
Time (min)
1835.31
1836.31
1837.32
1838.33
Deconvoluted mass (amu)
i
dPTP tailing
EIC [M+2H]2+
1861.30
1862.30
1863.30
1864.31
j
dZTP tailing
EIC [M+2H]2+
1865.28
1866.29
1867.29
1868.29
k
dX^tTP tailing
EIC [M+2H]2+
1862.28
1863.29
1864.29
1865.29
l
dK^nTP tailing
EIC [M+2H]2+
1864.30
1865.30
1866.30
1867.31
m
dJTP tailing
EIC [M+2H]2+
1861.29
1862.31
1863.30
1864.29
n
dVTP tailing
EIC [M+2H]2+
1865.28
1866.28
1867.29
1868.29
o
ACTTT (no tailing)
EIC [M+2H]2+
1532.29
1533.30
1534.30
p
AAAGT (no tailing)
EIC [M+2H]2+
1590.28
1591.29
1592.29
1593.29

q

EIC [M+2H]$^{2+}$

N-1	m/z	N	m/z
Sense	615.1111	Sense	767.1341
Antisense	639.6200	Antisense	796.1488

EIC [M+2H]$^{2+}$

N+1	m/z	N+2	m/z
+A	923.6629	+AA	1080.1917
+T	919.1571	+TT	1071.1801
+G	931.6603	+GG	1096.1866
+C	911.6572	+CC	1056.1804
+B	931.6603	+BB	1096.1866
+S^n	918.6651	+S^nS^n	1070.1961
+S^c	918.6651	+S^cS^c	1070.1961
+P	931.6603	+PP	1096.1866
+Z	933.6519	+ZZ	1100.1697
+X^t	932.1523	+X^tX^t	1097.1706
+K^n	933.1599	+K^nK^n	1099.1857
+J	931.6603	+JJ	1096.1866
+V	933.6519	+VV	1100.1697

Supplementary Figure 12. High resolution LC/MS of oligo showing N+1 tailing as major product. (**a**) Hairpin oligo, 5′Phos-ScaI-HP (**Supplementary Table 2**) was tailed using optimized conditions with all dNTPs and dxNTPs described in this work. After tailing, ScaI digestion was used to cleave the 3′-end, generating a short oligo that could be directly detected by LC/MS. (**b-n**) Extracted ion chromatograms (EIC) showing formation of N+1 tailed product (6 nt oligo) for all dNTPs and dxNTPs. In each chromatogram set, EIC for starting material (N), pyrophosphorolysis (N-1), and processive tailing (N+2) are also shown. Deconvoluted mass spectra of N+1 product is shown to resolve isotopes. (**o-p**) EIC and deconvoluted spectra show negative control reactions (ScaI-treated starting material, sense and antisense strands). (**q**) Exact masses calculated for EIC are tabulated. End abbreviations: 3′ indicates 3′-OH, 5P′- indicates 5′-PO_4. Source data are provided as a Source Data file.

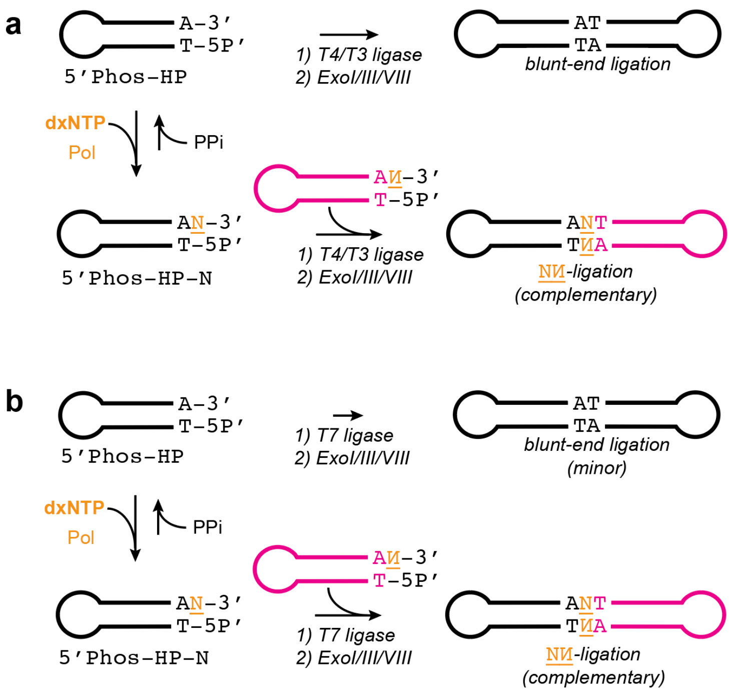

Supplementary Figure 13. Overview of T3 DNA ligase, T4 DNA ligase, and T7 DNA ligase products. (**a**) Major products formed from T3 ligation and T4 ligation assays between hairpins generated in this work. (**b**) Major and minor products formed for T7 ligation assays in this work. T7 ligase preferentially ligates hairpins with a cohesive nucleotide overhang and has minimal blunt-end ligation activity.[55] In reaction conditions with crowding agents such as high MW PEG, T7 ligase has been observed to perform blunt end ligation though to a lesser extent than T3 ligase and T4 ligase. Full hairpin sequences used in this work can be found in **Supplementary Table 2**. Nucleic acid end abbreviation: 3′ indicates 3′-OH, 5P′- indicates 5′-PO_4.

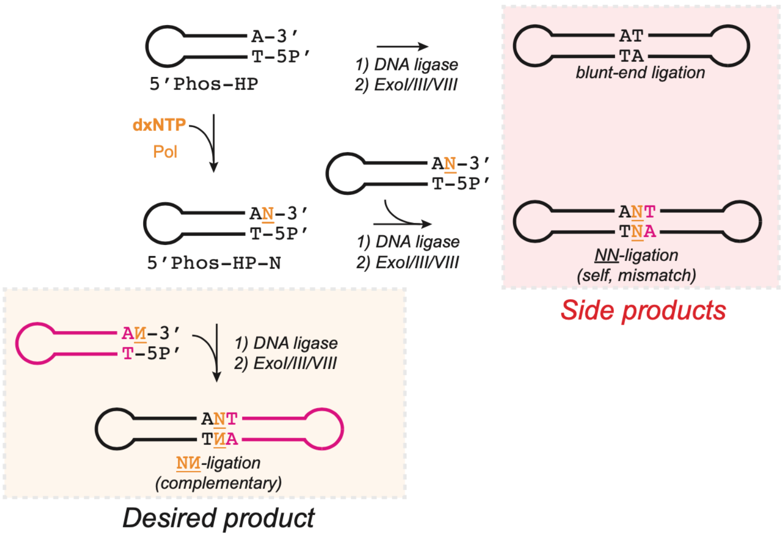

Supplementary Figure 14. Overview of XNA ligation products from XNA tailed hairpins. XNA ligation reactions were optimized making the following considerations of possible side products. Starting material is thought to be tailed by XNA tailing to > 95% completion. Untailed starting material (blunt-end hairpin DNA) can self-ligate forming blunt-ended dsDNA side product. XNA tailed DNA can also self-ligate in the presence of promiscuous DNA ligases in a mismatch configuration (e.g. P:P ligation). Desired product should only form in reactions that contain two hairpins tailed with complementary XNA bases. Nucleic acid end abbreviation: 3′ indicates 3′-OH, 5P′- indicates 5′-PO_4.

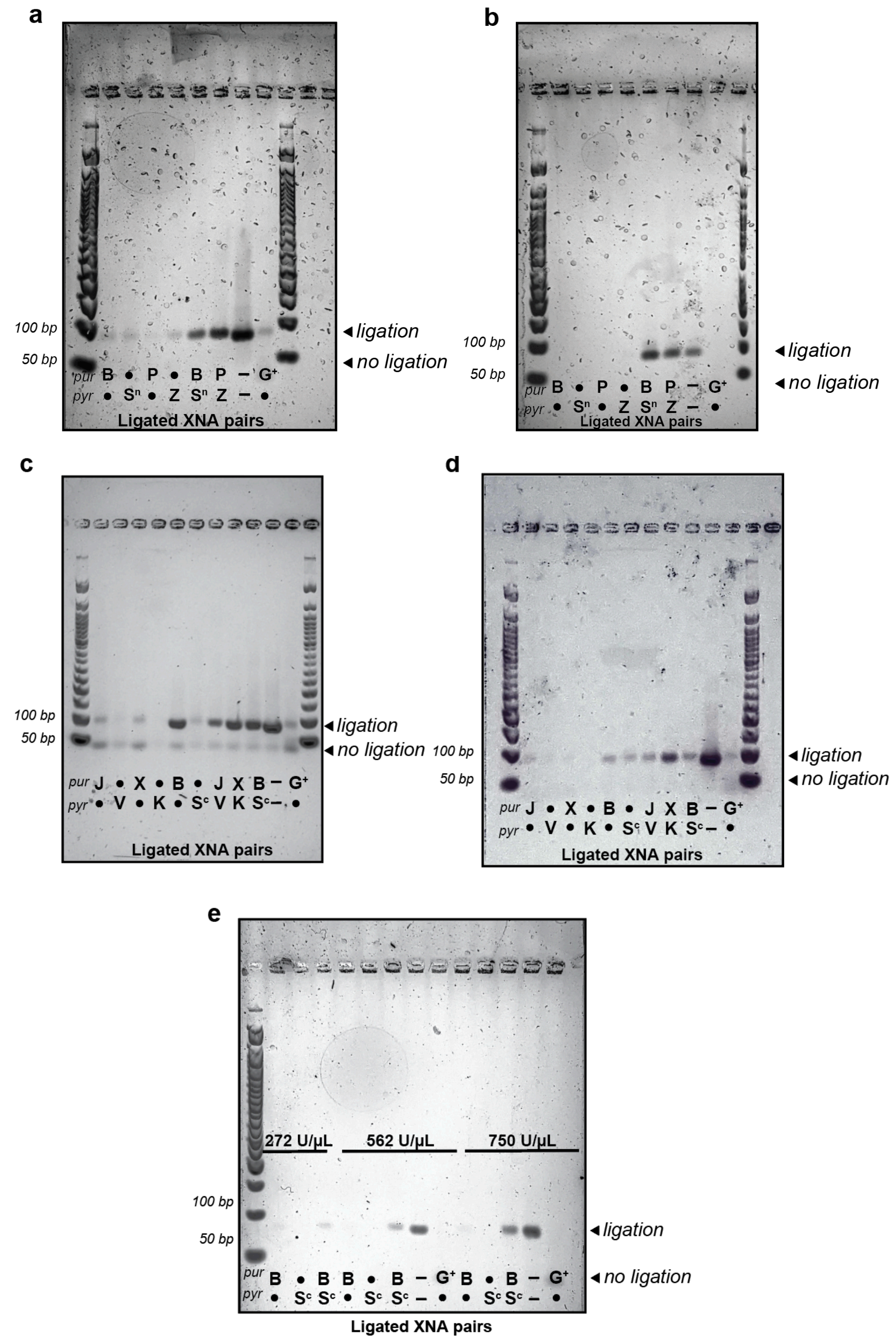

Supplementary Figure 15. Screening and optimization of ligation conditions across all XNA bases. All tailing reactions used conditions listed in **Supplementary Table 8** unless otherwise specified. Subsequent ligation reactions were performed using 4.7 µM of one oligo or 2.4 µM of two oligos with complementary tailed bases. Ligation reactions were incubated for 16 h at 16 °C using the specified ligase

and carried out in 1X of NEB StickTogether™ buffer which contains 7.5% (w/v) PEG 6000. Improperly ligated materials were digested using Exo I (1.5 U/μL), Exo III (7.7 U/μL) and Exo VIII (truncated, 0.77 U/μL) for 1 h at 37 °C. Exonuclease reactions were heat inactivated by incubation at 80 °C for 20 min. (**a**) T4 ligase (36 U/μL) assay. (**b**) T7 ligase (272 U/μL) assay. (**c**) T3 ligase assay (272 U/μL), with 8 h tailing for S^c. (**d**) T4 ligase assay (36 U/μL) containing 0.4 M betaine with 8 h tailing for S^c. (**e**) BS^c ligation with differing amounts of T7 ligase. In all gels, • indicates absence of the hairpin tailed with the complementary base. positive control for ligation (–/–) shows full ligation of blunt-end hairpin (upper band, no degradation by exonucleases), while negative control (G^+/•) shows either mismatch ligation or lack of ligation and subsequent digestion for a hairpin with a 3′ single nucleotide overhang (5′Phos-HP-3′G). Gels **c-d** are representative of two experimental replicates.

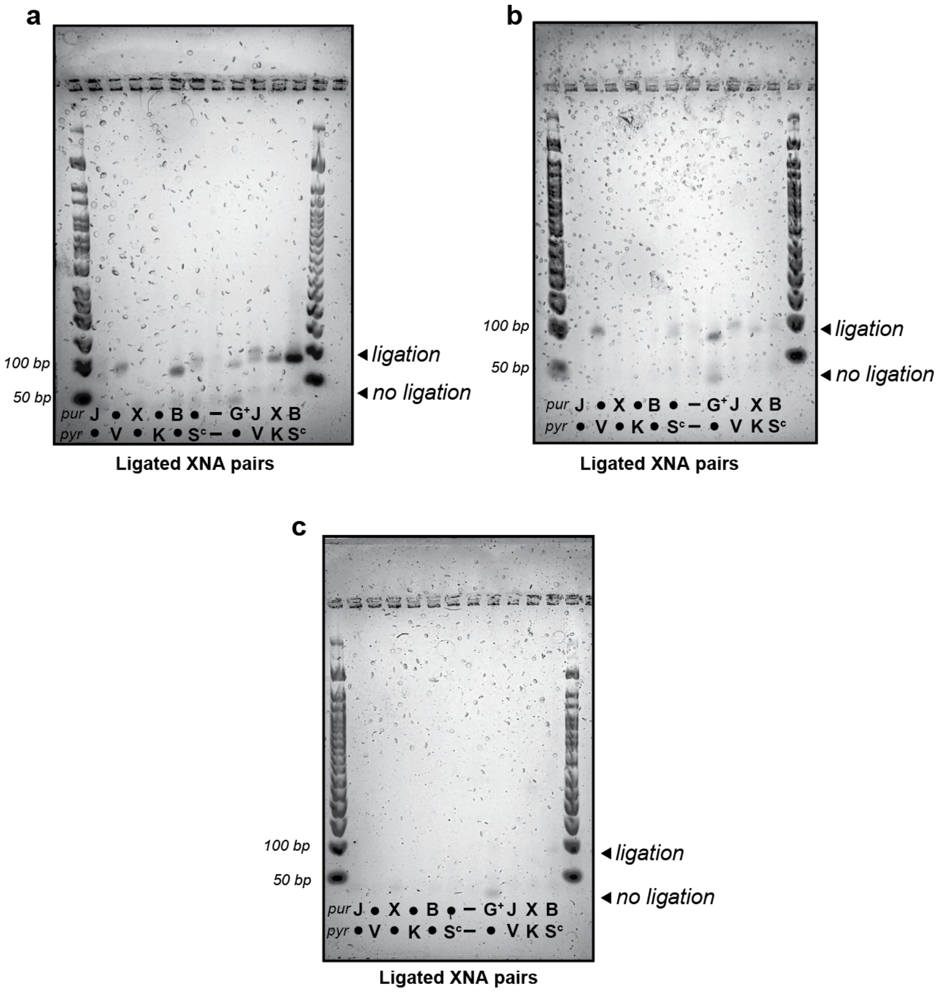

Supplementary Figure 16. Screening T3 ligase, T4 ligase, T7 ligase for JV, X^tK^n, and BS^c XNA ligation. Two blunt end hairpins that create a restriction enzyme site upon blunt ligation were purchased from IDT (5′Phos-NdeI-HP-1 and 5′Phos-NdeI-HP-2; **Supplementary Table 2**). Blunt-end ligated hairpins create an NdeI restriction site, while successfully tailed and ligated hairpins do not. This ensures that after XNA tailing, XNA ligation and NdeI/exonuclease treatment, the only products left are ligation products from properly tailed material, which prohibits the formation of the NdeI restriction site. All tailing reactions used conditions listed in **Supplementary Table 8** except S^c which was tailed for 8 h. Subsequent ligation reactions were performed using 4.7 µM of a single oligo or 2.4 µM of two oligos) with complementary tailed bases. Ligation reactions were incubated for 16 h at 16 °C using the specified DNA ligases and carried out in 1X of NEB StickTogether™ buffer which contains 7.5% (w/v) PEG 6000. Unligated materials, as well as blunt end ligated materials, were digested using a combination of Exo I (1.4 U/µL), Exo III (7.1 U/µL), Exo VIII (truncated, 0.71 U/µL), and NdeI (1.4 U/µL) for 1 h at 37°C. Enzymes were heat inactivated by incubation at 80 °C for 20 min. (**a**) T3 ligase assay (272 U/µL); (**b**) T4 ligase assay (36 U/µL); (**c**) T7 ligase assay (272 U/µL) for reactions containing single hairpins or mixture of two hairpins (as indicated). Lanes labeled with single letter abbreviation of nucleotide tailed onto 3′-end of hairpin. In

all gels, ● indicates absence of the hairpin tailed with the complementary base. Pre-tailed negative control (G^{+}/●) shows either mismatch ligation or lack of ligation and subsequent digestion for a hairpin with a 3′ single nucleotide overhang (5′Phos-HP-3′G). Blunt end negative control of a reaction ligation, (–/–) condition, containing 5′Phos-NdeI-HP-1 and 5′Phos-NdeI-HP-2 shows digestion by NdeI, and subsequent digestion by exonucleases. Gel representative of a single experimental replicate.

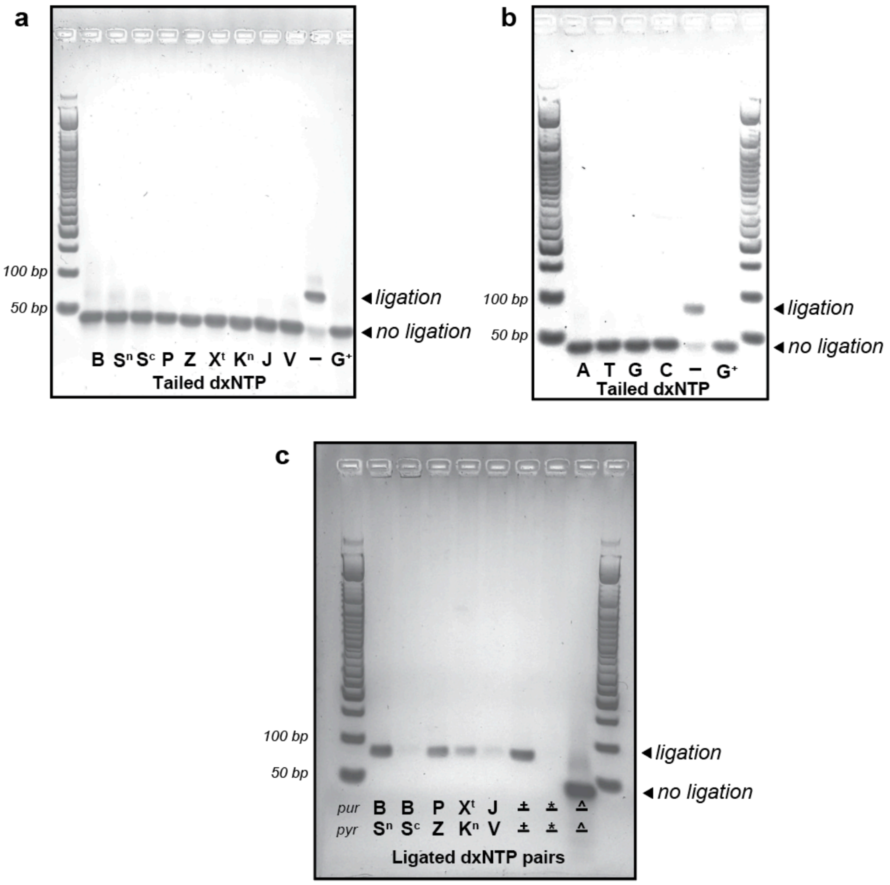

Supplementary Figure 17. Full gels of XNA tailing and XNA ligation using optimized conditions. All assays were done with a 5′-phosphorylated hairpin oligo with a 3′-blunt end, purchased from IDT (5′-Phos-11HP; **Supplementary Table 2).** Each DNA/XNA base was tailed using conditions from **Supplementary Table 8**. (**a**) Full gel for optimized XNA tailing conditions from **Fig. 2e**. Tailing completeness was measured via T4 ligation. Positive control (G^+) shows no ligation for a hairpin with a 3′ single nucleotide overhang (5′Phos-HP-3′G, lower band) while negative control (–) shows ligation of blunt-end hairpin (upper band). Samples were diluted 4-fold before loading. (**b**) Full gel for optimized DNA tailing conditions. Tailing completeness was measured by T4 ligation. Samples were diluted 8-fold before loading. (**c**) Full gel for optimized XNA ligation conditions from **Fig. 2g**. Following tailing, 2.4 µM of each oligo with a complementary tailed base (except for the B:S^c base pair which was 1.3 µM of each) was ligated using conditions from **Supplementary Table 10.** Unreacted hairpins or incomplete ligation products were removed by exonuclease treatment. Positive control (+) shows full ligation of blunt-end hairpin, while negative control (*) shows starting material with no polymerase and no ligase added, leading to full subsequent digestion by exonucleases. Starting material with no polymerase, ligase, or exonuclease added shown as a reference (^). Gels are representative of three experimental replicates.

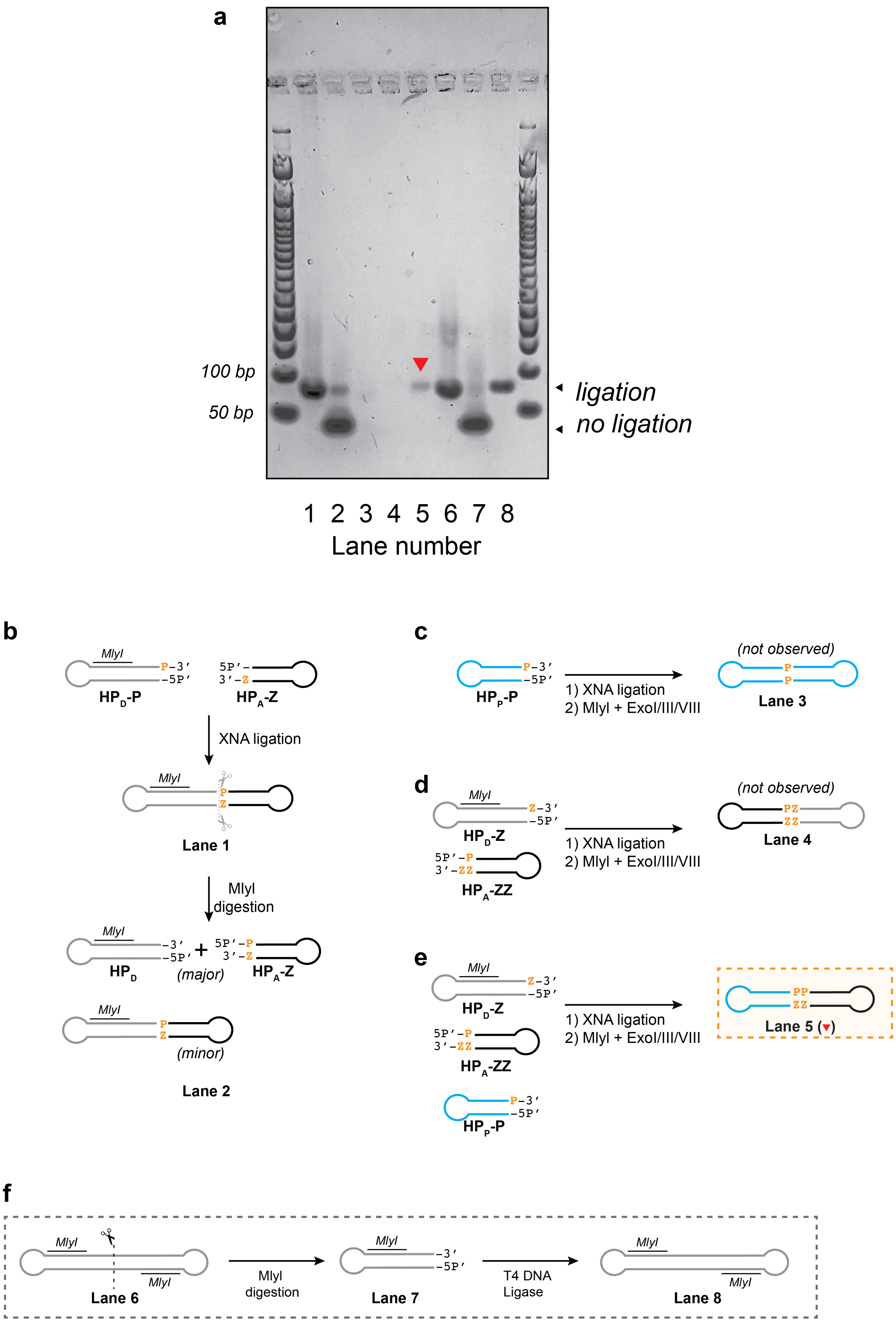
a
100 bp
50 bp
ligation
no ligation
1 2 3 4 5 6 7 8
Lane number
b
MlyI
HP_D-P
HP_A-Z
XNA ligation
Lane 1
MlyI digestion
HP_D
(major)
HP_A-Z
(minor)
Lane 2
c
HP_P-P
1) XNA ligation
2) MlyI + ExoI/III/VIII
(not observed)
Lane 3
d
HP_D-Z
HP_A-ZZ
(not observed)
Lane 4
e
Lane 5
f
Lane 6
T4 DNA Ligase
Lane 7
Lane 8

Supplementary Figure 18. Proof of concept for XNA tailing and XNA ligation cycling to insert two consecutive P≡Z base pairs. (**a**) Agarose gel showing key steps in consecutive XNA insertion. Each lane is described in the schematics that follow. (**b**) A hairpin containing an MlyI restriction site adjacent to the site of XNA ligation is used (donor hairpin, HP_D). MlyI is a type IIS restriction enzyme (5′-**GAGTC**NNNNN↓-3′) that leaves a blunt end after cutting. A donor hairpin with an MlyI site and an acceptor hairpin were tailed with P and Z respectively (generating HP_D-P, HP_A-Z), ligated and treated with exonucleases following the optimized conditions described in this work, and then purified (**lane 1**). The purified construct contains a single P≡Z base pair insertion. Product from lane 1 was digested using MlyI, resulting in products observed in **lane 2**: (major product) blunt end hairpin products HP_D (regenerated donor hairpin) and HP_A-Z (acceptor hairpin with a 3′- P≡Z base pair); (minor product) undigested product from lane 1. (**c**) Separately, a donor hairpin without an MlyI site was prepared by XNA tailing (HP_P-P). XNA ligation followed by MlyI and exonuclease treatment does not result in formation of a ligation product (**lane 3**). (**d**) In a second round, reaction product mixture from lane 2 was tailed with Z to produce Z-tailed donor hairpin (HP_D-Z) and Z-tailed PZ-acceptor hairpin (HP_A-ZZ). XNA ligation followed by MlyI and exonuclease treatment does not result in formation of a ligation product (**lane 4**). Minor ligation product previously observed in lane 2 is also no longer observed, suggesting this additional MlyI + exonuclease digestion round effectively removes MlyI-containing products. (**e**) Tailed donor and acceptor hairpins (HP_D-Z, HP_A-ZZ, HP_P-P) were ligated. XNA ligation followed by MlyI and exonuclease treatment results in formation of a product with two consecutive P≡Z base pair insertions (**lane 5, ▼**). Incorrect ligation product from two donor hairpins (HP_D-Z + HP_P-P) would not be present since HP_D-Z contains an MlyI site. (**f**) MlyI cycling control reactions were carried out to assay how yield is generally affected by multiple ligation cycles. Blunt-end HP_D was ligated using T4 DNA ligase (blunt-end ligation), treated with exonuclease, and purified (**lane 6**). Reaction product was then digested with MlyI (**lane 7**), then subjected to an additional round of blunt-end ligation using T4 DNA ligase (**lane 8**). Consecutive rounds of MlyI digestion and ligation result in a visible decrease in blunt end ligation product yield. For additional details regarding hairpin sequences used, please see methods section "Consecutive insertion of XNA base pairs using MlyI Type IIS restriction enzyme". Nucleic acid end abbreviation: 3′ indicates 3′-OH, 5P′- indicates 5′-PO_4. Gel representative of a single experimental replicate.

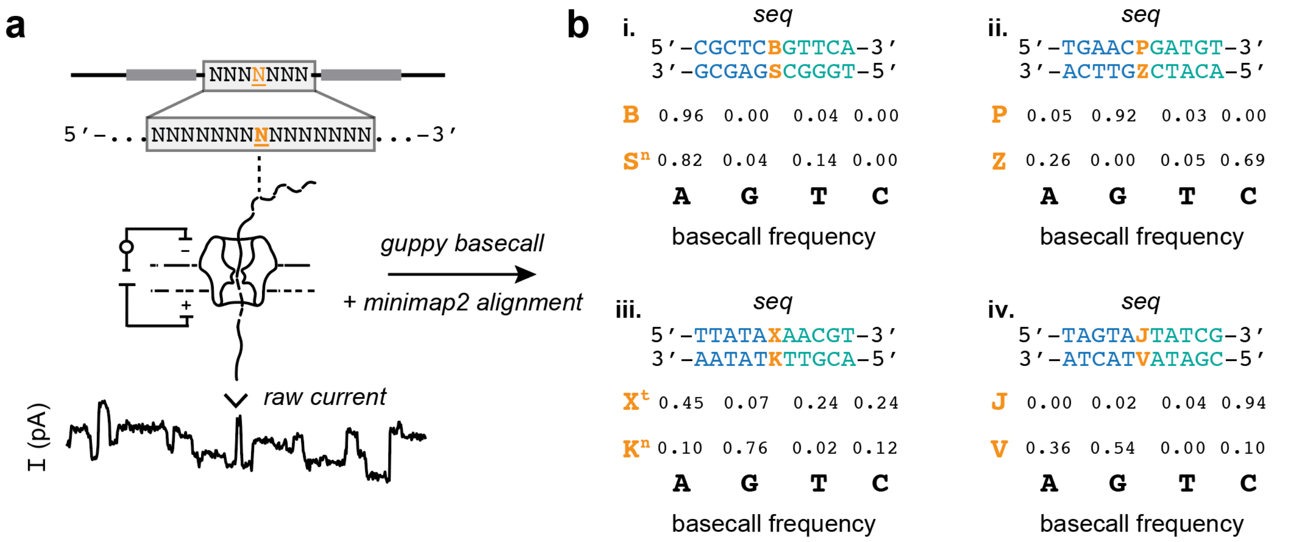

Supplementary Figure 19. Examples of basecalling XNA sequences with guppy. (**a**) ONT guppy was trained to basecall sequences composed only of standard nucleic acids (A, T, G, or C). We enzymatically synthesized sequences containing various XNA base pairs (B≡S^n, P≡Z, X^t≡K^n, J≡V) to determine what canonical base is assigned to each of these XNAs. Guppy with high accuracy configuration (dna_r9.4.1_450bps_hac.cfg) was used for basecalling, and minimap2 was used to align sequences. (**b**) Example sequence context surrounding an XNA base with the frequency of standard basecalls assigned to each XNA: i) B≡S^n base pair most frequently assigned to A:A; ii) P≡Z base pair most frequently assigned to G:C; iii) X^t≡K^n base pair most frequently assigned to A:G; iv) J≡V base pair most frequently assigned to C:G. Results suggest pyrimidine:purine guppy basecalling trends do not persist between standard and non-standard nucleic acids.

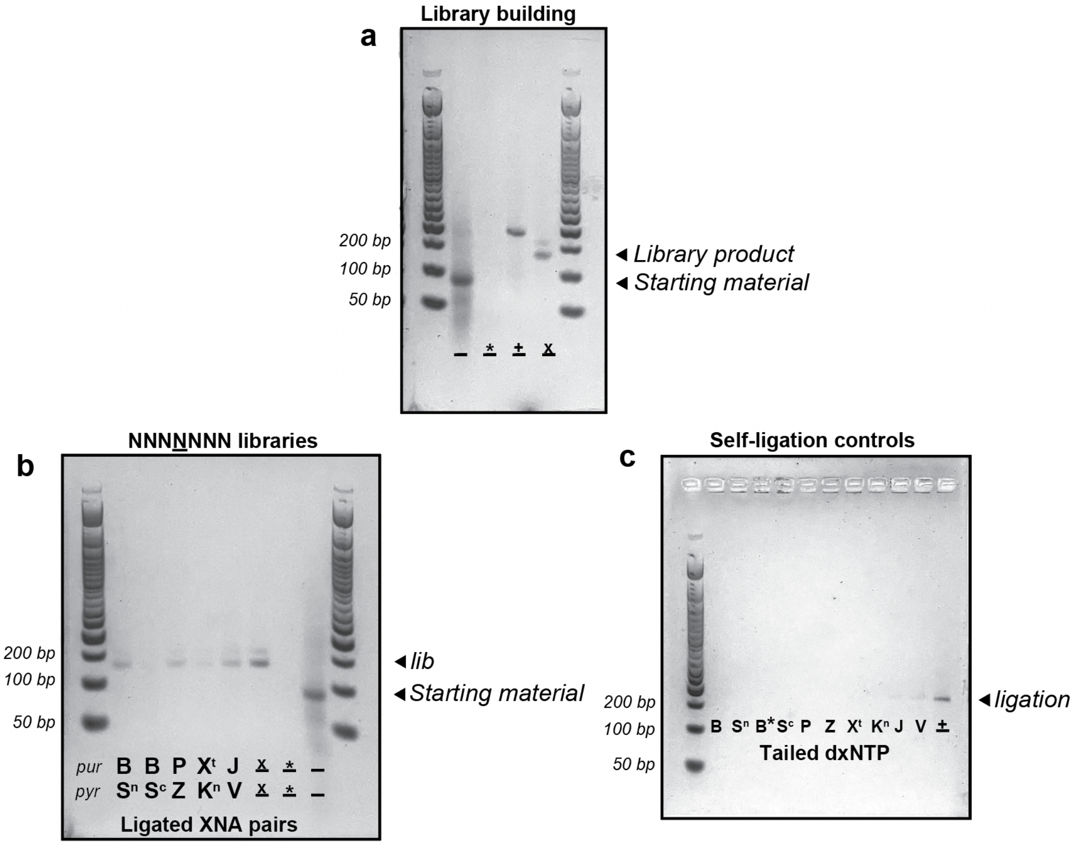

Supplementary Figure 20. Full gels of NNNNNNN library construction for nanopore sequencing. All assays were performed using NNN-pool oligos as starting material, listed in **Supplementary Table 3-5.** (**a**) Steps involved in library building process exemplified using blunt-end hairpins. 8-fold diluted starting material (–) for library building shown as reference. Starting material without ligase shows subsequent full digestion via exonuclease digestion (*). If ligase is added and ligation is successful, a subsequent exonuclease digest leaves only the ligated product (±) which does not have free 3′-ends. After removing one or both of the hairpin ends via restriction enzyme digestion, the final library product remains as the major product, with incomplete removal of the hairpin ends as the minor product (X). Ligation and subsequent processing were done using the methods outlined previously. (**b**) Complete NNNNNNN library products for all XNA base pairs and blunt end ligation library sequenced in this work. (**c**) Self-ligation for library hairpins to check for incomplete tailing and pyrophosphorolysis products. Library hairpins were tailed with the listed XNA using conditions listed in **Supplementary Table 8,** and 4.7 µM of each hairpin (except B* and Sc at 2.6 µM) was ligated to itself using the conditions listed in **Supplementary Table 10.** Blunt end ligation (±) was included as a negative control. Unreacted hairpins or incomplete ligation products were removed via exonuclease treatment. Minimal self-ligation is observed for XNA-tailed NNN-pools, with J and V-tailed NNN-pools showing the most self-ligation compared to all the other XNA-tailed NNN-pools. These data suggest that the major product of XNA ligation with complementary sets is desired heteroligation products. After sequencing, self-ligation products are identified by their pool barcodes and are removed prior to model building. Gels representative of a single experimental replicate.

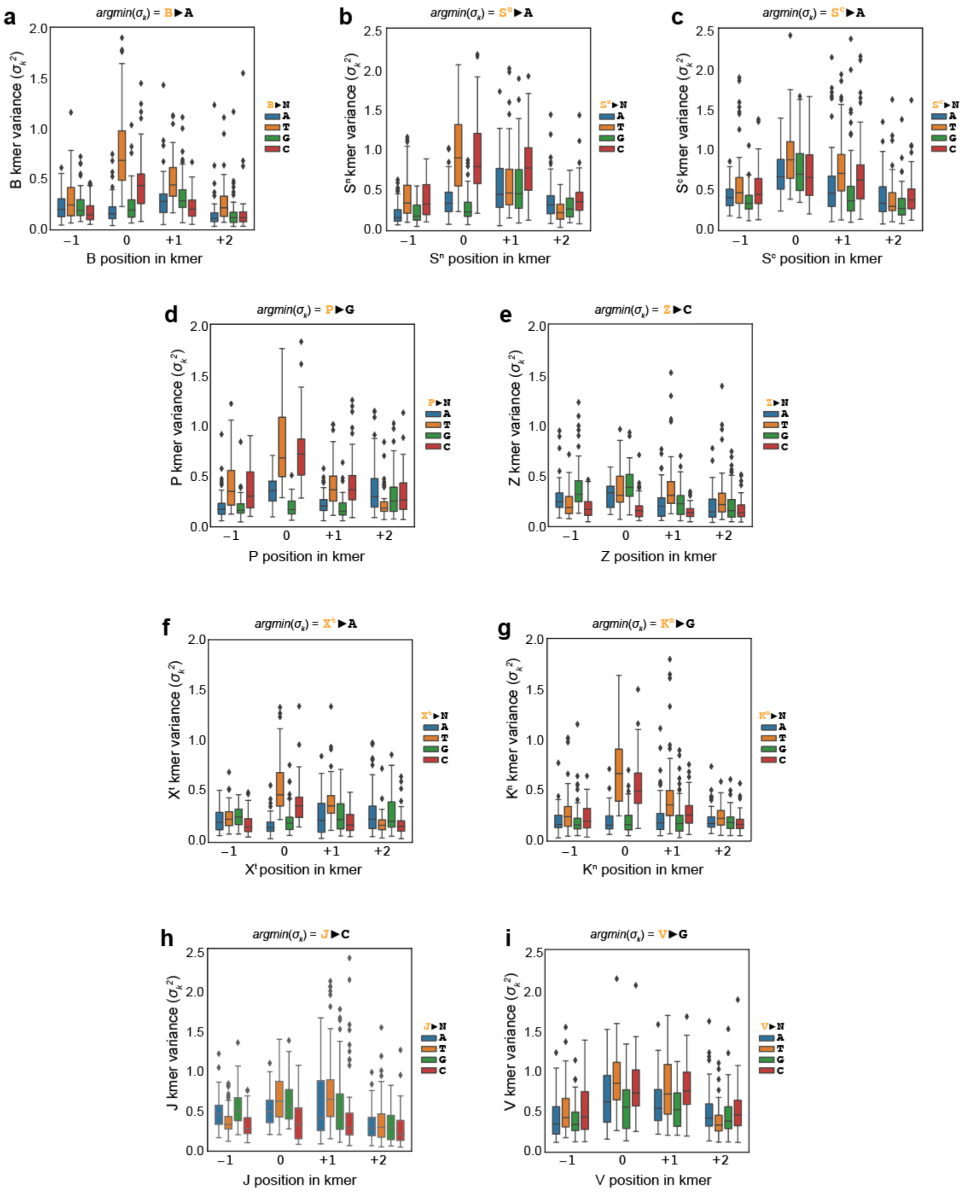

Supplementary Figure 21. Variance minimization for segmentation steps of signal-to-sequence mapping. Signal-to-sequence mapping was performed using Tombo. Tombo uses an informed kmer model to improve the accuracy of signal-to-sequence mapping. Without a prior model, segmentation requires assigning each XNA to a standard base. Improper segmentation leads to inaccurate model parameter estimates. To minimize bias in segmentation, we assigned each XNA to the standard base that minimized the total variance in observed kmer signal levels. (**a**-**i**) Boxplots of observed variance in kmer signal levels for each XNA base (B, S^n, S^c, P, Z, X^t, K^n, J, V respectively) at each position within a 4-nt kmer (-1: NNNN; 0: NNNN; +1: NNNN; +2: NNNN). Segmentation assignments used are indicated above each panel. Results are generally in agreement with guppy's basecalling observations and indicate that standard pyrimidine:purine pairing assignments fail to properly describe signal-level observations. With optimum choice of standard base assignment.

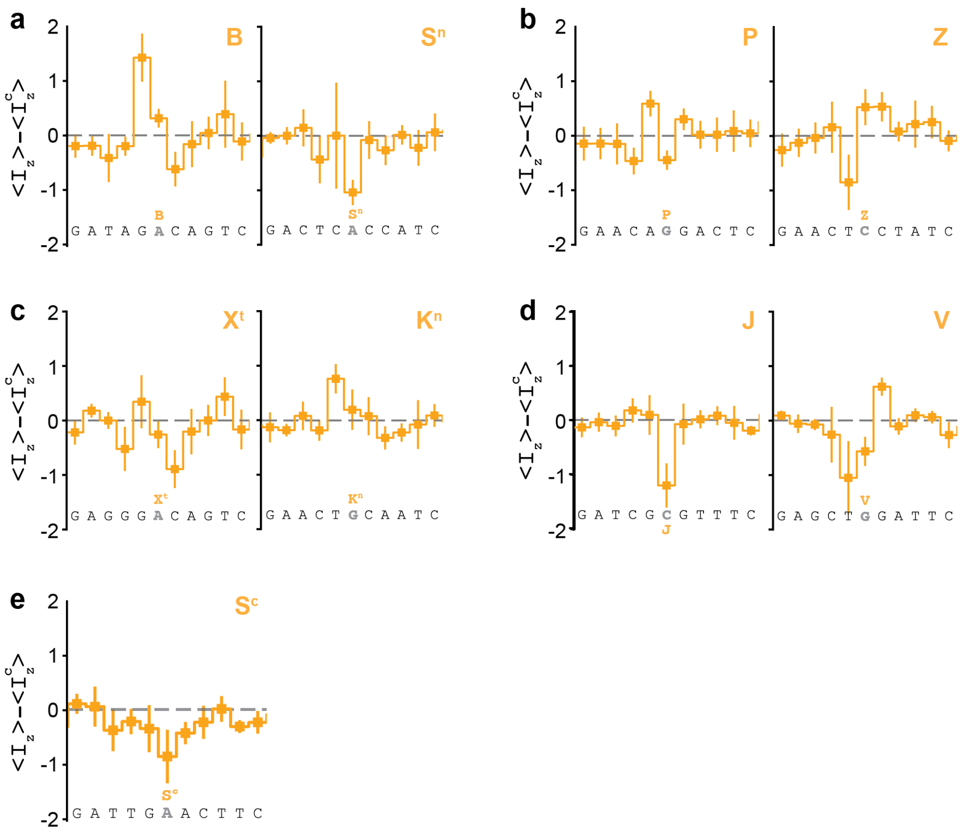

Supplementary Figure 22. Example trace of signal deviation from the standard model. (a-e) Example traces showing how observed normalized signal for sequences that contain a xenonucleotide ($\langle I_z \rangle$) deviates from the expected standard DNA model signal ($\langle I_z^c \rangle$) of the most similar standard base for B (*n* = 70), S^n (*n* = 22), P (*n* = 45), Z (*n* = 44), X^t (*n* = 75), K^n (*n* = 12), J (*n* = 10), V (*n* = 10), and S^c (*n* = 11). Most similar standard base shown below xenonucleotide. Error bars indicate standard deviation.

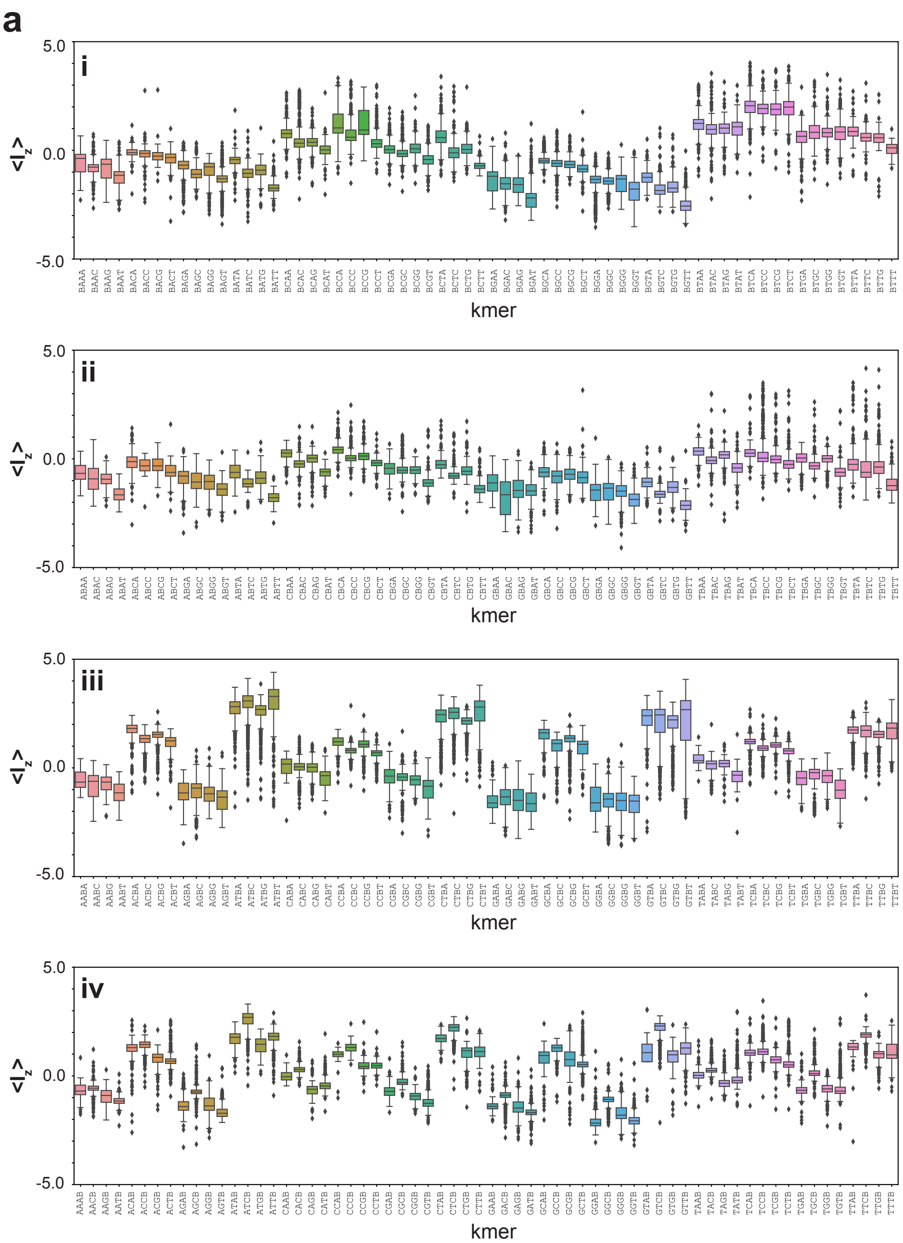
a
i
ii
iii
iv
5.0
0.0
-5.0
<I>z
kmer

b

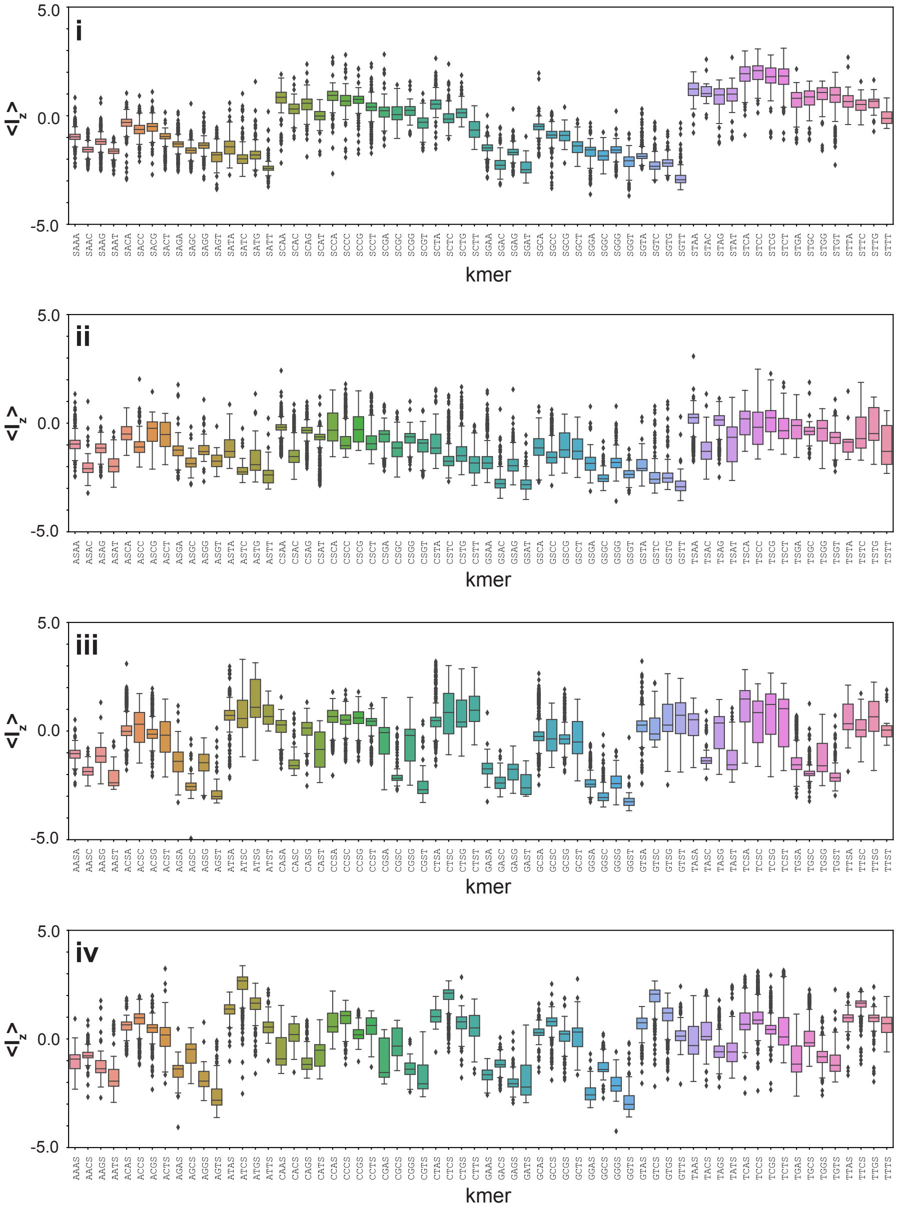

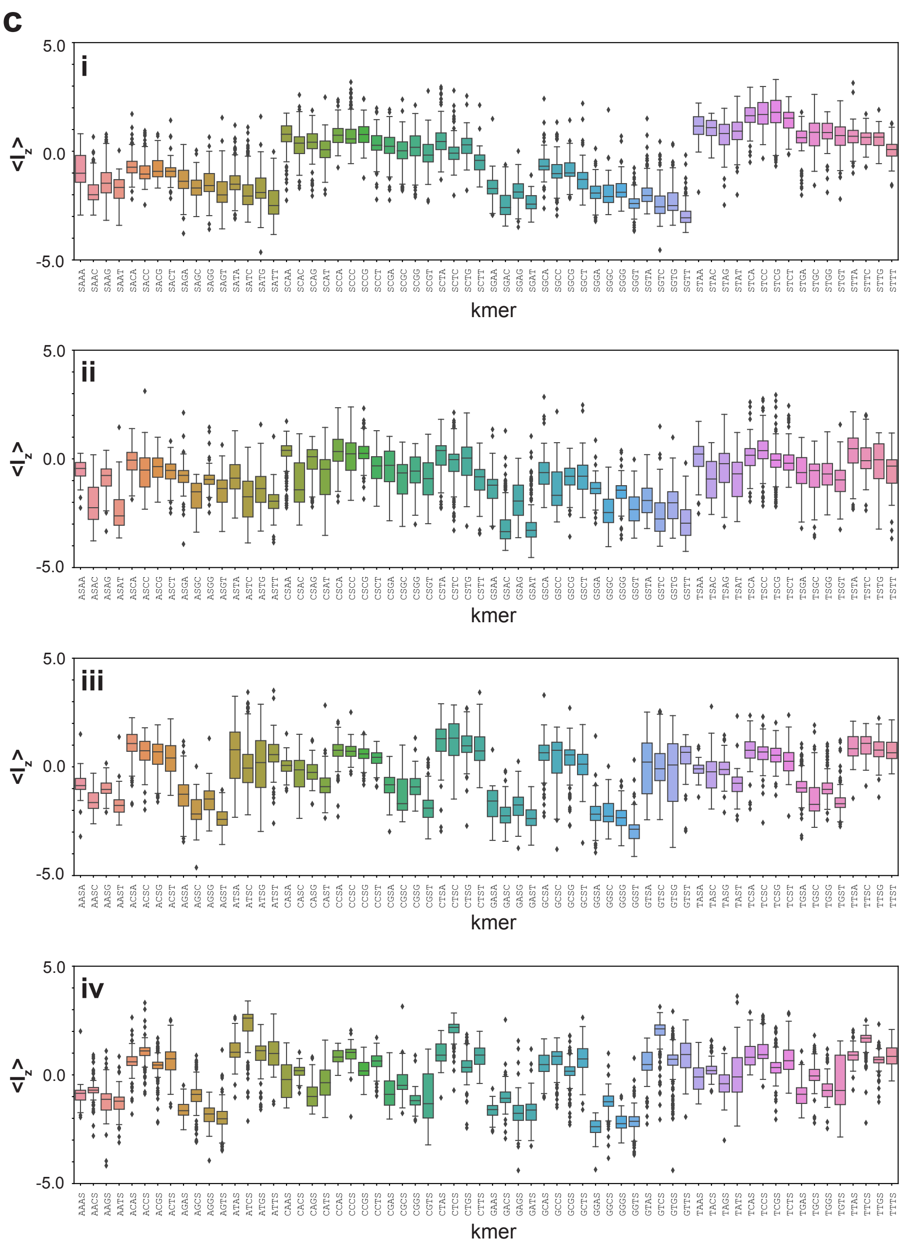
c
i
ii
iii
iv
5.0
0.0
-5.0
kmer

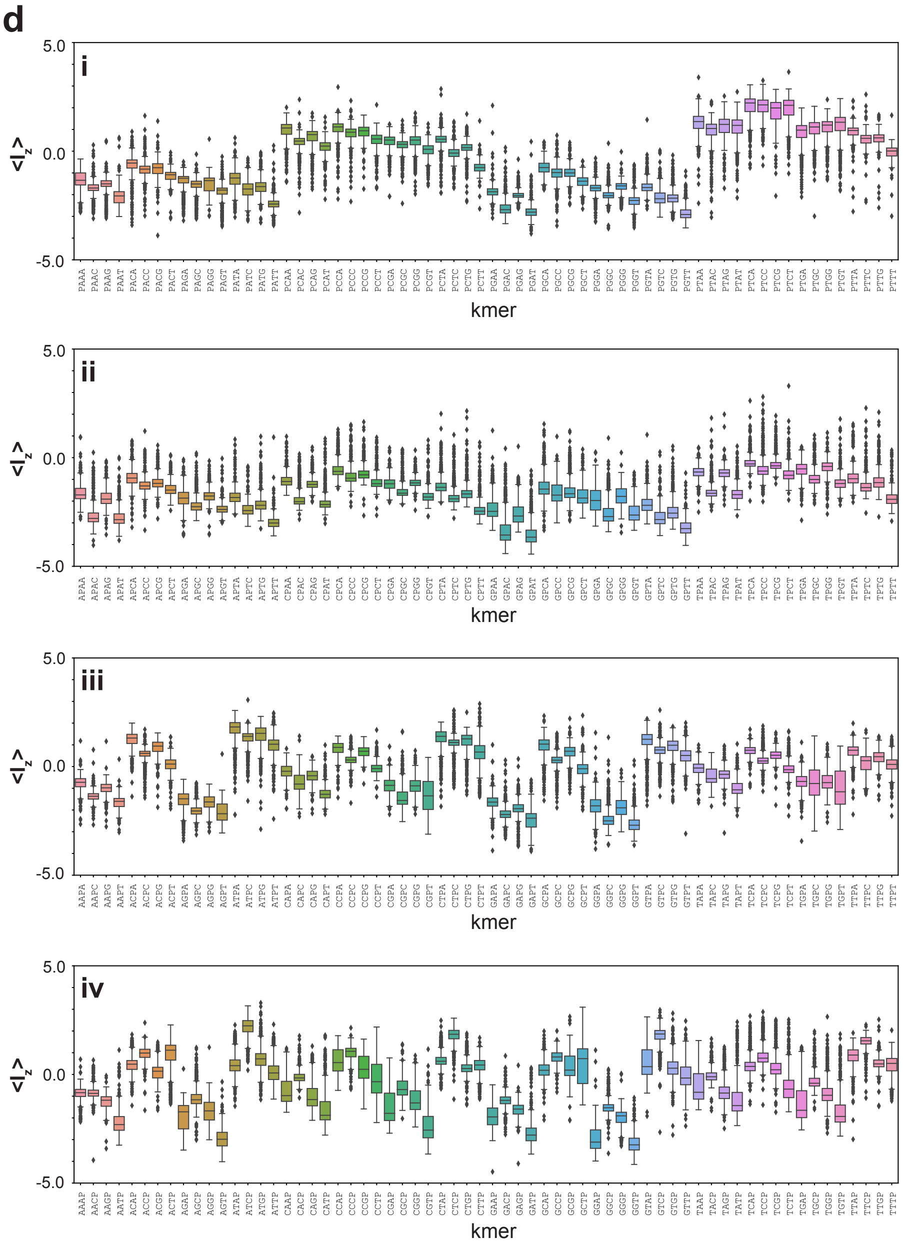

d
i
ii
iii
iv
<I_z>
kmer
5.0
0.0
-5.0

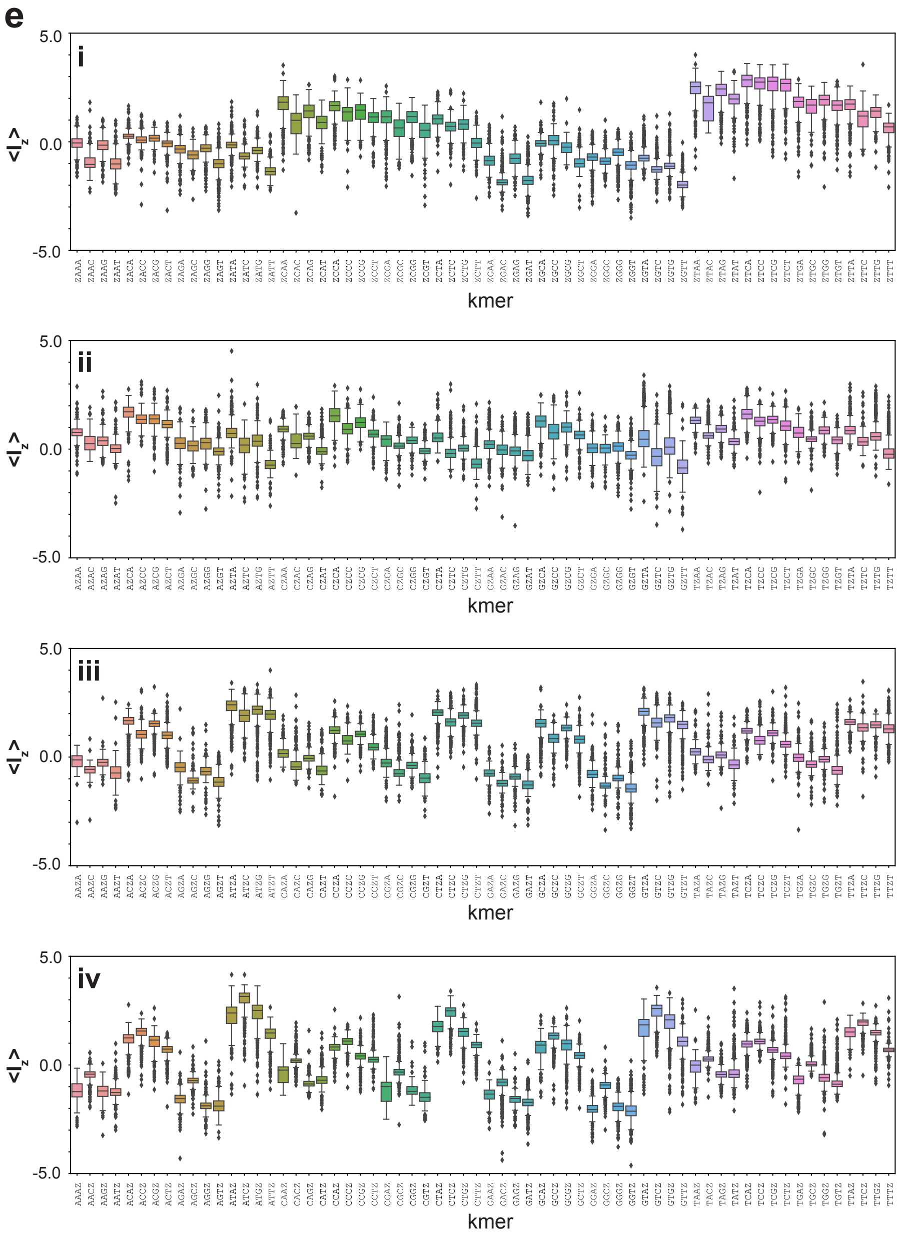
e
i
ii
iii
iv
5.0
0.0
-5.0
<I>z
kmer

f

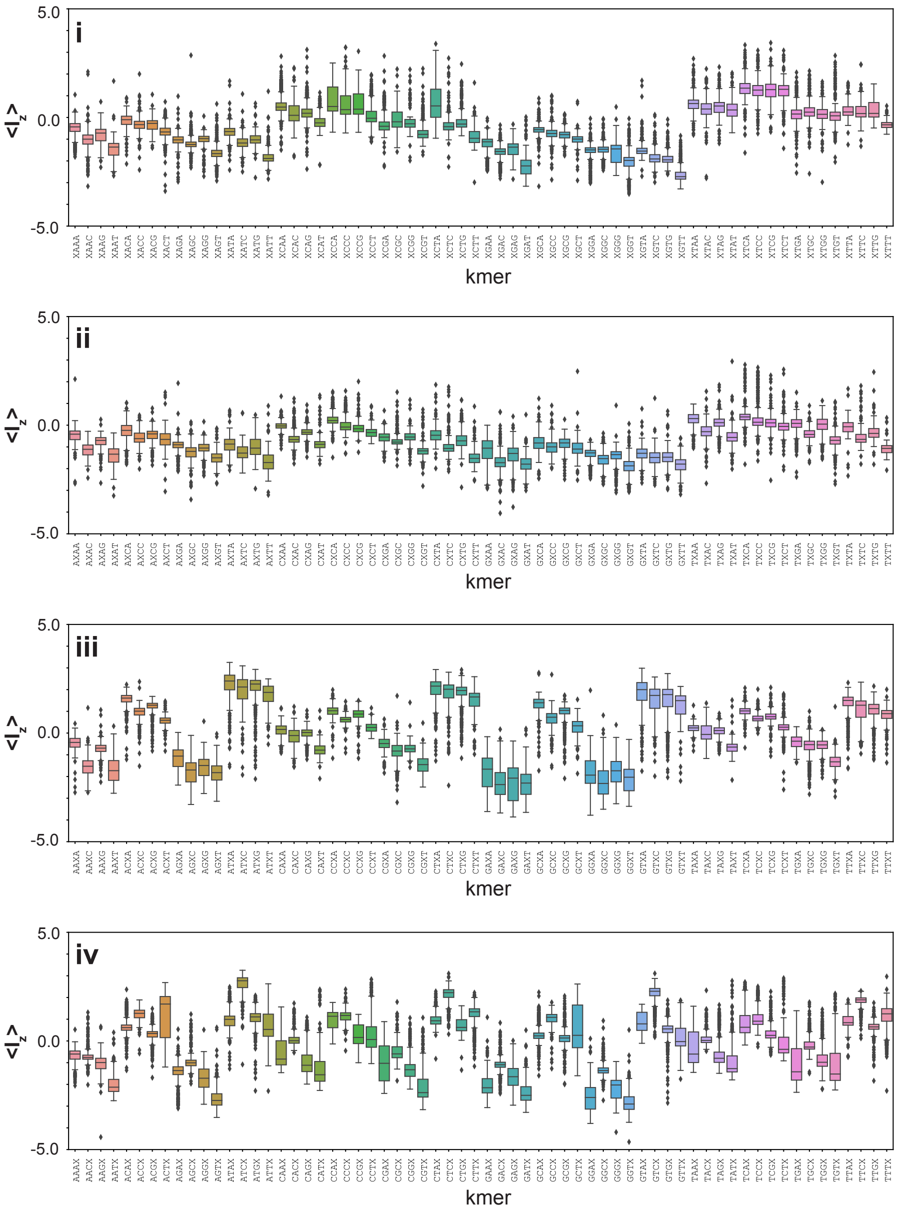

g

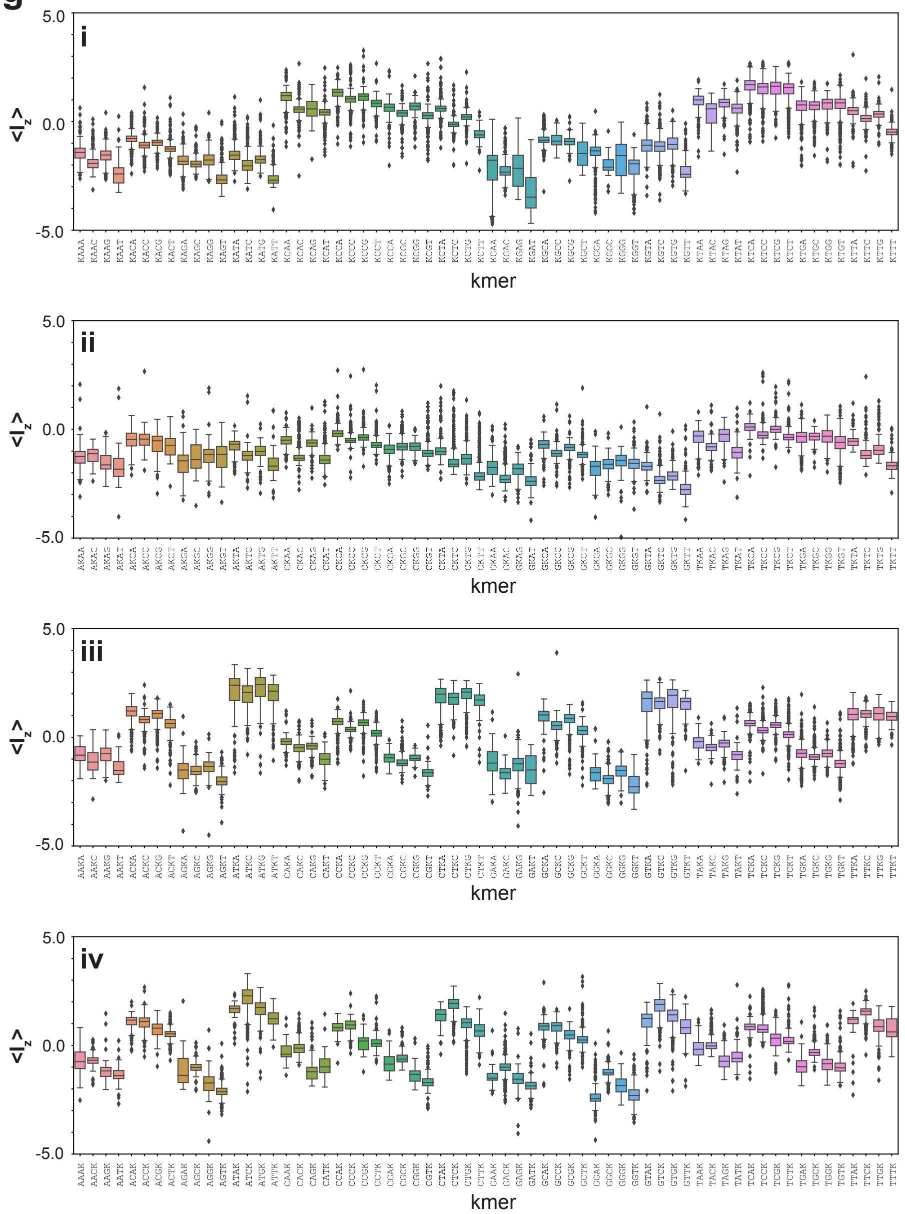

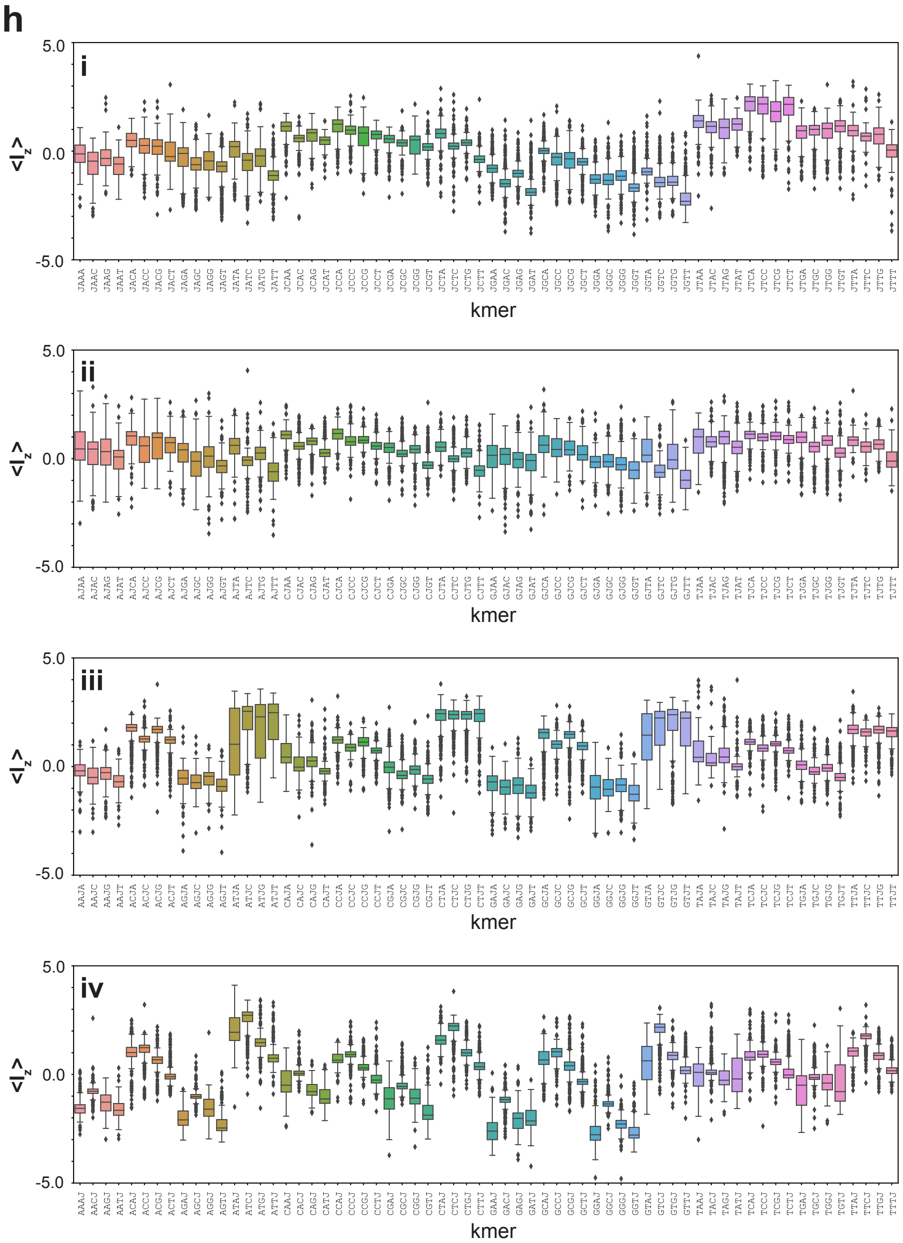
h
i
ii
iii
iv
5.0
0.0
-5.0
<I_z>
kmer

i

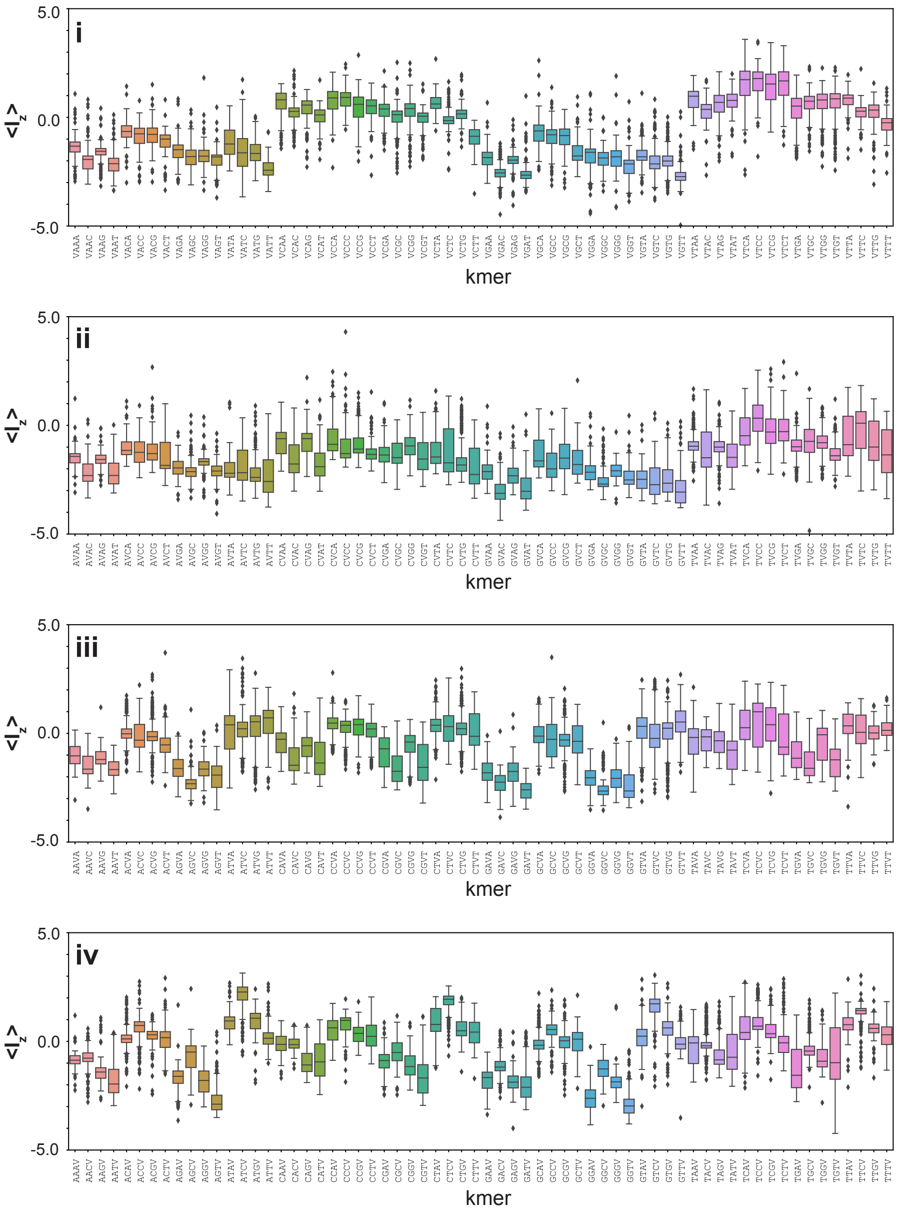

Supplementary Figure 23. Distribution of observed 4-nt kmers signal levels for all XNA bases. To build kmer models, NNNNNNN libraries were sequenced and then mapped to library references. Heptamer sequences that contained XNAs were decomposed to 4-nt kmers (NNNN, NNNN, NNNN, NNNN) and assigned the observed signal levels $\langle I_z \rangle$. Kmer models were built from all observations of each kmer among all sequences observed in the NNNNNNN library sets. (**a**-**i**) Panels are shown for kmers containing each of the XNA bases (B, S^n, S^c, P, Z, X^t, K^n, J, V respectively). Boxplot showing distribution of observed signal levels for each kmer where the XNA is in the: i) -1 position (NNNN); ii) 0 position (NNNN); iii) +1 position (NNNN); or iv) +2 position (NNNN). Boxplots show 25th and 75th percentiles (box), median (line), 10th and 90th percentile (whiskers), and outliers (points). Boxes colored only for visual effect. Each boxplot was generated from a maximum of 1000 observations for each kmer (n_{max} = 1000). Full statistics available in provided model files (**Supplementary Data 1**).

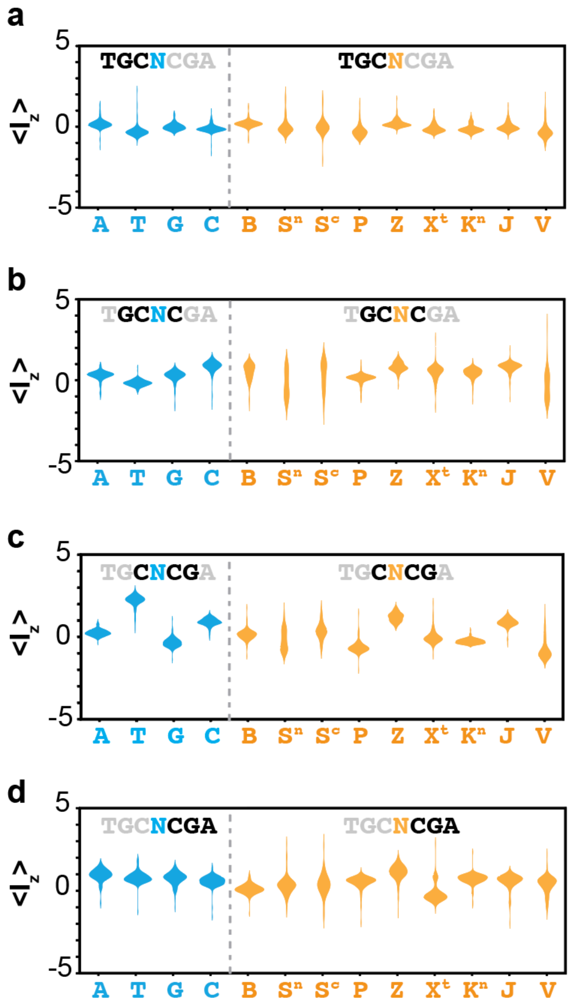

Supplementary Figure 24. Example comparison of observed 4-nt kmer signal levels across a full heptamer sequence. For a 4-nt kmer (NNNN), the central position is denoted as the 0th position. 4-nt kmer model used in this work sets the second base as the 0th position (-1, 0, +1, +2). Any heptamer sequence can therefore be described as a sequence of kmers. Violin plots show normalized signal distributions for observed kmers that constitute an example heptamer sequence (TGC**N**CGA, where **N** = A, T, G, C, B, S^n, S^c, P, Z, X^t, K^n, J, V). Each subplot shows a sliding window of kmers across the heptamer sequence. (**a**) First kmer in the heptamer sequence, containing variable base in the +2 kmer position. (**b**) Second kmer in the heptamer sequence, containing variable base in the +1 kmer position. (**c**) Third kmer in the heptamer sequence, containing variable base in the +0 kmer position. (**d**) Fourth kmer in the heptamer sequence, containing variable base in the -1 kmer position. Additional information explaining kmer model and heptamer sequences can be found with **Supplementary Table 16**.

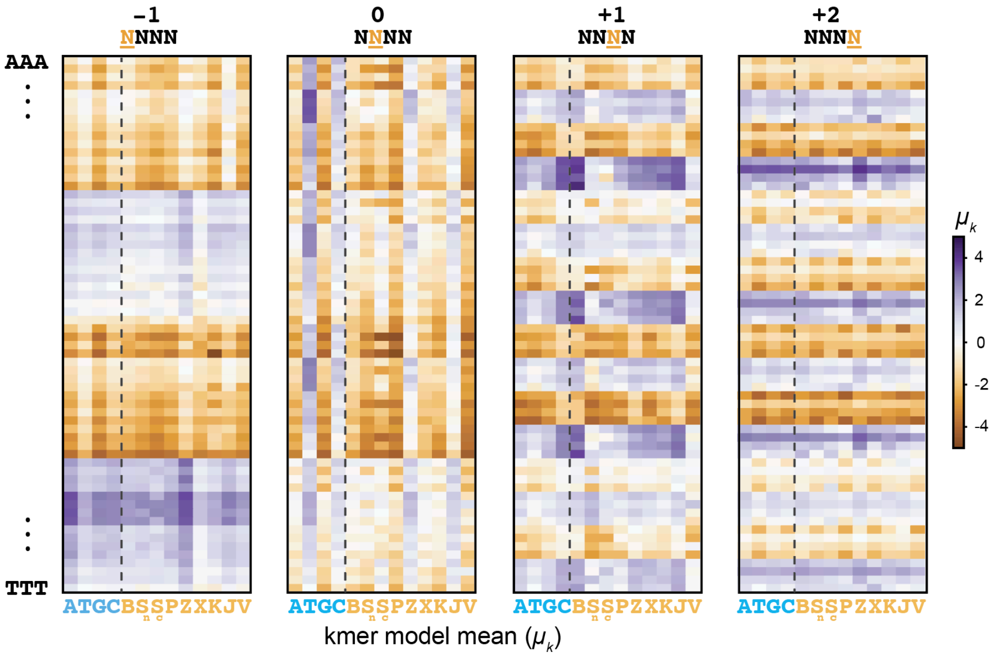

Supplementary Figure 25. Full heatmap showing measured 4-nt kmer model KDE means including standard bases. Heatmap shows μ_k estimates for all standard bases (A, T, G, C) alongside non-standard bases (B, S^n, S^c, P, Z, X^t, K^n, J, V) measured in this work. Subpanels are sorted by the position of the substituted base in the kmer (-1, 0, +1, and +2). For each kmer, N is denoted in the x-axis and the remaining NNN is denoted by the row (sorted alphabetically, i.e. A, C, G, T). Rows are sorted in alphabetical order based on the non-substituted base (A, C, G, T) from NNN = AAA to NNN = TTT. Positive μ_k values are shown in deeper shades of purple while negative values are shown in deeper shades of orange.

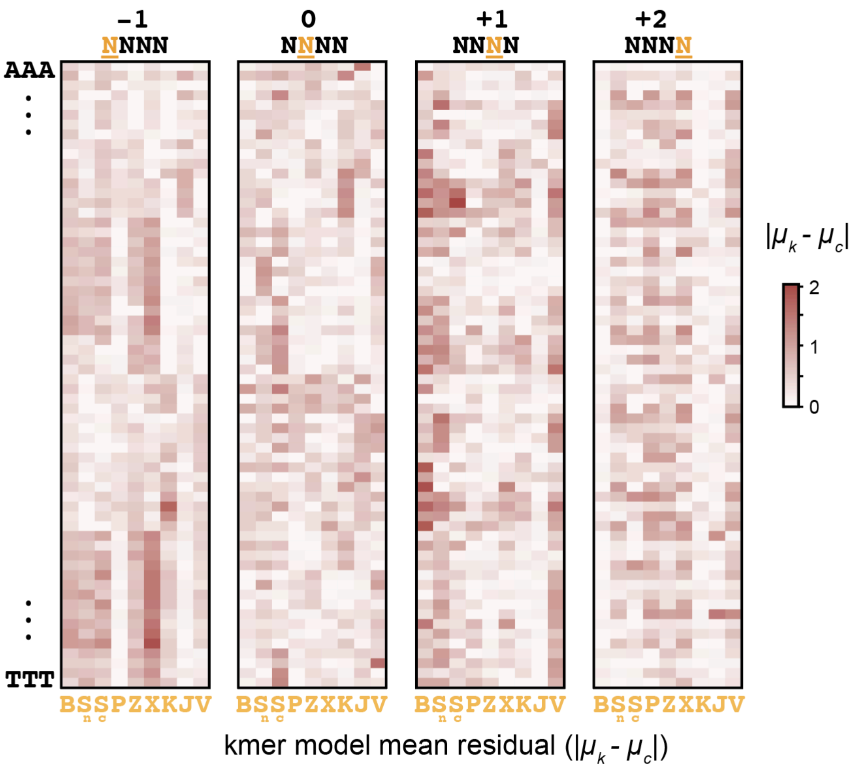

Supplementary Figure 26. Full heatmap showing measured 4-nt XNA kmer model deviation from standard model. Heat maps showing absolute value of kmer model mean residuals ($|\mu_k - \mu_c|$), where μ_k is the mean level of kmer *k*, and μ_c is the mean level of kmer *k* where the XNA is substituted for the most similar standard base. Deeper shades of red indicate XNA-containing kmer emits signal with larger deviations from the standard kmer. Heatmap is binned by position within the kmer that contains the XNA base (-1, 0, +1, or +2). For each kmer, N is denoted in the x-axis and the remaining NNN is denoted by the row (sorted alphabetically, i.e. A, C, G, T).

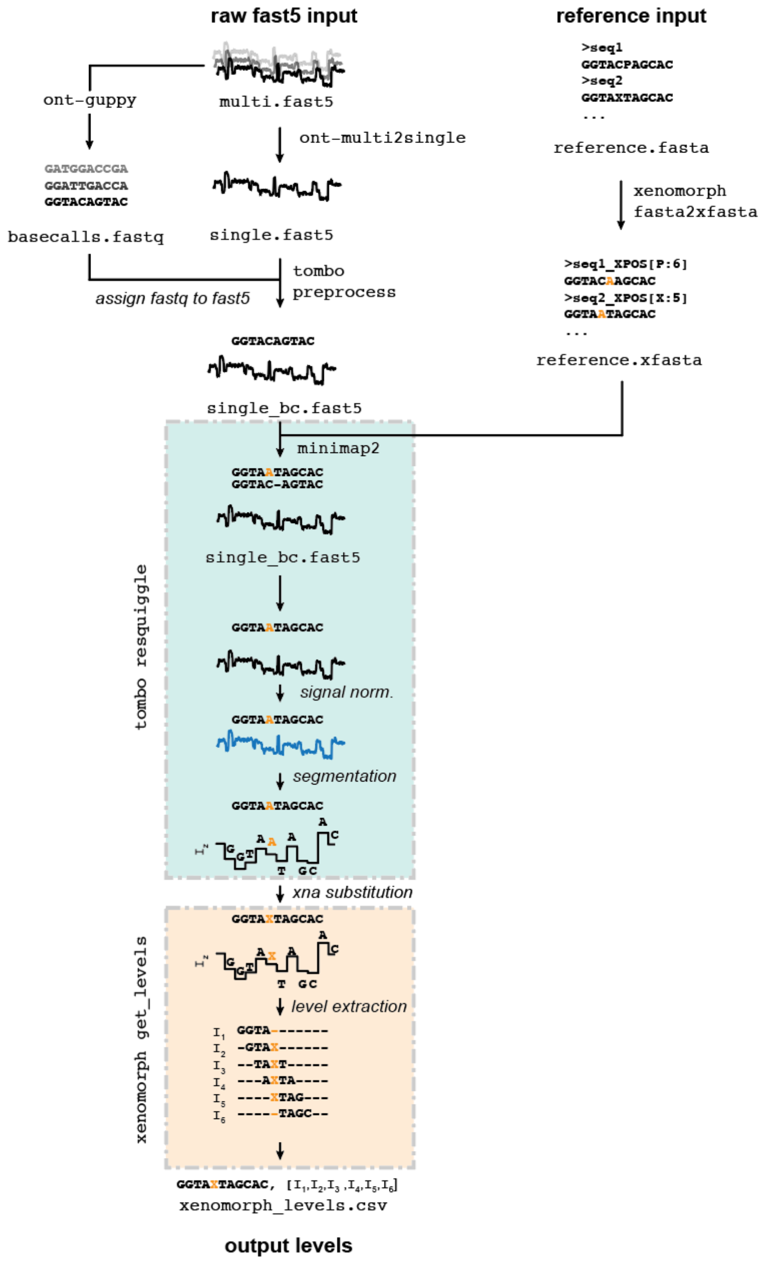

Supplementary Figure 27. The Xenomorph preprocessing pipeline. Flow diagram depicting the user input and outputs of the Xenomorph preprocessing pipeline. `Xenomorph preprocess` integrates basecalling, raw multi-to-single fast5 conversion, reference sequence fasta conversion, segmentation, and level assignment into a single command. Level extracted output files from `xenomorph preprocess` are inputs to basecalling through alternative hypothesis testing using `xenomorph morph`. Separating the preprocessing steps from alternative hypothesis testing allows users to experiment with basecalling using various model parameter settings or with alternative models without having to rerun the slower signal extraction steps. `xenomorph preprocess` uses guppy for initial basecalling, minimap2 for initial basecall-reference alignment, and ONT Tombo for signal normalization and signal-to-sequence alignment.

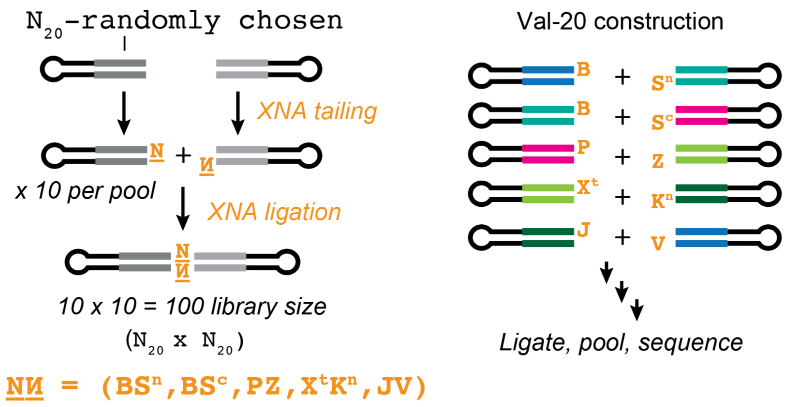

Supplementary Figure 28. Construction, pooling, and sequencing of the Val-20 6-letter DNA libraries. A validation library (Val-20 set) was constructed through XNA tailing and XNA ligation reactions (**Supplementary Table 8, 10**). In this library, XNA base pairs were embedded in a sequence context with no homology to the training dataset libraries. Sequence contexts were computationally generated by randomly choosing 20 nt from A, T, G, C with uniform probability. The 20-nt sequences generated are shown in **Supplementary Table 6**. After ligation, each pool contained 100 unique sequences of XNA base pairs embedded within this randomly generated 20mer context (i.e., 20 nucleotides randomly chosen from standard set (A, T, G, or C) with uniform probability; full sequences can be found in **Supplementary Data 2**). Validation libraries were pooled together and sequenced on an Oxford Nanopore MinION commercial nanopore device using a MinION flow cell.

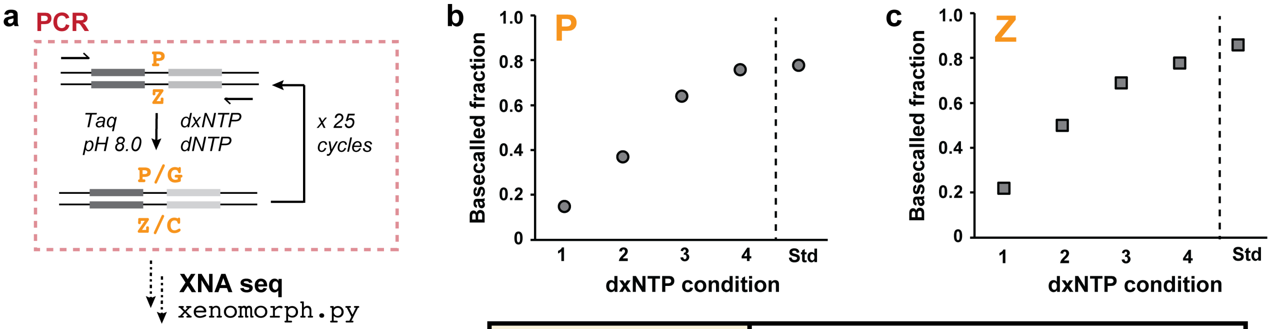

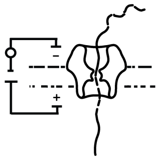

		[dxNTP] (mM)		[dNTP] (mM)			
#	Condition	dPTP	dZTP	dATP	dTTP	dGTP	dCTP
1	No dxNTP	0	0	0.2	0.2	0.2	0.2
2	Limiting	0.025	0.025	0.2	0.2	0.2	0.2
3	Equimolar	0.2	0.2	0.2	0.2	0.2	0.2
4	Optimal	0.6	0.05	0.1	0.1	0.1	0.6

Supplementary Figure 29. PCR amplification and sequencing of a DNA template with a P≡Z base pair. (**a**) Synthetic template DNA containing a P≡Z base pair was amplified with Taq polymerase in a pH 8.0 buffer with varying concentrations of dxNTP and dNTP (**Supplementary Table 22, 23**). PCR products were sequenced on a MinION nanopore flow cell then basecalled for PZ detection. Read fractions that basecalled to (**b**) P and (**c**) Z for each condition are shown. PCR conditions differ only by concentration of dxNTP and dNTPs used. The remaining fraction for each base corresponds to G and C basecalls (the most likely standard mutation for P and Z), respectively. Unamplified, synthetic P≡Z DNA was sequenced as a positive control (Std) for basecalling. Source data are provided as a Source Data file.

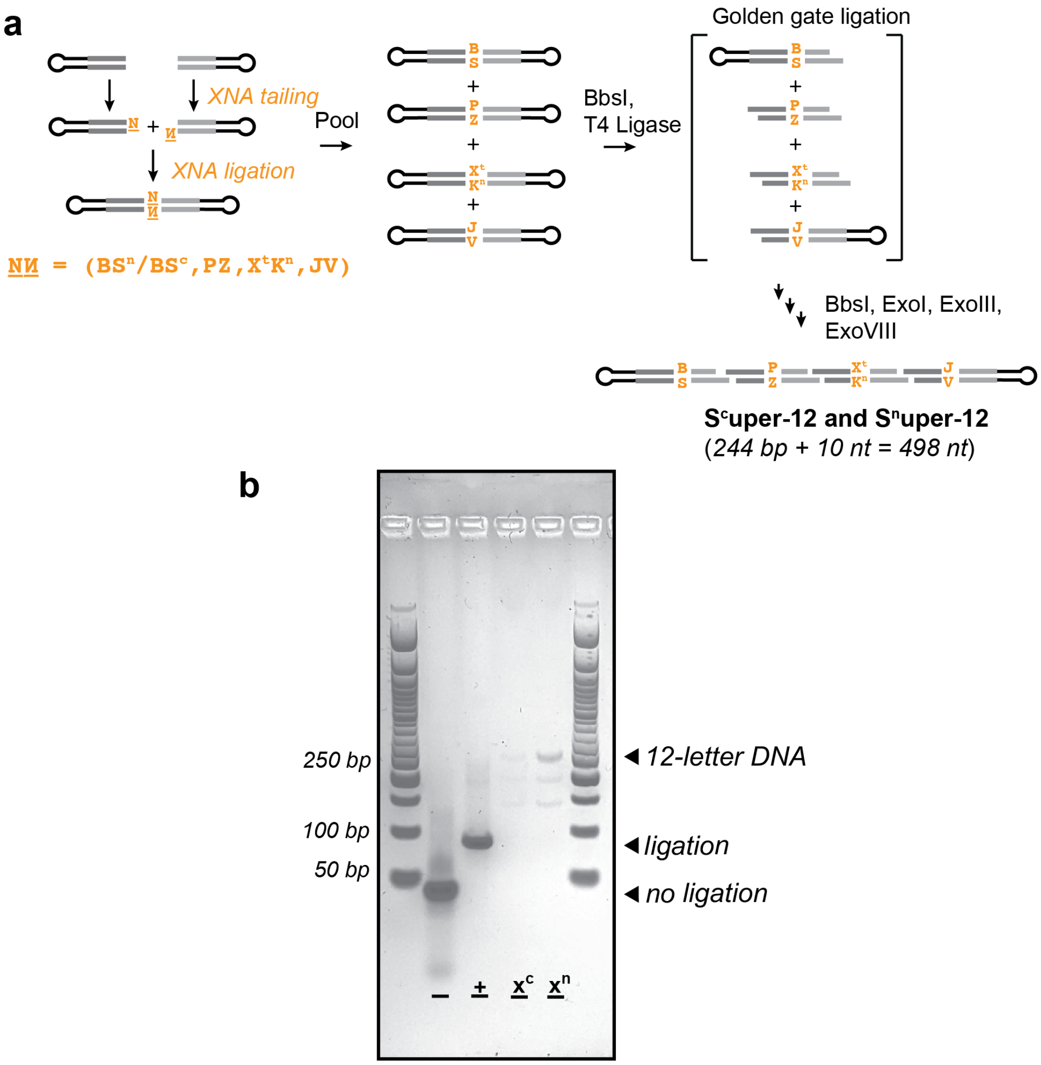

Supplementary Figure 30. Construction of 12-letter DNA for nanopore sequencing. All assays were performed using 12-letter DNA construction oligos as starting material, listed in **Supplementary Table 7.** (**a**) Oligos are tailed with a dxNTP and ligated to a complementary pair forming a sequence with a single xenonucleotide base pair insertion. These oligos contain Golden Gate sites. Four single insertion constructs undergo Golden Gate ligation to form a single dsDNA sequence containing all 12 DNA letters. To remove intermediary 6-letter, 8-letter, or 10-letter DNA products, unsuccessfully assembled hairpins are digested by restriction endo and exonucleases. (**b**) Agarose gel showing key steps in construction of 12-letter DNA. Starting material (–) shown as a reference. An example of a successful xenonucleotide tailing and ligation reaction resulting in an insertion of a single P≡Z base pair shown; subsequent exonuclease digestion leaves only the ligated product (±) which does not have free 5′- or 3′-ends. Lane (x^c) shows Golden Gate ligation product of S^{c}uper-12 and lane (x^n) shows Golden Gate ligation product of S^{n}uper-12. While smaller assembled products are visible, only those that fully align to the full span of 12-letter DNA product are considered for basecalling analysis. Gel representative of two experimental replicate.

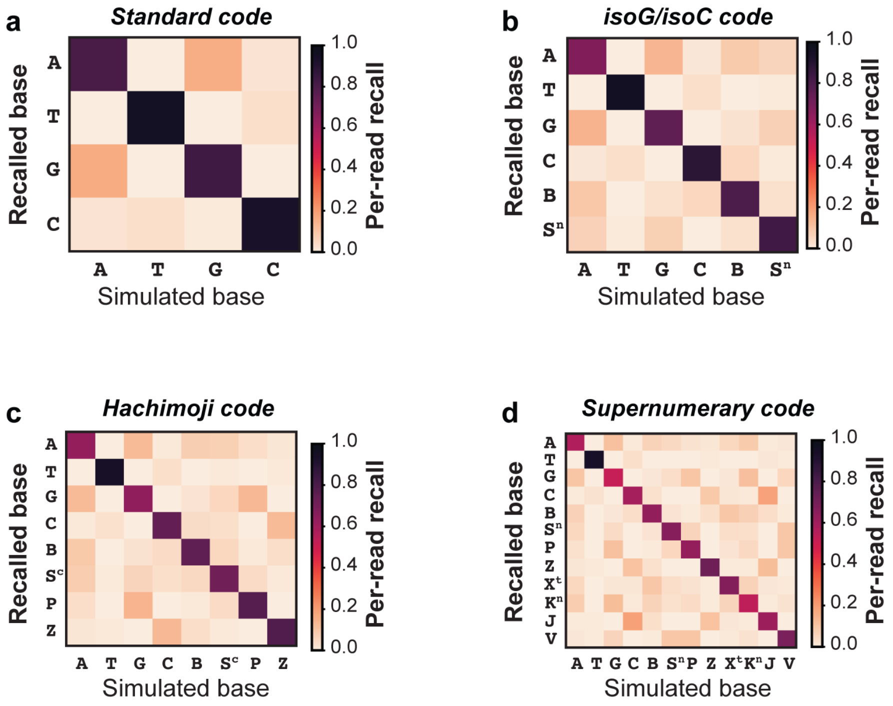

Supplementary Figure 31. Confusion matrix values of per-read recall for simulated reads. Confusion matrices showing per-read recall of simulated reads for; (**a**) a standard genetic code (A, T, G, C); (**b**) a theoretical isoG/isoC code (A, T, G, C, B, S^n); (**c**) the hachimoji code (A, T, G, C, B, S^c, P, Z); and iv); and (**d**) the 12-base S^nupernumerary code (A, T, G, C, B, S^n, P, Z, X^t, K^n, J, V). Recall was calculated from a simulation of n = 1,000 reads for each sequence (1,000 x 4,096 total reads simulated per base). Values shown as fractions. Legend shows color scale for fraction of simulated reads correctly recalled. Tabulated values shown in **Supplementary Table 27-30**.

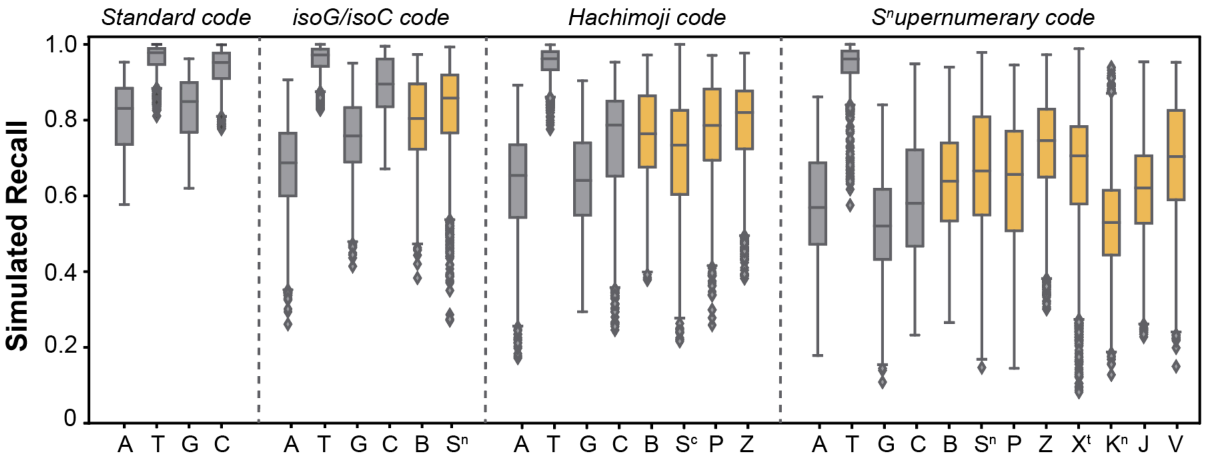

Supplementary Figure 32. Box plots of per-read recall for simulated reads. Box plots showing sequence-specific per-read recall of reads simulated in **Supplementary Fig. 31** that contain a genetic alphabet of increasing size: standard code (4 letters); isoG/isoC code (6 letters); hachimoji code (8 letters); and a S^nupernumerary code (12 letters with S = S^n). Boxplots show 25th and 75th percentiles (box), median (line), 10th and 90th percentile (whiskers), and outliers (points) for recall across all 4096 NNN<u>N</u>NNN sequences of each specified base (N = A, T, G, C only). Recall was calculated from a simulation of n = 1000 reads for each sequence (1000 x 4096 reads simulated per base). These simulations highlight how even theoretical basecall performance has a strong sequence dependence. Therefore, high recall is not guaranteed across all sequence spaces and certain sequence combinations are easier to distinguish from others.

Supplementary References

47. Roberts, C., Bandaru, R. & Switzer, C. Theoretical and experimental study of isoguanine and isocytosine: base pairing in an expanded genetic system. *J. Am. Chem. Soc.* **119**, 4640–4649 (1997).
48. Switzer, C. Y., Moroney, S. E. & Benner, S. A. Enzymatic recognition of the base pair between isocytidine and isoguanosine. *Biochem.* **32**, 10489–10496 (1993).
49. Singh, I. *et al.* Structure and biophysics for a six letter DNA alphabet that includes imidazo[1,2-a]-1,3,5-triazine-2(8H)-4(3H)-dione (X) and 2,4-diaminopyrimidine (K). *ACS Synth. Biol.* **6**, 2118–2129 (2017).
50. Hoshika, S. *et al.* "Skinny" and "fat" DNA: two new double helices. *J. Am. Chem. Soc.* **140**, 11655–11660 (2018).
51. Behera, B., Das, P. & Jena, N. R. Accurate base pair energies of Artificially Expanded Genetic Information Systems (AEGIS): clues for their mutagenic characteristics. *J. Phys. Chem. B* **123**, 6728–6739 (2019).
52. García, P. B., Robledo, N. L. & Islas, Á. L. Analysis of non-template-directed nucleotide addition and template switching by DNA polymerase. *Biochem.* **43**, 16515–16524 (2004).
53. Fiala, K. A. *et al.* Mechanism of template-independent nucleotide incorporation catalyzed by a template-dependent DNA polymerase. *J. Mol. Biol.* **365**, 590–602 (2007).
54. Matsuura, M. F. *et al.* Assays to detect the formation of triphosphates of unnatural nucleotides: application to *Escherichia coli* nucleoside diphosphate kinase. *ACS Synth Biol* **5**, 234–240 (2016).
55. Bauer, R. J. *et al.* Comparative analysis of the end-joining activity of several DNA ligases. *PLoS One* **12**, e0190062 (2017).
