# Supplementary material for: Enzymatic synthesis and nanopore sequencing of 12-letter supernumerary DNA
Source: Nat Commun. 2023 Oct 26;14:6820. doi: 10.1038/s41467-023-42406-z (PMC10603101; doi:10.1038/s41467-023-42406-z)
Supplement: Supplementary file 3 — Description of Additional Supplementary Files Document [file 41467_2023_42406_MOESM3_ESM.pdf]

Description of Additional Supplementary Files

Supplementary Data 1: Kmer values for XNA models. Each DNA and XNA-containing kmer used in this work, as well as its corresponding coverage, mean (KDE and observed), median, min, max, and standard deviation are listed.

Supplementary Data 2: Library oligonucleotide sequences. DNA oligonucleotide hairpin sequences used to build the models (NNN_hairpin_library) and validation datasets (val20_hairpin_library) in this work are listed in a format easily adaptable for IDT ordering. In addition to these hairpin sequences, sequence combinations built through XNA tailing and ligation for the validation dataset are listed (validation_sequences).
