# Supplementary material for: Enzymatic synthesis and nanopore sequencing of 12-letter supernumerary DNA
Source: Nat Commun. 2023 Oct 26;14:6820. doi: 10.1038/s41467-023-42406-z (PMC10603101; doi:10.1038/s41467-023-42406-z)
Supplement: Supplementary file 6 — Reporting Summary [file 41467_2023_42406_MOESM6_ESM.pdf]

nature portfolio

Corresponding author(s): Jorge A Marchand

Last updated by author(s): 2023/09/19

Reporting Summary

Nature Portfolio wishes to improve the reproducibility of the work that we publish. This form provides structure for consistency and transparency in reporting. For further information on Nature Portfolio policies, see our Editorial Policies and the Editorial Policy Checklist.

Statistics

For all statistical analyses, confirm that the following items are present in the figure legend, table legend, main text, or Methods section.

n/a	Confirmed	
☐	☒	The exact sample size (*n*) for each experimental group/condition, given as a discrete number and unit of measurement
☐	☒	A statement on whether measurements were taken from distinct samples or whether the same sample was measured repeatedly
☒	☐	The statistical test(s) used AND whether they are one- or two-sided<br>*Only common tests should be described solely by name; describe more complex techniques in the Methods section.*
☒	☐	A description of all covariates tested
☒	☐	A description of any assumptions or corrections, such as tests of normality and adjustment for multiple comparisons
☐	☒	A full description of the statistical parameters including central tendency (e.g. means) or other basic estimates (e.g. regression coefficient) AND variation (e.g. standard deviation) or associated estimates of uncertainty (e.g. confidence intervals)
☒	☐	For null hypothesis testing, the test statistic (e.g. *F*, *t*, *r*) with confidence intervals, effect sizes, degrees of freedom and *P* value noted<br>*Give P values as exact values whenever suitable.*
☒	☐	For Bayesian analysis, information on the choice of priors and Markov chain Monte Carlo settings
☒	☐	For hierarchical and complex designs, identification of the appropriate level for tests and full reporting of outcomes
☒	☐	Estimates of effect sizes (e.g. Cohen's *d*, Pearson's *r*), indicating how they were calculated

Our web collection on statistics for biologists contains articles on many of the points above.

Software and code

Policy information about availability of computer code

Data collection	guppy version 6.1.5+446c355 (ONT); minknow version 22.10.10 (ONT); tombo version 1.5.1 (ONT)
Data analysis	all code deposited on github (https://github.com/xenobiolab/xenomorph) with corresponding package release (doi: 10.5281/zenodo.8356450)

For manuscripts utilizing custom algorithms or software that are central to the research but not yet described in published literature, software must be made available to editors and reviewers. We strongly encourage code deposition in a community repository (e.g. GitHub). See the Nature Portfolio guidelines for submitting code & software for further information.

Data

Policy information about availability of data

All manuscripts must include a data availability statement. This statement should provide the following information, where applicable:

- Accession codes, unique identifiers, or web links for publicly available datasets
- A description of any restrictions on data availability
- For clinical datasets or third party data, please ensure that the statement adheres to our policy

Raw reads, basecalls, and reference fasta for sequencing data collected and analyzed in this work has been deposited in the SRA under Bioproject PRJNA932328.

Research involving human participants, their data, or biological material

Policy information about studies with human participants or human data. See also policy information about sex, gender (identity/presentation), and sexual orientation and race, ethnicity and racism.

Reporting on sex and gender	N/A
Reporting on race, ethnicity, or other socially relevant groupings	N/A
Population characteristics	N/A
Recruitment	N/A
Ethics oversight	N/A

Note that full information on the approval of the study protocol must also be provided in the manuscript.

Field-specific reporting

Please select the one below that is the best fit for your research. If you are not sure, read the appropriate sections before making your selection.

☒ Life sciences ☐ Behavioural & social sciences ☐ Ecological, evolutionary & environmental sciences

For a reference copy of the document with all sections, see nature.com/documents/nr-reporting-summary-flat.pdf

Life sciences study design

All studies must disclose on these points even when the disclosure is negative.

Sample size	Sample size for individual experiments is listed in figure legend or provided in methods section.
Data exclusions	For all sequencing data analyzed, a standard read quality thresholding was used to filter out reads with a q-score <9 or a signal match score >3 (ONT-Tombo). For full 12-letter DNA (Scuper-12/Snuper-12), only reads that map to the full construct were analyzed to avoid fragments.
Replication	Chromatograms and gels shown are representative of at least 3 replicates unless otherwise explicitly stated. Sequencing data for model building was collected from individual runs and cross validated on replicate runs for QC.
Randomization	For model building - no randomization was used. Full dataset collections were processed and analyzed.<br>For validation library hairpin sequence design - a 3'-end 20mer sequence of ATGC bp was generated by randomly selecting 20 nucleotides (w/ replacement) using a uniform probability distribution. Generated sequences were passed through IDTs oligo calculator to ensure that resulting sequence would not contain secondary structure elements that disfavor hairpin formation.<br>For validation library building - the validation oligo pools were constructed by randomly selection two hairpin oligo pools to ligate. Selection was performed serially with no anticipated bias.
Blinding	Blinding was not used in this study, and was not relevant to the study design. All data was processed and analyzed computationally with models described in work, and therefore treated equally. No means for tuning models were used by authors that could influence outcome of results.

Reporting for specific materials, systems and methods

We require information from authors about some types of materials, experimental systems and methods used in many studies. Here, indicate whether each material, system or method listed is relevant to your study. If you are not sure if a list item applies to your research, read the appropriate section before selecting a response.

Materials & experimental systems

n/a	Involved in the study
☒	☐ Antibodies
☒	☐ Eukaryotic cell lines
☒	☐ Palaeontology and archaeology
☒	☐ Animals and other organisms
☒	☐ Clinical data
☒	☐ Dual use research of concern
☒	☐ Plants

Methods

n/a	Involved in the study
☒	☐ ChIP-seq
☒	☐ Flow cytometry
☒	☐ MRI-based neuroimaging
